# Supplementary material for: Synaptic and neural behaviours in a standard silicon transistor
Source: Nature. 2025 Mar 26;640(8057):69–76. doi: 10.1038/s41586-025-08742-4 (PMC11964925; doi:10.1038/s41586-025-08742-4)
Supplement: Supplementary file 1 — Supplementary Information [file 41586_2025_8742_MOESM1_ESM.docx]

**Table of Contents**

[Supplementary Note 1: Definitions on neuro-synaptic behaviour and neural mimicking processes 4](#_Toc178693846)

[Supplementary Figure 1 | Neurons and synapses: roles and fundamental characteristics 4](#_Toc178693847)

[Supplementary Table 1 | Figures of merit of neuron-mimicking devices and technologies 7](#_Toc178693848)

[Supplementary Note 2: Parasitic bipolar firing and applications in submicron MOSFETs 9](#_Toc178693849)

[Supplementary Figure 2 | Hysteretic punch-through avalanche in 180 nm MOSFET 11](#_Toc178693850)

[Supplementary Figure 3 | Hysteretic punch-through avalanche in 500 nm MOSFET 12](#_Toc178693851)

[Supplementary Figure 4 | I-V characteristics of standard 500 nm transistor under nominal conditions (grounded bulk) 13](#_Toc178693852)

[Supplementary Figure 5 | Effect of the die’s substrate spreading resistance on the punch through characteristic of 180 nm length transistors 14](#_Toc178693853)

[Supplementary Figure 6 | Bulk-to-ground resistance control with an additional transistor 15](#_Toc178693854)

[Supplementary Figure 7 | Punch through control via substrate bias network with a control transistor 16](#_Toc178693855)

[Supplementary Figure 8 | Dynamics of punch-through impact ionization process in 180 nm transistors 17](#_Toc178693856)

[Supplementary Figure 9 | Sweep rate dependence of firing and relaxation voltages 18](#_Toc178693857)

[Supplementary Note 3: Physics based simulations of the floating bulk 2-transistor neuron device under experimental conditions 19](#_Toc178693858)

[Supplementary Fig. 10 | TCAD modelling results of the fundamental 180 nm transistor structure 19](#_Toc178693859)

[Supplementary Fig. 11 | ON and OFF transitions of the punch-through impact ionization firing mechanism 19](#_Toc178693860)

[Supplementary Fig. 12 | Hole density in the floating bulk transistor 20](#_Toc178693861)

[Supplementary Fig. 13 | ON transition voltage dependence on VG2 (RB) 21](#_Toc178693862)

[Supplementary Fig. 14 | TCAD transient simulations of sweep rate dependent hysteresis loops 23](#_Toc178693863)

[Supplementary Figure 15 | Device-to-device robustness of the firing process under punch-through conditions in 180 nm channel length transistors 24](#_Toc178693864)

[Supplementary Figure 16 | Cycle-to-cycle variability of the firing characteristics under high resolution acquisition for 500 nm channel length transistors 25](#_Toc178693865)

[Supplementary Figure 17 | Pulsed regime robustness of the firing mechanism under punch-through conditions in 180 nm channel length transistors 26](#_Toc178693866)

[Supplementary Figure 18 | Measurements of the firing time for different voltage spike amplitude in 180 nm channel length transistors 27](#_Toc178693867)

[Supplementary Figure 19 | Measurements of the firing time under single voltage spike in 180 nm channel length transistors 28](#_Toc178693868)

[Supplementary Figure 20 | Measurements of the firing time under single voltage spike in 500 nm channel length transistors 29](#_Toc178693869)

[Supplementary Figure 21 | Measurements of the relaxation dynamics of the neural behaviour in 180 nm transistors 30](#_Toc178693870)

[Supplementary Figure 22 | Spiking frequency dependent firing in 180 nm floating bulk neuron 31](#_Toc178693871)

[Supplementary Figure 23 | Pulsed synaptic update procedure and statistics 32](#_Toc178693872)

[Supplementary Figure 24 | Pulsed synaptic reset and variability 33](#_Toc178693873)

[Supplementary Figure 25 | Retention measurements under long-term periodic read 34](#_Toc178693874)

[Supplementary Figure 26 | Retention of the synaptic update in 500 nm length transistors 35](#_Toc178693875)

[Supplementary Note 4: physical origin of long-term synaptic update in floating bulk MOSFETs 36](#_Toc178693876)

[Supplementary Figure 27 | Device conditions during reset sweeps 36](#_Toc178693877)

[Supplementary Figure 28 | Retention measurements with grounded bulk of the synaptic device after set/reset in floating bulk condition 38](#_Toc178693878)

[Supplementary Note 5: Reliability aspects related to drain voltages under pulsed operation 39](#_Toc178693879)

[Supplementary Note 6: Tuneable frequency bursting neuron and its application in neural networks 41](#_Toc178693880)

[Supplementary Fig. 29 | Experimental testbench for floating bulk transistor in tuneable bursting neuron mode 41](#_Toc178693880)

[Supplementary Fig. 30 | Oscillatory spiking behaviour of tuneable floating bulk neuron 42](#_Toc178693881)

[Supplementary Fig. 31 | Range of firing frequencies across the tuning space and input excitatory currents 42](#_Toc178693882)

[Supplementary Fig. 32 | Schematic representation of a potential neural network section implemented with floating bulk transistor neurons and synapses 43](#_Toc178693883)

[Supplementary Fig. 33 | Detail of the control circuits used for dual inference/write procedures 44](#_Toc178693884)

[Supplementary Figure 34 | Device level SPICE modelling through fundamental phenomena 45](#_Toc178693885)

[Supplementary Note 7: Neuro-synaptic mimicking phenomena and devices, and their application in hardware-based demonstrations of ANNs 46](#_Toc178693886)

[Supplementary Table 2. Technologies used for neuron and synaptic mimicking 47](#_Toc178693887)

[Supplementary Note 8: Physical simulations of the mechanisms governing the floating bulk device 48](#_Toc178693888)

[Supplementary Figure 35 | TCAD simulation workbench and parameters 48](#_Toc178693889)

[Supplementary Figure 36 | Agreement of TCAD impact ionization simulations with experimental data 49](#_Toc178693890)

[References for the supplementary information 50](#_Toc178693891)

### **Supplementary Note 1: Definitions on neuro-synaptic behaviour and neural mimicking processes**

*Neural behaviour (integrate and fire models and higher order complexity)*

Neurons are the core information-processing elements in biological systems. Fundamentally, they are electrically non-linear cores that display tens of different dynamical behaviours (time-domain response based on history of the unit and immediate inputs) when driven by electrochemical stimulation^1^. Electrically mimicking their function is demanding with classical circuit elements (such as transistors) because of the degree of complexity and non-linearity that neurons exhibit under different conditions, typically requiring multiple devices to approach a fundamental neuron function. Many of the known neural dynamics have been modelled mathematically with fundamental dynamical system theory, where a set of first order differential equations and driving variables (input and output membrane voltages) define the state of the system through time.

The fundamental role of a neuron within a neural network is to integrate a set of input excitation signals through time and generate an according propagation signal at its output, to the next layer of neurons in the system. This fundamental first order behaviour is known as integrate and fire (IF), and it is electrically equivalent to a threshold firing hysteretic loop (see Supplementary Fig. 1a). To include an additional history term, in the dynamics of a first order neuron, the inclusion of a “leaky” term is often used to model the return of a neuron to its quiescent state when no input excitations are received or these are very mild, due to the diffusion of ions through the membrane of the cell^2^.


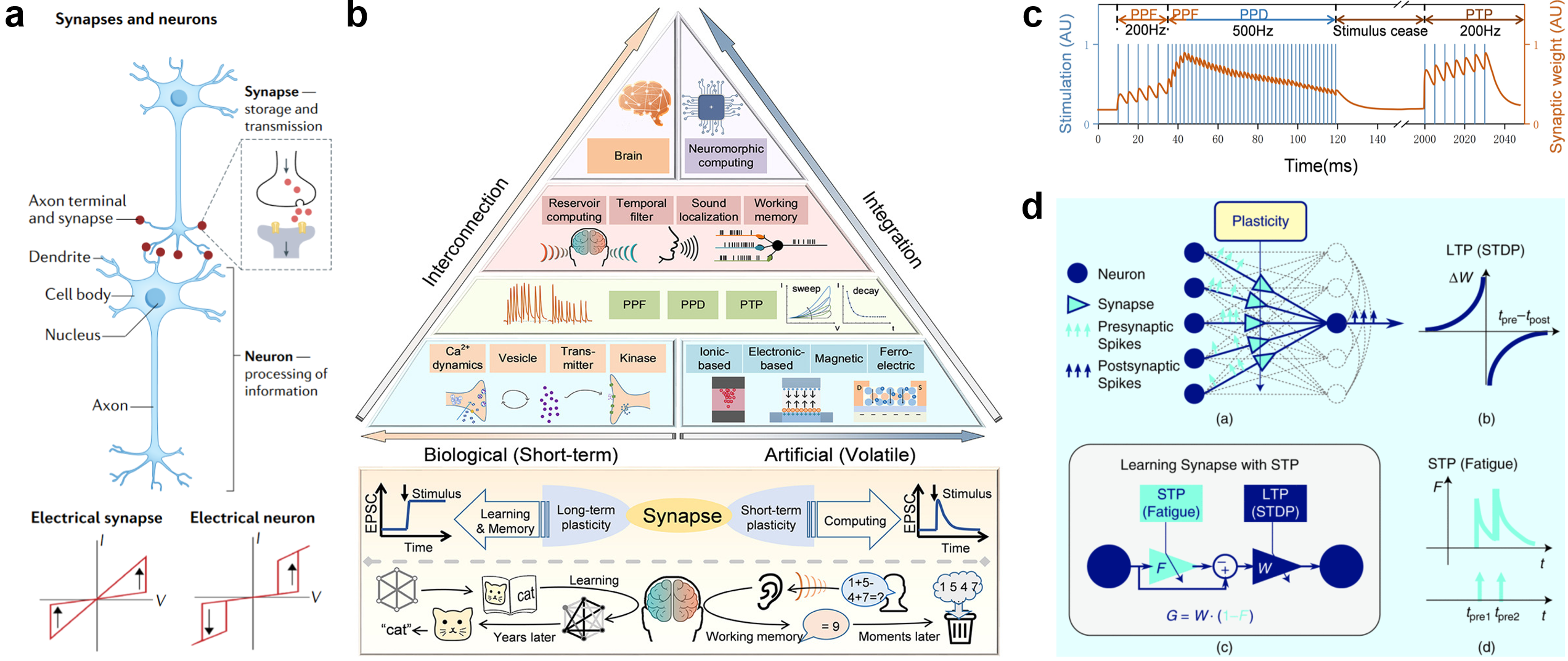


**Supplementary Figure 1 | Neurons and synapses: roles and fundamental characteristics. a,** A basic schematic representation of two neurons interconnected through a set of synapses (inset), and the simplified I-V electrical characteristics of each (bottom). Reproduced with permission from Ref. ^1^. Copyright 2022, Nature Publishing Group. **b,** A pyramidal representation of short-term synaptic behaviours in biological functions and neuromorphic circuits (top). A basic distinction between long and short-term plasticity in synapses and their role in specific functions of learning, computing and memorizing features within a neural network (bottom). Reproduced with permission from Ref. ^3^. Copyright ELSEVIER, 2023. **c,** Short-term synaptic weight potentiation (facilitation) and depression as a function of input stimulation (not decay when stimulus cease). Reproduced with permission from Ref. ^3^. Copyright 2023, ELSEVIER. d, (Top) A typical representation of a neural network featuring neuron (circles) layers and plastic synapses (lines), alongside a typical LTP characteristic as a result of spike timing (also known as spike timing dependent plasticity or STDP). (Bottom) A synapse block-level representation comprised by a STP portion and an LTP portion, alongside a typical STP weight versus time characteristic after spiked stimulation. Reproduced with permission from Ref. ^4^. Copyright IEEE, 2018.

Adding at least on more adaptation variable to the neural model enables a second order of dynamic behaviours that have been experimentally observed: particularly, the capability of a neuron to display a steady state of spikes in their action potential whose frequency depend on a constant input current stimulus, also known as spike-frequency adaptation or “bursting” ^5^. This dynamic is fundamental in biological systems for information coding and transmission through layers of neurons, and their abnormality has been linked, in neuroscience, to neurological disorders^6^. In this regard, multiple organic devices and electrochemical transistors are being researched to closely mimic the biological behaviour of neurons ^7^. Electrochemical transistors show great promise of achieving this. For example, polymeric transistors have shown that, coupled with additional circuitry, they can mimic the neuron model that more closely resembles the ionic processes giving raise to neuron’s membrane action potentials, the Hodgkin-Huxley model. However, in order to reach biological bursting frequencies, they require very large capacitances (> 100 nF) and very large currents (>10 μA)^8^, on devices that are difficult to scale due to the need of liquid ionic gates. In contrast, the floating bulk transistor is capable of a wide range of adaptive action potential bursts with smaller involved capacitances, much smaller currents and a robust integration capability, which can enable biological mimicking with standard technologies (see Supplementary Note 5).

A wide range of spiking regimes have been observed from biological neurons, and some state-of-the-art neuromorphic microchips include complex CMOS circuits capable of providing configurable neuron models and adaptability in the same circuit, depending on the type of neural network being built (such as Intel’s Loihi2)^9^. In that sense, the capabilities of the floating bulk transistor to show high degree of adaptability offers an exciting opportunity to reduce the area overhead of such circuits by exploiting avalanche dynamics and in-device capacitance effects. Meanwhile, higher order complexity neural behaviours, such as chaotic oscillations, are being explored with emerging devices (such as memristors^1^) but their application is still far down the line.

*Long-term synaptic potentiation or plasticity (LTP)*

LTP represents the permanent^†^ change in synaptic strength that is closely associated with learnt features and processes, a.k.a. memory (see Supplementary Fig. 1b). Biologically speaking, the idea that learning results from changes in the strength of the synapse was first suggested by Santiago Ramon y Cajal around 1894 and the modulation of synaptic connectivity was incorporated into models by Hebb in the 1950s (ref. ^3^). In neuroscience, the phenomenon of LTP was discovered in the hippocampus around the mid-70’s, but not experimentally demonstrated until 30 years later^10^. Even though the current understanding of long-term memory is relating it to a wide variety of different neural processes, there is general agreement on the fundamental role of LTP. From the perspective of artificial neural networks, the concept of LTP has become centric in the development of machine learning systems. As the fundamental element at the heart of artificial neural networks, the concept of LTP enables the offline and online training of large neural network models in software, traditional digital hardware (e.g. GPUs) and, more recently, dedicated neuromorphic hardware based on standard^11^ and emerging^12,13^ technologies.

The fundamental process of LTP is the increase (or decrease, depression, LTD) in the connection strength between two or more neurons through an axon (Supplementary Fig. 1b). Such change in weight, as it is typically called in machine learning, is driven by pre- and post-synaptic signals incoming from surrounding neural layers. Therefore, the LTP behaviour is dynamic in the sense that it could be reversed with certain excitation, providing the system with the capability to update a learnt process or even forget it altogether. Neuromorphic circuits typically implement this characteristic as a non-volatile memory which can be digital or analogue in nature, in the sense that it can be computationally represented by a binary number (of several bits) or electrically as a variable conductance, which is the most closely related to the biological phenomenon (considering that neural signals are in essence ionic currents).

*^†^Note that the term “permanent” is usually employed to refer to a long-term process, but LTP it is not necessarily perpetual neither from the biological principle nor from the application sense. Some literature even use the term between quotes to reflect this matter* ^3^*[Li2023iScience].*

*Short-term synaptic plasticity*

Compared to LTP forming the foundation of learning and memory, short-term plasticity (STP) is essential for critical computation functions. The key difference relies on STP being characterized as relatively slow fading dynamic of the weight update in a synapse: when no stimulation is present, the synapse tends to return to an equilibrium weight or quiescent state (see Supplementary Fig. 1c) While practical applications of LTP are centric to state-of-the-art neural networks implemented in neuromorphic accelerator cores^14,15^, the implementation of STP in hardware is still elusive from the device perspective and, more importantly, from its active use in application-specific circuits that mimic biological functions that are known to rely on such process^3^. From the perspective of biological neural processes, STP is expected to play a pivotal role in what is known as “working memory”^16^, which in other words could resemble a temporal buffer that unifies memory and computation, only to discard the incoming data moments after it has been processed. This kind of principle is widely used in the processing of time data series^17^, resembling biological processes such as sound recognition or spatial perception^3^.

Initially, around the late 90’s, the potential of using STP in machine learning tasks was not well understood, which could explain the scarce number of early reports involving the implementation of machine learning systems or routines including STP. Nevertheless, there was general agreement on its potential in time-domain processing and decision tasks^4^, and several studies started to emerge including this phenomenon. CMOS circuits have been used for decades to emulate STP, mainly driven by subthreshold circuits and integrator capacitors on-chip^18,19^. More recently, ionic dynamics in memristors or memtransistors have also been explored for their close similarity to ionic diffusion processes involved in biological synaptic dynamics, but their status is still infant in terms of integration feasibility and reliability^20^. Still, they are being explored for their use as synapses that transition from an STP into an LTP regime once certain excitatory thresholds are surpassed (see Supplementary Fig. 1d), or the application of STP dynamics in neuromorphic computation strategies based on perturbations, such as recurrent neural networks^17^ or reservoir computing^21^, where emerging devices (such as memristors) struggle due to inherent mechanism variability.

*Neuro-synaptic behaviour*

From the electrical modelling of neurons and synapses, it is possible to see that there are important similarities (recall Supplementary Fig. 1a): in both cases, the synapse or neuron show a dynamic change of conductance or membrane potential (respectively) according to its previous state and to the inputs that are received from their surroundings. For this reason, the electrical behaviour of LTP, STP and neurons (particularly in their integrate and fire model) are very closely related. Particularly for the case of STP and neuron firing/relaxation/inhibition, the time dependent update of the output within certain thresholds can determine whether the device is used in a synaptic regime or as a neuron element. This can be clearly observed in Supplementary Fig. 21 and is the main reason why neurons and synapses have been implemented, in many cases, with the same technology in different circuit configurations (such as phase change devices^22,23^ or ferroelectric FETs^24,25^).

**Supplementary Table 1 | Figures of merit of neuron-mimicking devices and technologies.**

Main figures of merit of neuron mimicking approaches at the electronic circuit level listed and compared to the floating bulk transistor approach. Only experimentally obtained results are included in this table, no simulation-based results or technology node extrapolations are included. Note that this list is not exhaustive, as multiple other approaches could be listed, including organic/molecular devices such as liquid and solid ionic gate transistors, or neuro-synaptic transistors built with novel 2D, perovskite, or MXene materials. Though the research in these directions is vast and promising, the technology readiness of such approaches is still low, hence we have intentionally avoided their mention here and only included technologies that are currently under production or in the production research stage, with much higher technology readiness than academic research devices. This does not intend to minimize the potential of novel approaches, but to clearly highlight how the use of the floating-bulk transistor as neuro-synaptic device positions amongst other currently available technologies. LIF, leaky integrate and fire; IF, integrate and fire; PCM, phase change material; NEMS, nano-electromechanical system; Z^2^-FET, zero-impact ionization FET; STT, spin transfer torque (a type of magnetic memory for memristors); L-FeFET, leaky-ferroelectric FET; PD-SOI, partially depleted silicon-on-insulator; T, transistor; M, memristor; C, capacitor; R, resistor; N/A, data not available; OTS, ovonic threshold switch; IMT, insulator-to-metal transition. SONOS, Silicon-oxide-nitride-oxide-silicon, as typical structure of embedded flash floating gate memories. ^*^The peak power in PCM is produced during neuron reset step.

| Function | Technology | Voltage | Time constant | Energy per spike /  peak power | Size | Device count | CMOS integrable? | Mature |
| --- | --- | --- | --- | --- | --- | --- | --- | --- |
| LIF neuron  ^26^ | Metal-oxide memristor | 1.25 V ~ 3 V | 5 μs to  50 ms | ~40 pJ | (0.053μm^2^ in 7 nm) | 1T1M | Device level | Med |
| LIF (spiking)  ^27,28^ | BTBT 45 nm  PD-SOI CMOS | 1.3 V ~  1.8 V | 5 μs to  1 ms | 6.7 fJ | 129 μm^2^ | > 8 T | Yes | High |
| LIF neuron  ^29^ | CMOS pseudoresistors | 1.2 V | 1.2 ms | N/A | 1392 μm^2^ | > 9T1C | Yes | High |
| LIF neuron  ^30^ | CMOS-NEMS | ~0.5 V | 1 μs | ~50 fJ | 896 μm^2^ | 10T2C  2NEMS | Yes | Low |
| IF neuron  ^31^ | Dual-overlapped gate MOSFET | 1.5 V | 1 μs | ~100 pJ | N/A | 9T1C | Possible | Med |
| IF neuron  ^32^ | Z^2^-FET  (tunnelling) | 2 V~  4 V | > 5 ms | N/A | 100 μm^2^ | 1T (FDSOI) | Possible | Med |
| IF neuron  ^33^ | PCM memristor | 1.5V~5.5 V | 40 μs ~ 200 μs | N/A  4.4mW^*^ | ~0.25μm^2^ | 17T1M | Yes | Med |
| Time-surf neuron^34^ | Memristor | 3.5 V | 100 μs ~ 1 ms | ~10 pJ | 1900 μm^2^ | 1TFT1M | No | Low |
| IF neuron  ^35^ | STT memristor | 1.2 V | 10 μs | N/A  (>1 mA) | 26 μm^2^ (in 65 nm) | 11T2M | Yes | Med |
| LIF neuron  ^36^ | L-FeFET | ~1.8 V | 10 ~  100 ms | N/A  ~6μW/μm | N/A  (6F^2^ min.) | 2T1R | Research | Low |
| LIF neuron  ^37–39^ | Vertical/stacked silicon nanowires or fins | ~6 V | 100 μs to 5 ms | N/A  2.5~20 μW | ~0.25 μm^2^ | 1T  (2 gate  tunning) | Possible (non-standard) | Med |
| IF neuron  ^40^ | OTS memristor | ~1 V | Capacitor- fixed | 1 pJ | (4F^2^ min.) | 1M1C | Yes | Med |
| IF neuron  ^41^ | IMT memristor | ~1.75 V | Capacitor- fixed | ~12 μW | N/A | 1T1M + 2R1C | Possible | Low |
| LIF neuron  ^42,43^ | PD-SOI | 3 V ~ 3.5 V | 50 ns ~ 1 μs | ~35 pJ  ~1.5 mW | 1.8 μm^2^  (1767F^2^) | 18T +  4C 3R | Yes | High |
| Neuron + synapse ^44^ | Floating gate (SONOS) on SOI | > 3 V | 2.5 ms to 25 ms | 70 pJ to  700 pJ | 0.24 μm^2^  (6F^2^) | 1T float. gate | Limited by  SONOS | Med |
| LIF neuron  ^45^ | Floating gate tri-gate (SONOS) | Up to  +/- 9 V | N/A  (~100 μs) | 0.62 pJ | N/A | 8T | Limited by  SONOS | Med |
| Tuneable neuro-synaptic  (this work) | MOSFET | 1.5 V ~ 4.5 V | 1 μs to  >> 1 s | ~415 pJ/μm  (~75 pJ min.)  ~ 18 μW/μm | ~0.2 μm^2^  (0.04μm^2^)  (6F^2^) | 1T or  2T tune | Yes (standard) | High |

### **Supplementary Note 2: Parasitic bipolar firing and applications in submicron MOSFETs**

As was depicted in Fig. 2a of the main text, the very structure of a MOSFET device comprises a parasitic bipolar transistor conformed in the silicon. In the case of an n-channel MOSFET, this parasitic device is the result of the subjacent N-P-N structure resulting from the source and drain regions separated by the gate length. From decades of semiconductor physics studies, the conduction regimes of this kind of structure have been well identified through the progression of different device technologies. Altogether, planar MOSFET devices typically show 3 main parasitic mechanisms that result in the rapid increase of the current flowing through the drain, beyond the control of the gate terminal: (i) the drain junction avalanche breakdown; (ii) the bipolar impact-ionization (snapback) mechanism; and (iii) the punch-through impact ionization mechanism. Given that the first is mostly related to the design parameters of the drain-body n-p junction, pure drain avalanche is typically an unwanted issue in high-voltage or power MOSFET devices, but it plays little role in sub-micrometer CMOS transistors. Snapback and punch-through, on the other hand, became dominant with the downscaling of device length and with the advent of alternative technologies to the bulk planar CMOS in the 90’s and early 2000’s, such as partially depleted SOI, fully depleted SOI and FinFET. Depending on the deepness of the scaling, the technology platform and the specific design of the front-end-of-line (FEOL, that is all the transistor parameters, such as doping profiles in the silicon, gate and spacer dielectrics, contact modules, etcetera), the dominant mechanism may be one or the other^46,47^, but it is well known that both mechanisms typically co-exist, since they are driven by a large number of common parameters and by the drain voltage itself.

Snap-back mechanism is a parasitic bipolar effect present in MOSFET devices that consists of the parasitic bipolar device turning on upon the presence of a sufficiently high drain (collector) voltage. When the bulk terminal is grounded, as in normal conditions for a traditional bulk-CMOS technology, this avalanche current gives rise to a potential drop across the intrinsic resistance of the bulk semiconductor itself, increasing the potential of the bulk terminal with respect to the source/emitter terminal.This voltage drops tends to turn on the parasitic bipolar NPN transistor (in a n-channel MOSFET), which is able to drive the high currents produced during the avalanche process and trigger the snap-back, reducing the drain voltage in the process and protecting sensitive devices connected to it from large voltage transients. Given that this is an avalanche driven phenomenon, the characteristic abruptness of the snap-back process has been useful in the development of electrostatic discharge (ESD) protection devices to protect the extremely sensitive ultra-thin oxides of MOSFET devices from damaging during different stages of the product life (BEOL fabrication, dicing/pick and place, testing, encapsulation, and off-factory manipulation). Their main characteristic is the capability of sinking large current transients produced during an ESD event, limiting the maximum voltage developed on the sensitive gate oxides of core devices. Popular structures for this purpose are known in the IC design community as gate-grounded or gate-coupled MOSFETs (GGNMOS, GCNMOS in the n-channel versions)^48,49^.

In sub-micrometer MOSFETs, punch-through is a parasitic bipolar effect that also results on the gate terminal losing control of the drain current due to deepening of the depletion region at the drain junction the drain. As the drain voltage is increased, the width of the quasi-neutral region of the bulk semiconductor under the channel (namely, the majority-carrier charge in the base of the parasitic bipolar transistor), is reduced. For a device with a short channel length and relatively light substrate doping, it does not take much increase in drain voltage before all the majority carrier base charge of the parasitic bipolar device is depleted: at this point, the drain (collector) punches through to the source (emitter). In this condition, the collector current can become excessively large, being limited only by the spreading resistance of the remaining neutral regions (source and drain) and the contact resistances at the source and drain terminals. The drain current at or close to the punch-through condition is no longer controlled adequately by the gate voltage for proper device operation, and for this reason device structure and doping are designed so that this condition is avoided during nominal device operation^47,50–52^.

After the parasitic bipolar transistor is in control of the current flowing between the drain and source terminals, when the drain stress voltage is reduced the device will eventually be turned off and the current will tend to return to its initial values (i.e., the off current of the transistor mainly defined by subthreshold diffusion current). However, to reach this condition, the carrier excess in the base of the bipolar transistor needs to be returned to the equilibrium level. This process has also received the name of single transistor latch (STL) when first observed in floating body devices in SOI technologies^53^, as the device gets “latched” into a state until a “reset” condition is reached. During this stage, the floating bias network connected to the bulk of the transistor effectively provides a current path and tunes the voltage drop in the parasitic bipolar structure to control the operating regime of the device.

MOSFETs operating under punch-through impact ionization regime have been proposed as abrupt turn-on transistors with subthreshold slopes below the fundamental quantum limit (60 mV/dec)^54^ and also as a capacitorless memory unit thanks to the hysteretic characteristics of their drain current as function of drain voltage (output curve, Id-Vd)^55,56^. Hysteresis in the output curve of short-channel transistors has been observed as early as 1987,(Ref. ^57^) however the phenomenon was only exploited as a memory effect around the late 2000’s with different approaches: junctionless transistors^58^, SiC or SiGe heterojunction transistors^59,60^, double-gated^58,61^ and gate all-around MOSFETs^55^ or floating gate nanowires^62^. Meanwhile, the feasibility of neuron-like behaviours exploiting these characteristics has been sporadically explored in the past^42,43,45,63–67^. Recent explorations, however, reduce to device level analysis based on simulations (mostly using finite element methods, such as technology computer aided design, or TCAD)^32,68–74^.

The most explored alternative to implement single-transistor neurons to the date involves technology specific implementations of single transistor latches, including SOI, Fin-like structures, stacked or vertical silicon nanowires^37–39,68^. In all cases the floating body of the device plays a central role, with slight differences in the conduction mechanism for different device architectures. A more developed approach uses a standard PD-SOI NMOS transistor in a band-to-band-tunnelling (BTBT) regime to act as an integrator element in LIF/spiking neurons^27,28^. In these devices, the bulk terminal is used as means for resetting the integrator element, but this requires several additional transistors (at least a threshold detector, all in all comprising more than 8 extra transistors for a single neuron). However, their performance is very much fixed (not tuneable or only tuneable through extended circuits), in some cases (as vertical nanowires or stacked nanosheets) their implementation is not fully compatible with standard, readily available CMOS processes, and their performance can be limited, involving high voltages (6 V), negative control voltages (V_G_ < 0), and limited characteristic time constants (100 μs ~ 5 ms) that are not suitable for multiple bio-mimicking functions (see Supplementary Table 1). Moreover, from an implementation perspective, the potential of the floating bulk MOSFET for neuromorphic applications has been specific to neuron model mimicking, mostly integrate and fire, by including the device as part of a larger circuit. Still, the synaptic capabilities have not been explored and the neural performance that can be achieved has not been clearly assessed. Simplified implementation of neural and synaptic behaviours with readily available, standard transistors can be an important boost towards the rapid development of neuromorphic integrated circuits without incurring into large cost/area overheads or into the challenges of relying on emerging technologies on the short term.

**
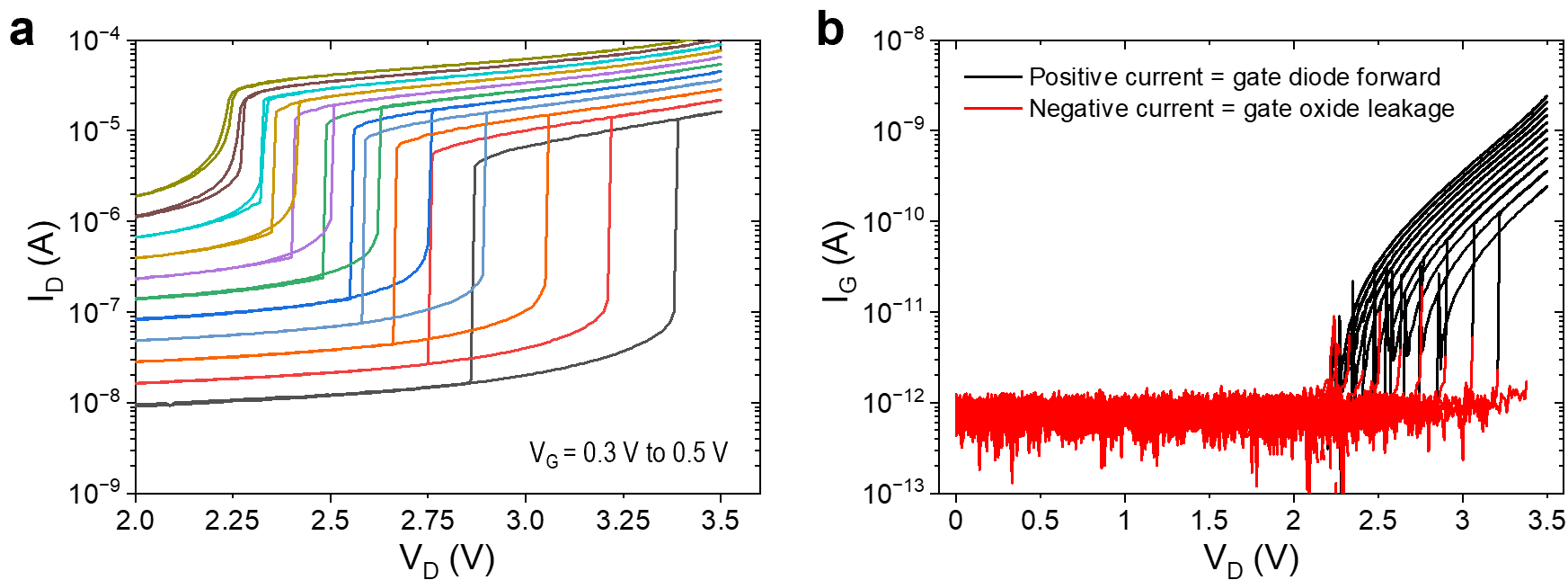
**

**Supplementary Figure 2 | Hysteretic punch-through avalanche in 180 nm MOSFET. a,** Output characteristic drain current I_D_ vs. drain voltage V_D_ of a standard 180 nm length transistor under a floating bulk condition. Transistor channel width is 1 µm. The body contact is floated from the ground node through the substrate spreading resistance of the die and an external transistor biased in the weak-inversion region with V_G2_ = 1.4 V (see Fig. 2d of the main text). V_G_ is stepped between 0.3 V and 0.5 V in 20 mV steps. Note that, for increasing V_G_, the response goes from hysteretic to the typical “kink” effect of body transistors. Voltage sweep rate is between 0.25 V/s and 0.75 V/s in auto-ranging mode. **b,** Gate current is monitored during the whole I_D_-V_D_ sweep to ensure the gate oxide is not being damaged. Colour denotes the sign of the current in the logarithmic scale, plotted as absolute value |I_G_|. Upon avalanche firing, the gate antenna protection diode clearly shows the increase in the bulk-related current (black lines). Gate leakage remains below 10 pA in all cases.


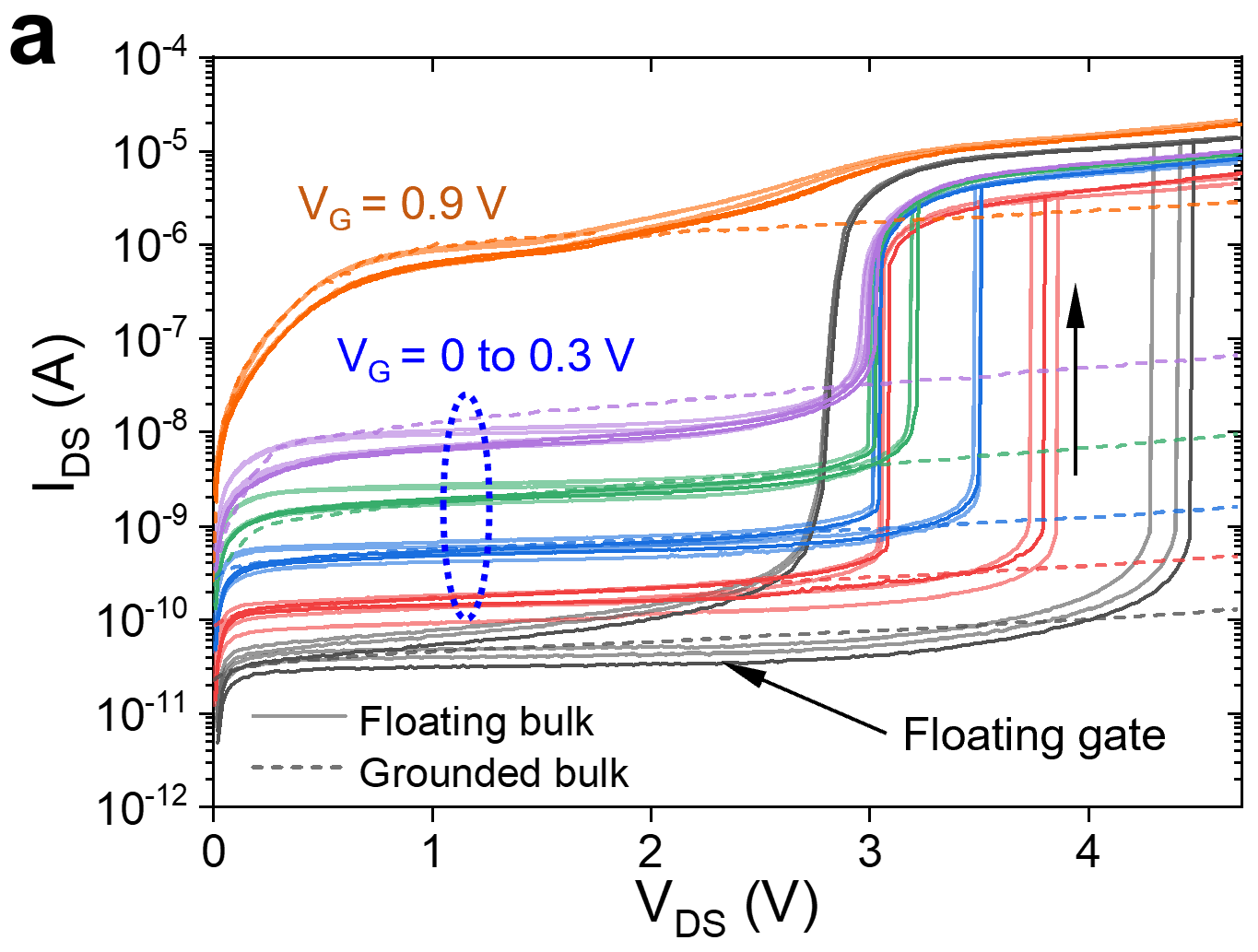


**Supplementary Figure 3 | Hysteretic punch-through avalanche in 500 nm MOSFET. a,** Output characteristic drain current I_D_ vs. drain voltage V_D_ of standard 500 nm length transistors (thick oxide device in a 180 nm technology node) under a floating bulk condition. Characteristic of three different devices are superimposed. Transistor channel width is 1 µm. The body contact is floated from the ground node through the substrate spreading resistance of the die and an external transistor biased in the weak-inversion region with V_G2_ = 1.4 V. V_G_ is stepped between 0 V and 0.3 V in 100 mV steps, while black and orange curves represent floating V_G_ and VG = 0.9 V, respectively. Note that, for increasing V_G_, the response goes from hysteretic to the typical “kink” effect of body transistors. Dashed lines show the response but with grounded bulk condition (through a pad connected to top-wafer body tap connections). Voltage sweep rate is between 0.25 V/s and 0.75 V/s in auto-ranging mode.


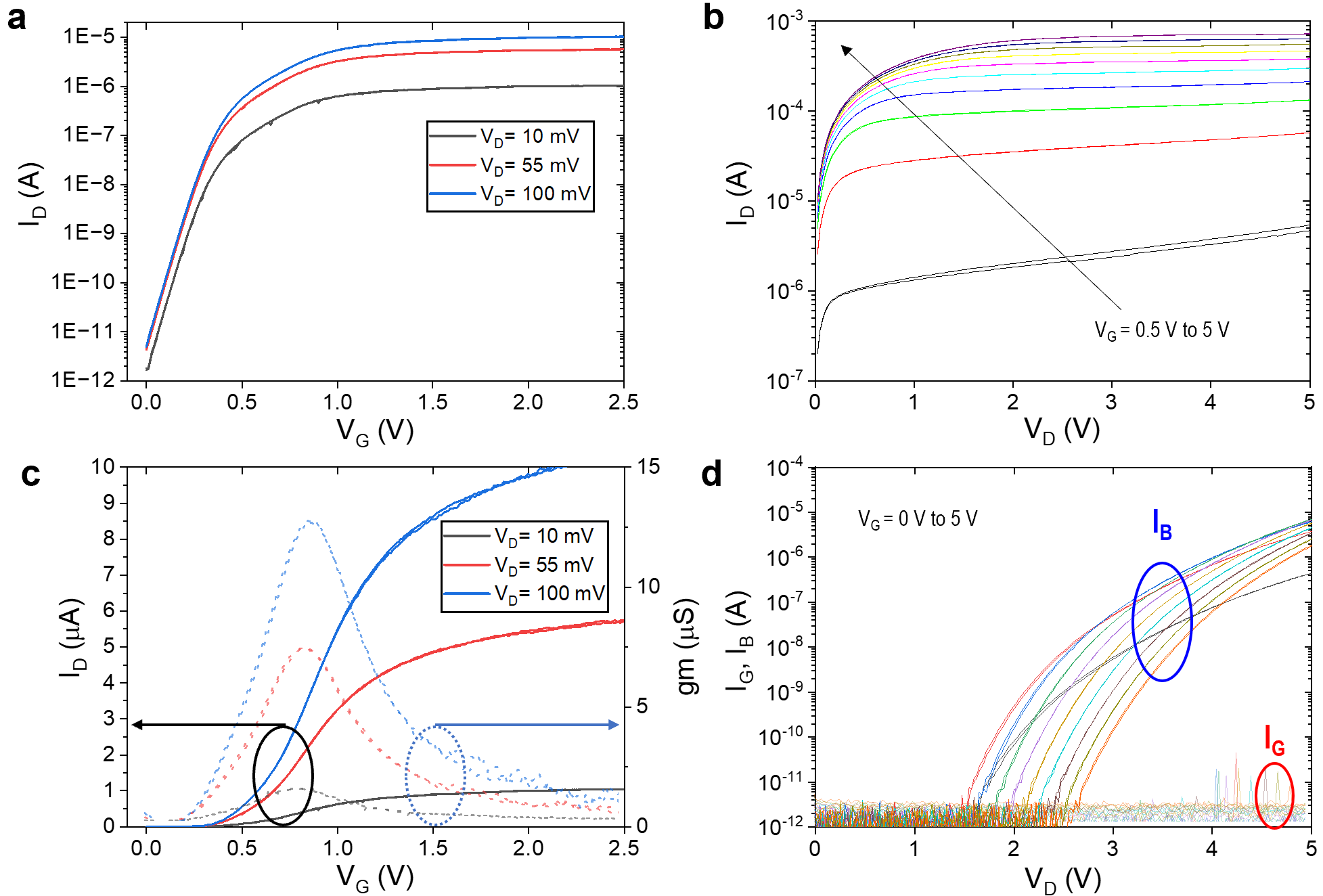


**Supplementary Figure 4 | I-V characteristics of standard 500 nm transistor under nominal conditions (grounded bulk). a,** Transfer characteristic drain current I_D_ vs. gate voltage V_G_ of standard 500 nm length transistors (thick oxide device in a 180 nm technology node) under nominal conditions (grounded bulk), for three different V_D_. **b,** Output characteristic I_D_ vs. V_D_ at various V_G_. **c,** Left axis is the same data from panel a, only in linear scale. Right axis (dashed curves) is the incremental transconductance (gm) of the same device. **d,** Gate (I_G_) and bulk (I_B_) currents monitored during the sweeps in panel **b**. Note that body current can exceed 7 µA, clear indicator of severe impact ionization. In all cases, voltage sweep rate is between 0.25 V/s and 0.75 V/s in auto-ranging mode.

**
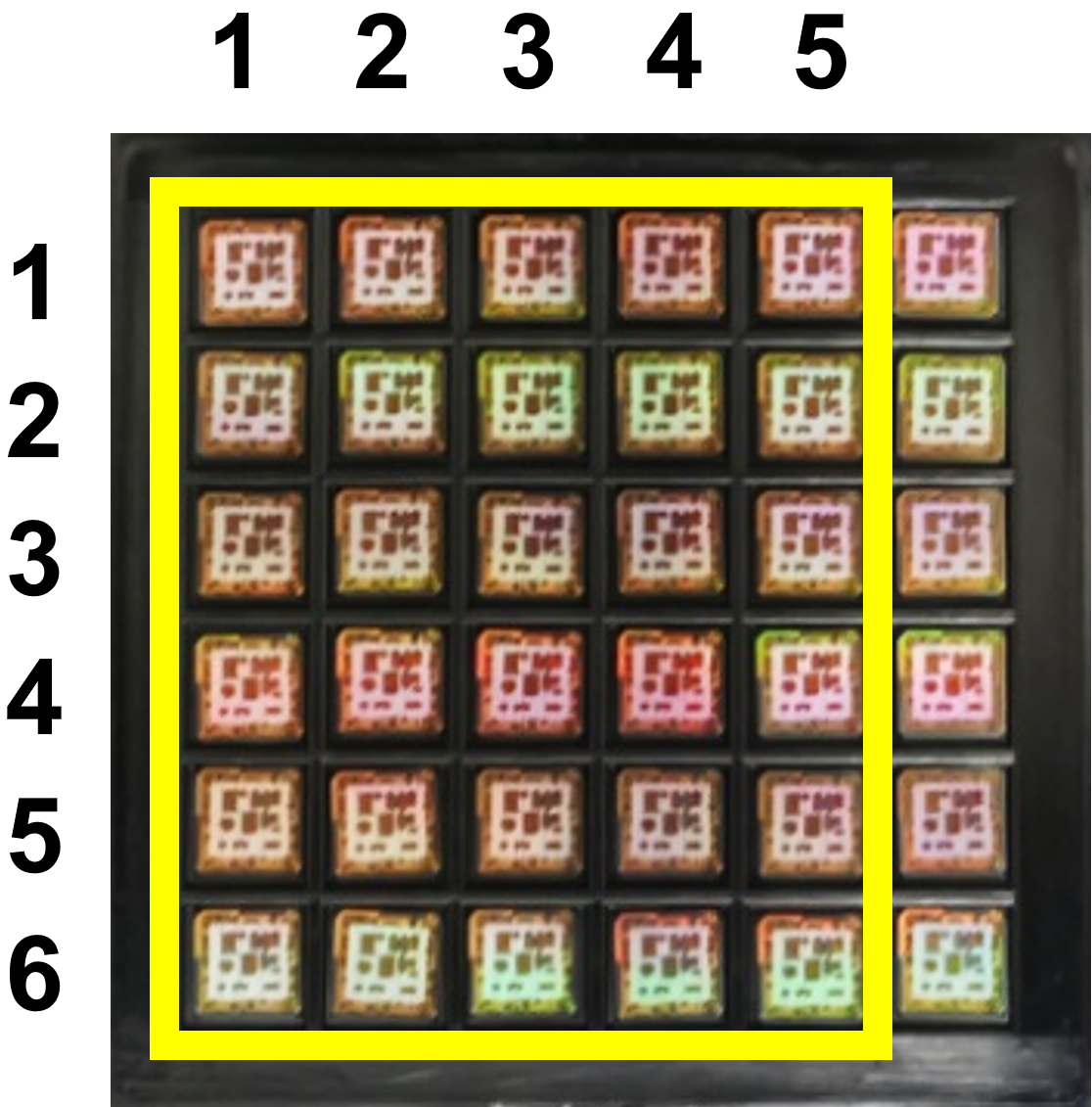
**

**
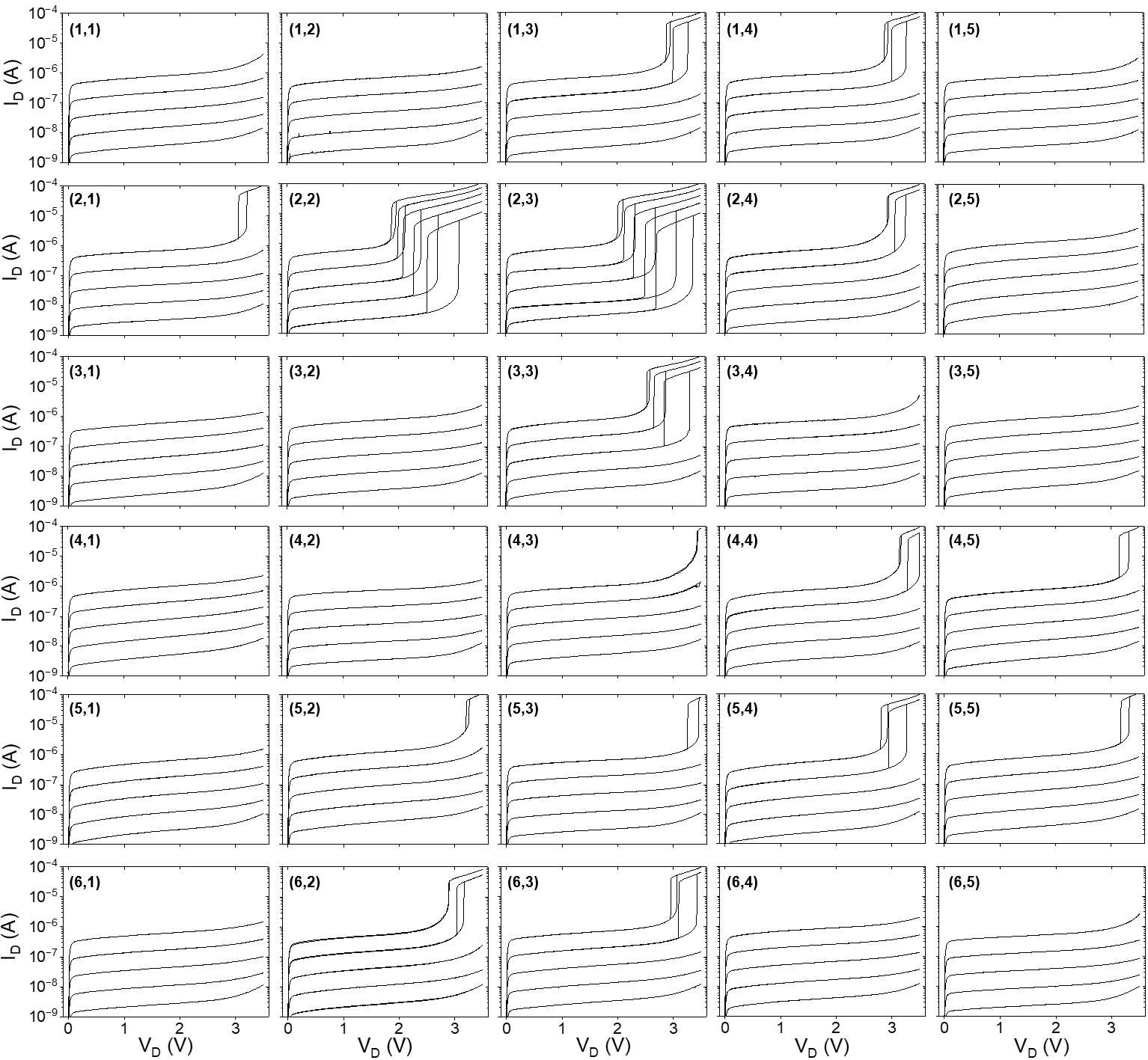
**

**Supplementary Figure 5 | Effect of the die’s substrate spreading resistance on the punch through characteristic of 180 nm length transistors.** Out of 30 dice (5 mm × 5 mm) from a typical waffle carrier (top), we observe die-to-die variability of the avalanche process when measuring devices grounded through the back wafer side (using the probe station chuck). The effect shows a degree of area correlation on the arrangement, which could be related to local gradients of wafer doping, impacting on die-to-die variability of the bulk spreading resistance.

**
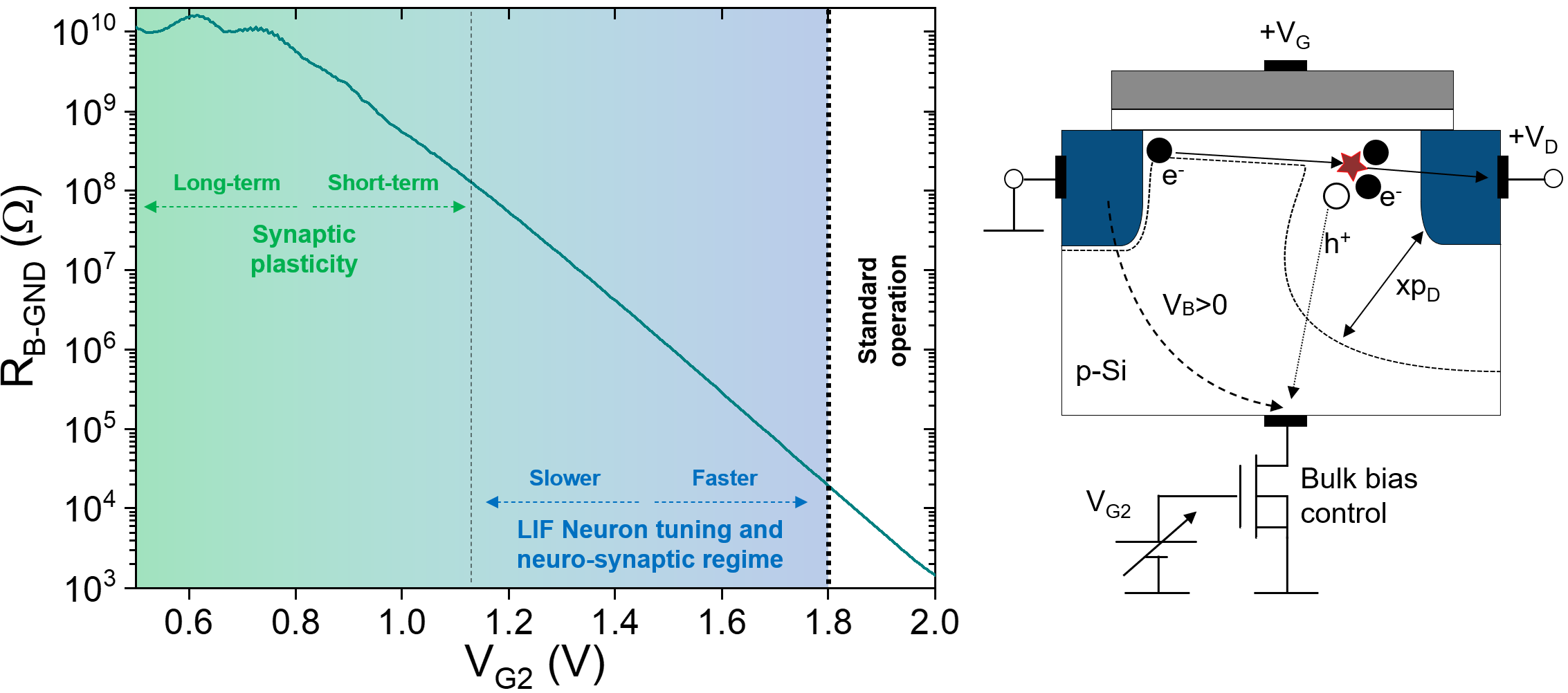
**

**Supplementary Figure 6 | Bulk-to-ground resistance control with an additional transistor.** Effective resistance imposed by the channel of the bulk bias control transistor as a function of applied V_G2_ bias (see schematic on the right-hand side). The resistance value is the extracted channel resistance of the employed BS170 transistor at low V_D_ = 100 mV as a function of V_G2_.

**
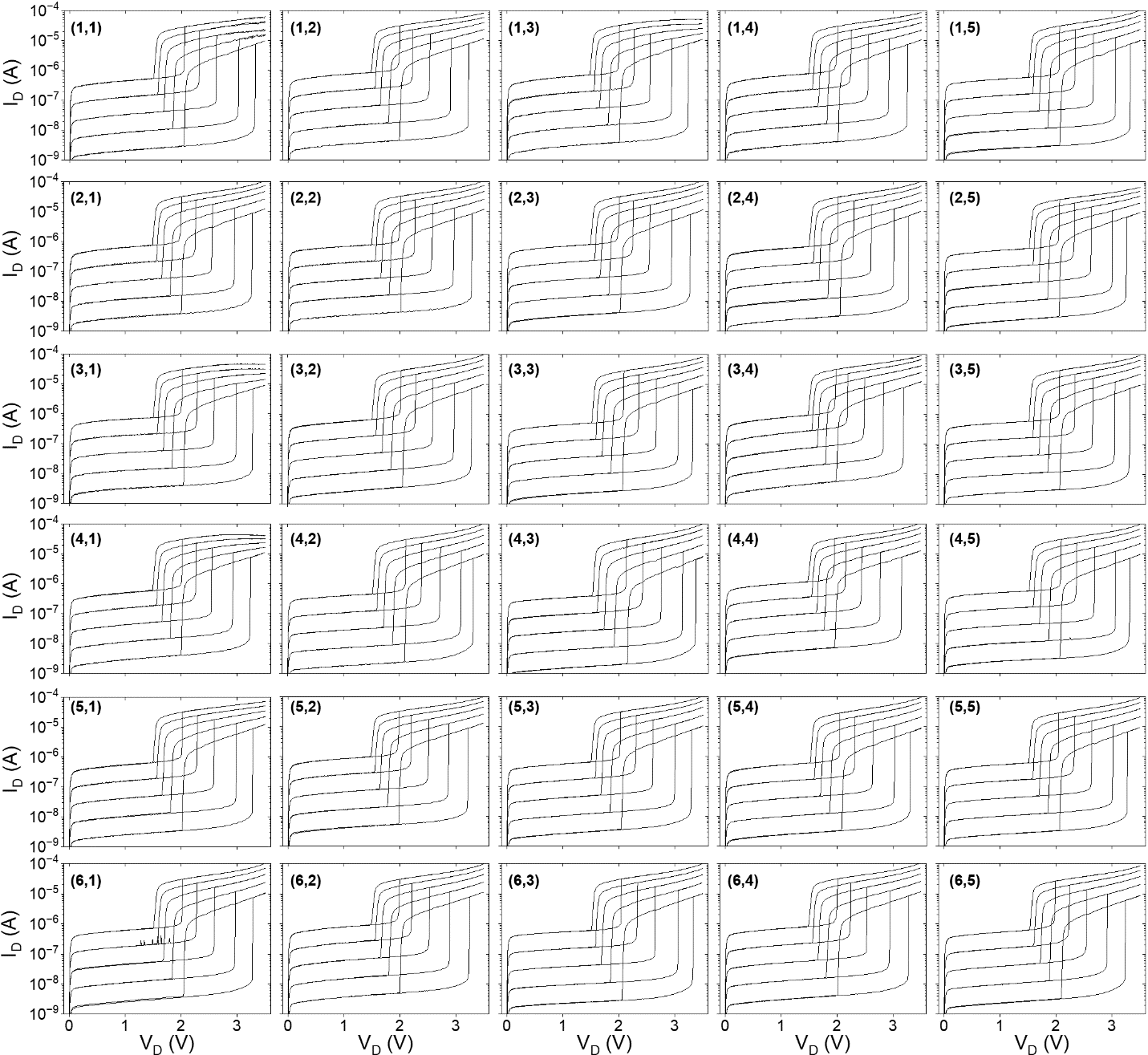
**

**Supplementary Figure 7 | Punch through control via substrate bias network with a control transistor.** Across the same 30 dice from Supplementary Fig. 5, we observe consistent punch through characteristic in all of them when using an external transistor connected to the back wafer side (through the probe station chuck). In all cases, gate bias of the control transistor (V_G2_ in Fig. 1d of the main text) is 1.3 V. This effectively masks the die-to-die variability of the spreading resistance of the substrate and allows to tune the neuro-synaptic characteristic with a 100 % yield. Remaining variability is inherent to the CMOS fabrication process and to the repeatability on the landing of the probe tips.


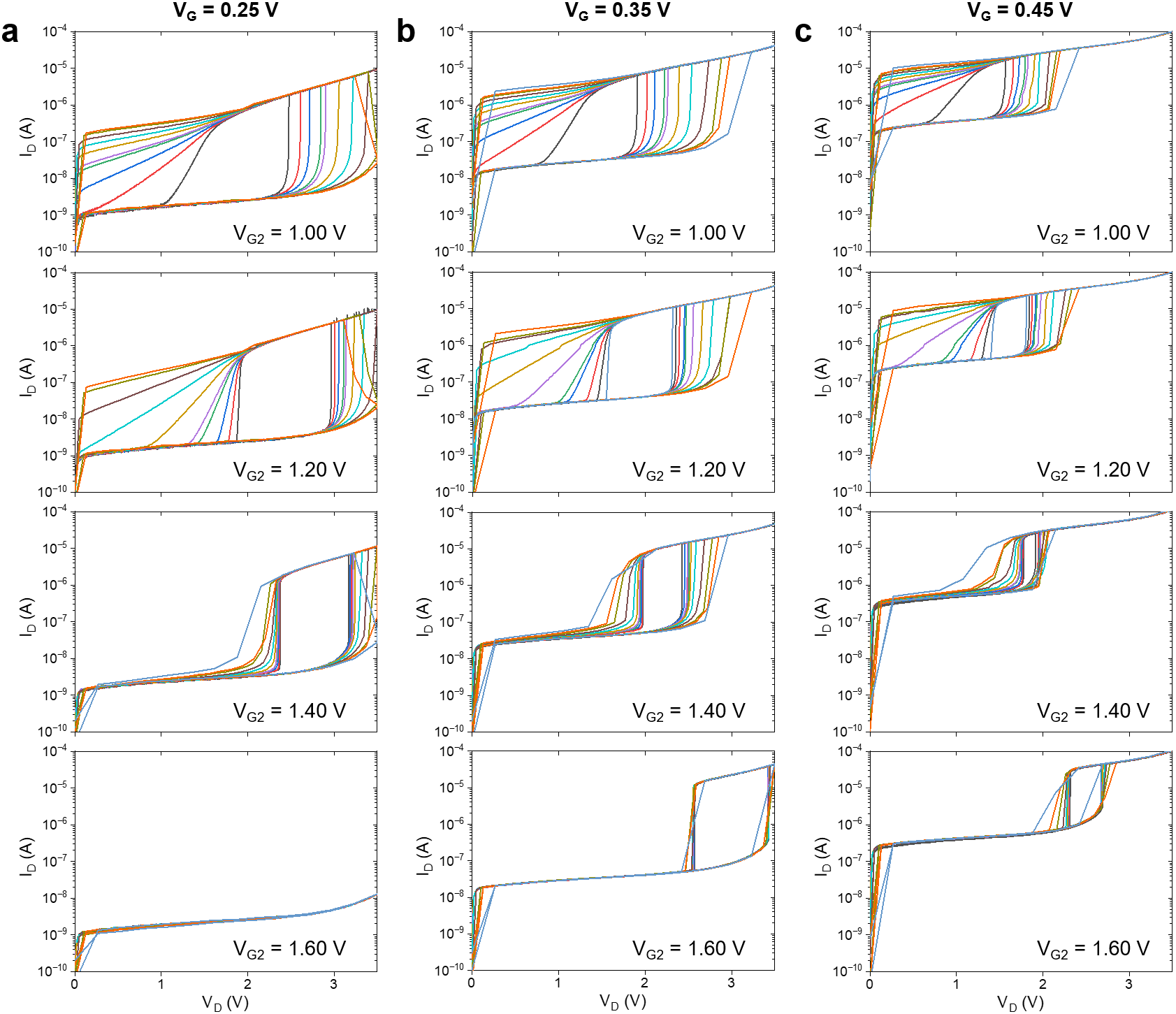


**Supplementary Figure 8 | Dynamics of punch-through impact ionization process in 180 nm transistors.** I-V Sweep rate dependence of the firing (V_fire_) and relaxation (V_relax_) voltages measured at V_G_ = 0.25 V (column **a**), V_G_ = 0.35 V (column **b**), and V_G_ = 0.45 V (column **c**), and with V_G2_ ranging between 1.0 V and 1.8 V. See detailed discussion in Supplementary Note 3.


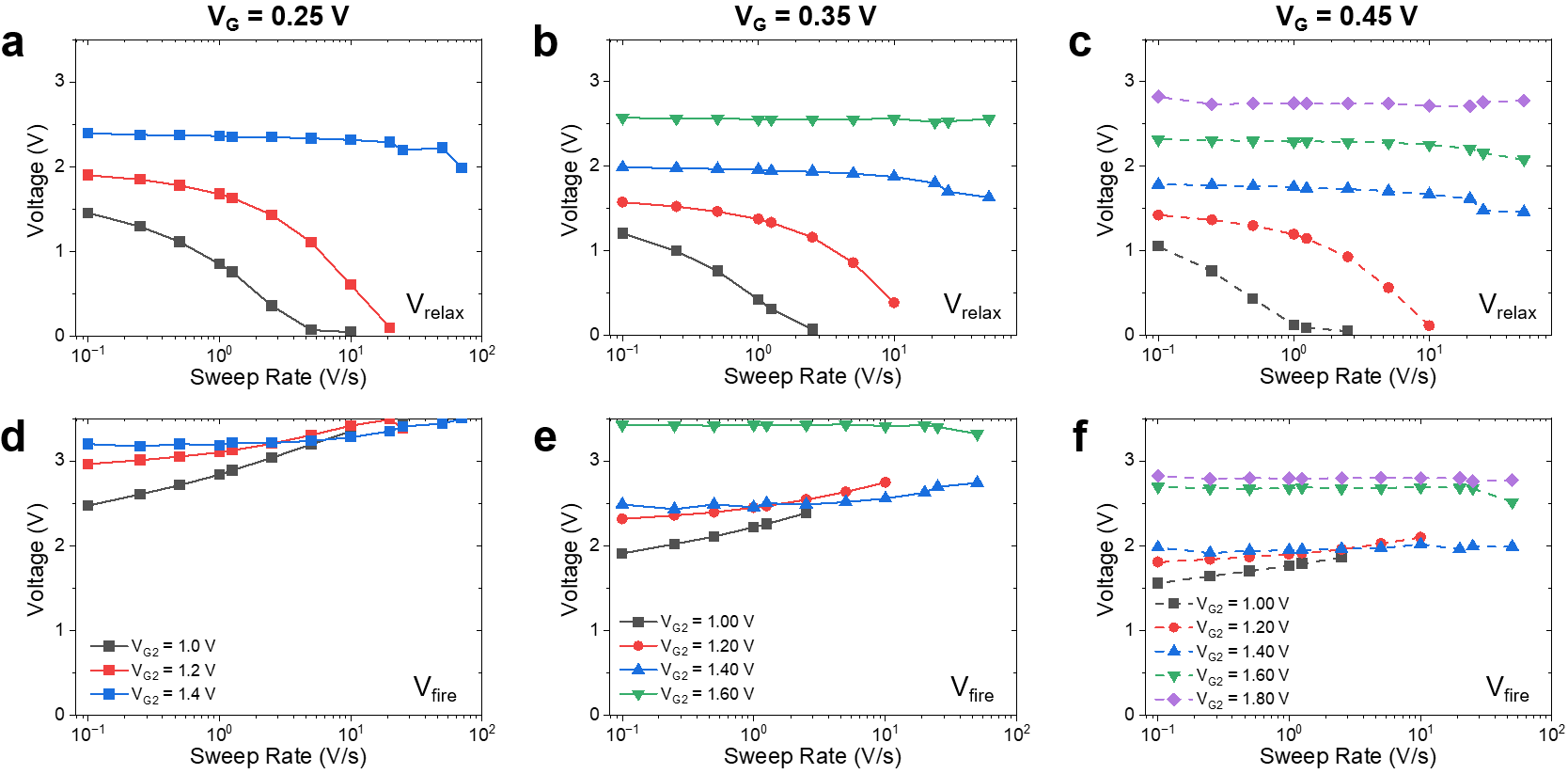


**Supplementary Figure 9 | Sweep rate dependence of firing and relaxation voltages. a, b, c,** Relaxation voltages and **d, e, f,** firing voltages as a function of I-V SMU sweep rate at V_G_ of 0.25 V, 0.35 V and 0.45 V, respectively. Different V_G2_ are represented by distinct colours/symbols. Note a shallower dependence of V_fire_ in all cases (see detailed discussion in Supplementary Note 3).

### **Supplementary Note 3: Physics based simulations of the floating bulk 2-transistor neuron device under experimental conditions**

We perform detailed TCAD simulations of the devices used in this study, replicating the device structure based on the available information of the process and some general assumptions given by the technology node, such as doping profiles which are not precisely known (these are technology proprietary information that is not available). Details of the simulation procedures are provided in the Methods section of the manuscript, alongside a link to a repository containing the full TCAD project for public access, and Supplementary Note 6 provides complete details of the simulation procedure.

To obtain representative results we first calibrate the models and simulations to the experimental data, particularly to the impact ionization currents observed during the measurements of the standard-biased n-channel transistors, i.e., with bulk grounded (see Supplementary Fig. 10 comparing TCAD data with experimental I-V curves).


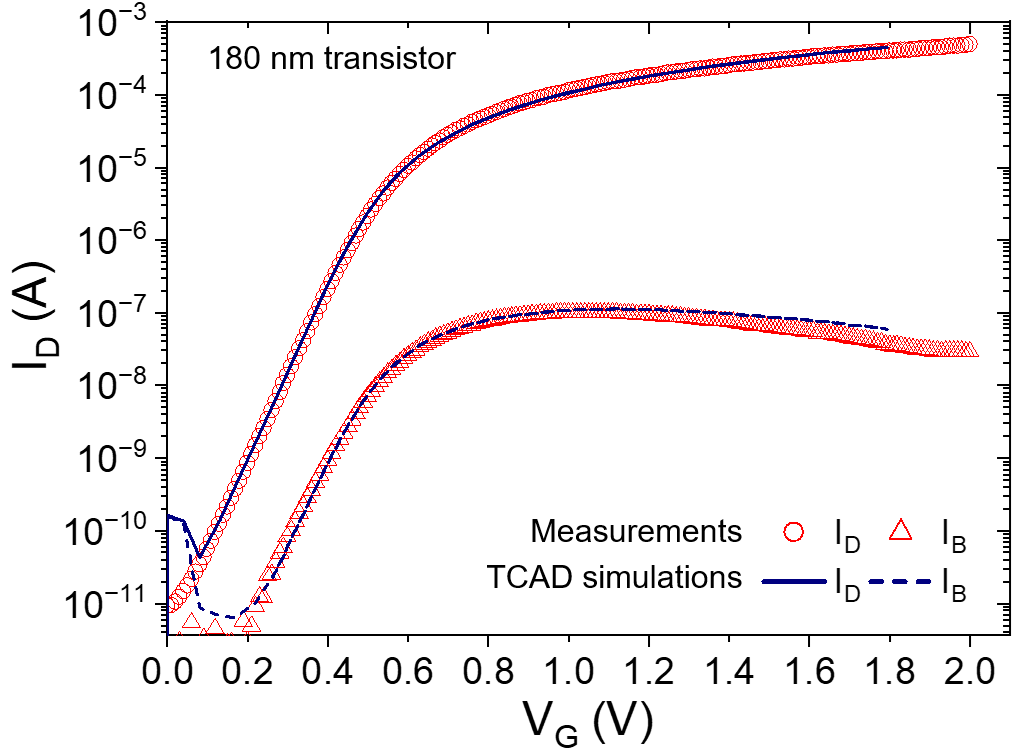


**Supplementary Fig. 10 | TCAD modelling results of the fundamental 180 nm transistor structure.** The simulation models and structure are optimized to replicate the main driving mechanism of the floating bulk device, which is impact ionization (characterized by bulk current I_B_). TCAD simulation results in transient regime (replicating measurement conditions) show excellent agreement with measurement results. In measurements and simulations, V_D_ = 1V.


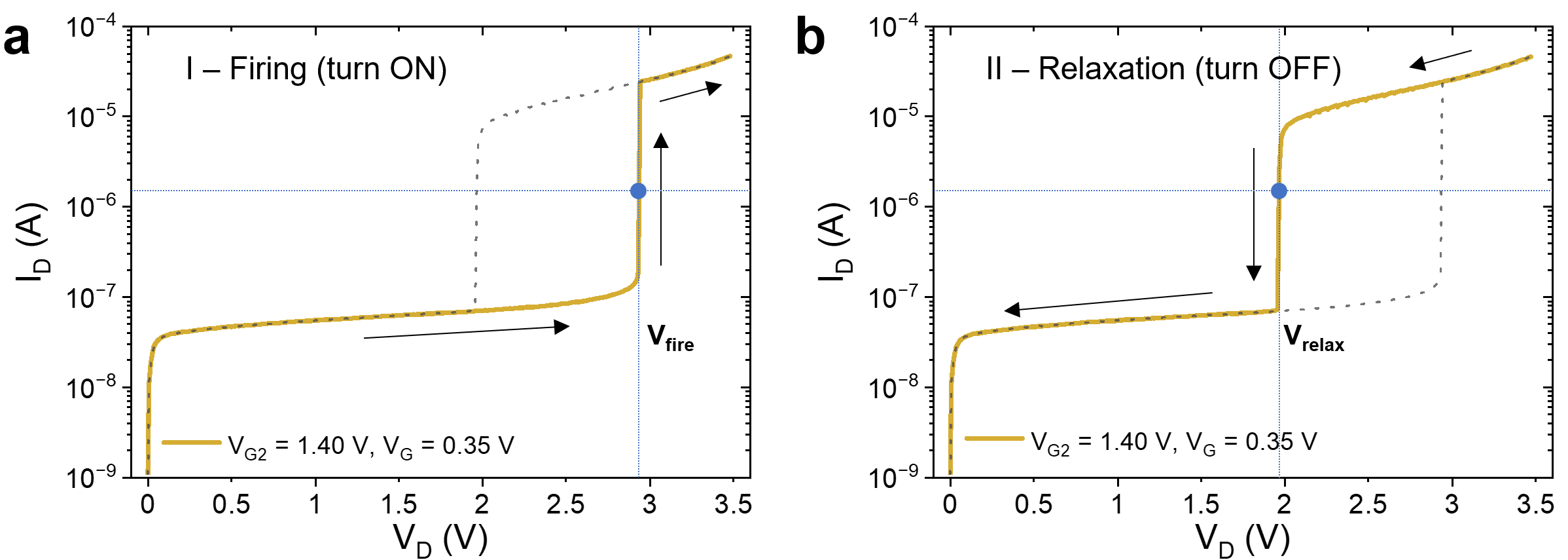


**Supplementary Fig. 11 | ON and OFF transitions of the punch-through impact ionization firing mechanism.** **a**, Turn ON transition (V_fire_) during ramp up of V_D_. **b,** Turn OFF transition (V_relax_) during ramp down of VD. In both cases, the SMU is configured in LIMITED (1 nA) AUTO range mode, which results in an average sweep rate of 0.65 V/s.

To explain the physics in full extent, we split the process in two parts, as depicted in Supplementary Fig. 11: I - the process when the neuron fires (forward sweep in the I-V), and II - the process that takes place when the neuron turns off (backward sweep in the I-V).

**I – Firing (turn ON)**

As drain voltage is increased (ramped up) the build-up of excess carriers related to the impact ionization ^47^ process will result in the progressive firing of the parasitic bipolar transistor in the structure of the MOSFET (see Fig. 2a). The excess electrons get drifted/diffused alongside the channel electrons, while the excess holes can do one out of two things. On one hand, excess holes tend to accumulate in areas with the lowest potential near the channel region. This has been well covered by Moselund et al. ^55^ while exploring punch-through avalanche devices for capacitorless DRAM memory applications and we observe a consistent behaviour: Supplementary Fig. 12 displays the hole density in the structure of the 180 nm MOSFET at equilibrium and fired conditions before and during the firing event.


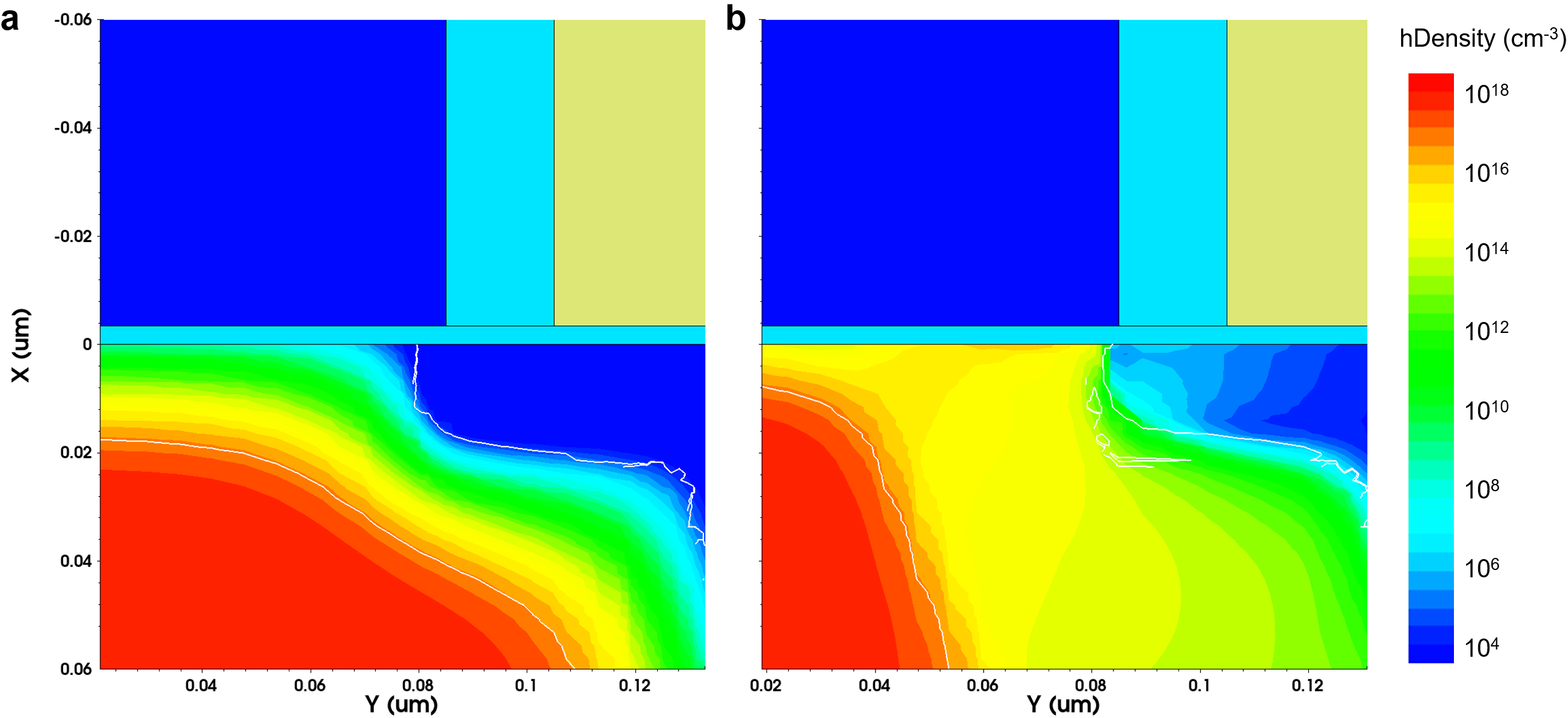


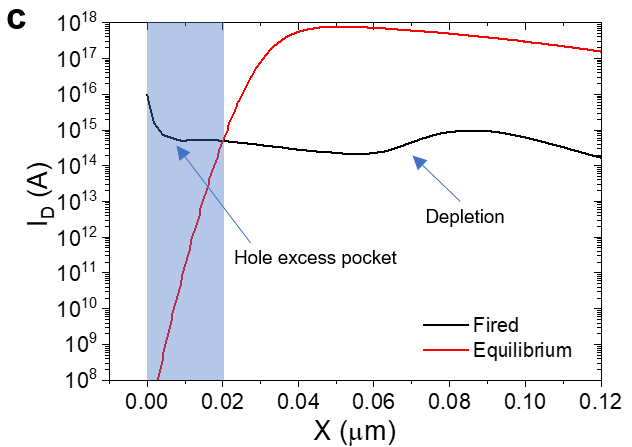


**Supplementary Fig. 12 | Hole density in the floating bulk transistor.** Hole density near the drain region under **a,** equilibrium and **b,** fired conditions. The white lines represent the depletion region. The fired conditions are V_G_ = 0.35 V, V_D_ = 3.5 V, and effective R_B_ = 50 MΩ. **c,** Hole density across the depth of the structure (X) at Y = 0.07 μm, displaying the hole excess pocket near the gate oxide interface, caused by an electrostatic potential well.

On the other hand, holes are also collected through the bulk terminal of the MOSFET, namely I_B_. This collection process can change substantially with the bulk effective resistance, and hence it is directly affected by the bias of the bulk control transistor V_G2._ The raise of I_B_ forward biases the bulk-source junction of the device, which is coincidentally the base-emitter junction of the parasitic bipolar transistor. The fact that the MOSFET is operating near the punch-through condition, means that the effective base of this bipolar transistor is very narrow, which translates to a high DC current gain, defined as the ratio between base current and collector current (β_DC_ = I_C_/I_B_). Therefore, the increase in I_B_ eventually turns on the parasitic bipolar transistor, inducing a high current that adds to the MOSFET channel current, but that can be orders of magnitude higher (specially while operating the MOSFET in the weak/moderate inversion regions, as is the case in our implementation). At this point, the transistor connected to the bulk of the floating body device plays a crucial role on determining the neuron firing voltage: a higher effective resistance of the substrate means a larger voltage drop due to I_B_, hence a higher forward bias of the emitter-base junction of the parasitic bipolar transistor. In other words, the bipolar transistor is “easier” to turn ON, and therefore the voltage value V_D_ at which the neuron fires reduces when V_G2_ is lower. This is clearly depicted by new measurements of the firing voltage dependence on V_G2_, conducted at constant V_G_ and constant ramp rate (see Supplementary Fig. 13a and 13b), and further verified through TCAD simulations where the increase of bulk-connected resistance translates to a lower onset potential of the firing process, which coincides with a collapse of the depletion region at the source-bulk (emitter-base) junction due to avalanche-induced forward bias (see Supplementary Fig. 13c and 13d).


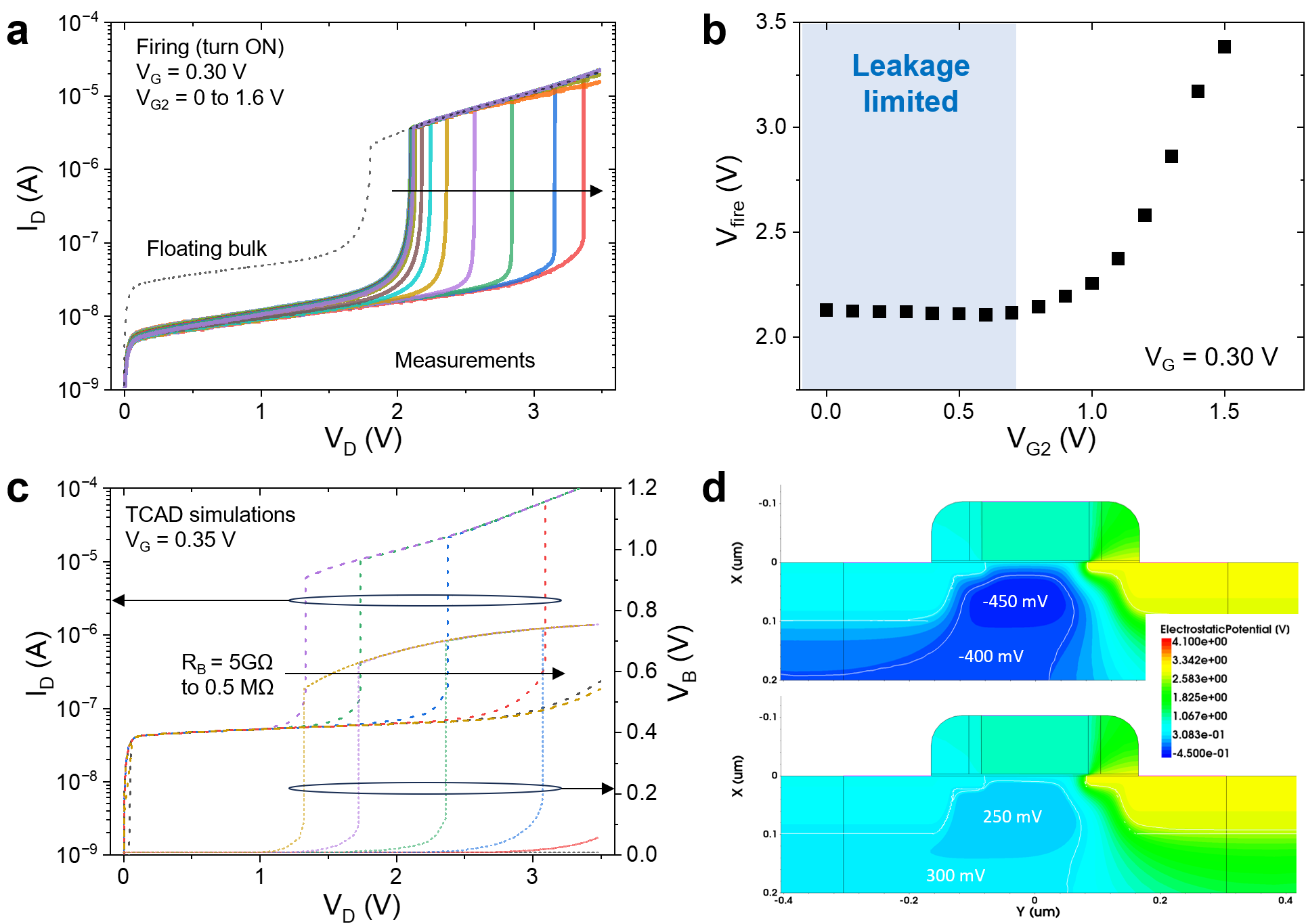


**Supplementary Fig. 13 | ON transition voltage dependence on VG2 (RB).** **a,** Turn ON transitions during ramp up of V_D_ for V_G2_ between 0 V and 1.6 V (dashed curve is for a fully floating bulk condition, no device connected to the bulk terminal). Ramp rate is constant at 0.25 V/s in all cases, and a 10 second reset is applied between sweeps by setting V_G2_ = 5 V with all device terminals grounded. **b,** V_fire_ dependence on bulk control V_G2_. c, Simulations replicating the conditions from panel **a** in a mixed-mode environment, using a fixed resistance R_B_ connected to the bulk terminal of the device structure. Left axis shows I_D_ and right axis shows V_B_, displaying the bipolar transistor firing and showing excellent agreement with measurements. **d,** Electrostatic potential contours right before (top) and after (bottom) the device fires.

Once the parasitic bipolar device has fired, a positive feedback loop takes place which the self-sustains the base current: the raise of the substrate potential through R_B_, which in turn forward-bias the bulk-source (base-emitter) junction, further increases the drain current through bipolar amplification effect, sustaining the impact ionization condition. Commonly, this is an unwanted parasitic effect which can be effectively reduced by multiple device level optimization steps from the fabrication side ^47,50–52^. But it is also well known that a reduced effective bulk resistance naturally diminishes this feedback effect, which is accomplished in VLSI design by the redundant placement of substrate contacts and the careful design of the bias network on chip.

For measurements performed at very low V_G2_ values < 0.8 V, I_B_ is only limited by leakage currents of the experimental setup and the external control device (see Supplementary Fig. 6). This is observed as a saturation in the threshold voltage V_fire_ to ~2.1 V. If the bulk is fully floating, the minimum observed V_fire_ is ~1.8 V.

**II.a – Relaxation at high V_G2_ / low R_B_ (abrupt turn OFF)**

In all cases, when the voltage is swept back down, the current remains high even after the voltage V_D_ drops below the threshold that was required to attain sufficient impact ionization as to fire the self-sustained bipolar device. This gives origin to the clearly observed hysteresis. At this point, the high current is only sustained through the previously mentioned bipolar effect: as long as the voltage drop in R_B_ is high enough to keep the bipolar device in direct active mode (amplification), the channel current will remain high (as the product of βI_B_), mainly driven by this parasitic device.

However, as the drain voltage is driven lower, two effects take place: (i) the depletion region of the drain will tend to get smaller due to the reduction of the reverse bias in the drain-bulk (collector-base) junction, increasing the effective width of the base of the bipolar transistor and reducing its gain β_DC_ (this is denoted by the slope of the I_D_-V_D_ characteristic in the post-fired condition); (ii) the excess carriers provided by the impact ionization process are also reduced, which also represents a decrease of I_B_ and therefore a weaker forward bias of the emitter-base junction in the bipolar device as excess carriers are collected and the electrostatic potential in the bulk of the device is decreased. All in all, when I_B_ falls below the threshold value to keep the bipolar device ON, the parasitic device turns OFF, but this process can have very different dynamics depending on how fast the voltage of the bulk terminal can drop. At high V_G2_ > 1.4 V, the impedance connected to the channel is relatively low in terms of the voltage drop that is generated due to impact ionization currents (100 nA ~ 1 μA I_B_ would generate voltage drops ~ 100 mV). In these conditions, the turn off dynamic is very abrupt (substrate voltage can drop rapidly through a relatively low impedance) and the turn off voltage (V_relax_) shows a flatter dependence on the sweeping rate (see Supplementary Fig, 9a-c. Meanwhile, the turn ON voltage (V_fire_) is slightly more sensitive to the sweep rate (see Supplementary Fig, 9d-f). These differences should be ascribed to the different time dependence of the processes driving the turn ON and turn OFF processes (impact ionization and excess carrier decay/substrate voltage drop, respectively).

**II.b – Relaxation at low V_G2_ / high R_B_ (slow turn OFF)**

When the effective bulk resistance is sufficiently high, the presence of parasitic capacitances at the bulk connection can play a major role on the dynamics of the relaxation process. The total parasitic capacitance at the bulk connection will maintain the high potential of the floating body as it slowly discharges, therefore the forward bias of the emitter-base junction is sustained as V_D_ is swept down, keeping the bipolar transistor ON and showing a less abrupt turn OFF in the backward sweep. Under these conditions, even though excess carriers will tend to recombine (mostly through Shockley-Read-Hall and Auger dynamics) when impact ionization rate is diminished, the capacitance on the bulk acts as a source for excess carriers, maintaining the bulk potential high even as V_D_ is ramped down. This is clearly represented by the measurements displayed in Supplementary Fig. 8: at V_G2_ = 1.4 V, a very shallow dependence of V_relax_ with the sweep rate can be seen, becoming much pronounced at V_G2_ = 1.0 V. In Supplementary Fig. 9 the behaviour of V_fire_ and V_relax_ as a function of sweep rate is displayed for a wider V_G_, V_G2_ space. V_relax_ shows a clear dependence at low V_G2_ < 1.4 V which is much shallower for V_fire_.

This asymmetry allows controlling the plasticity of the neuro-synaptic device or, in other words, how long it takes for the device to relax back to its stationary relaxed state. It is important to highlight that under very high bulk resistance conditions, the relaxation dynamics will be very impacted by parasitic capacitances, which can lead to significant variability when reproducing this effect amongst different measurement setups or circuit configurations. This can be observed through mixed-mode TCAD transient simulations where a lumped capacitance is connected to the bulk terminal of the device. At relatively large capacitances of 10 pF, it is possible to observe this effect and a good agreement with experimental measurements (see Supplementary Fig. 14). For high density applications optimizing speed, the slowest that this process can take place will depend on the parasitic substrate capacitance of the device itself and on the effective resistance of the substrate connection (at constant parasitic capacitance, larger resistance translates to slower relaxation of the floating bulk). In the case of applications that prioritize mimicking of biological processes and their time-domain characteristics (typically slow process with characteristic times > 1 ms), the substrate network can be designed specifically, incurring in area overheads to increase bulk connection resistance and/or capacitance (a typical trade-off when dealing with long time constants).

**
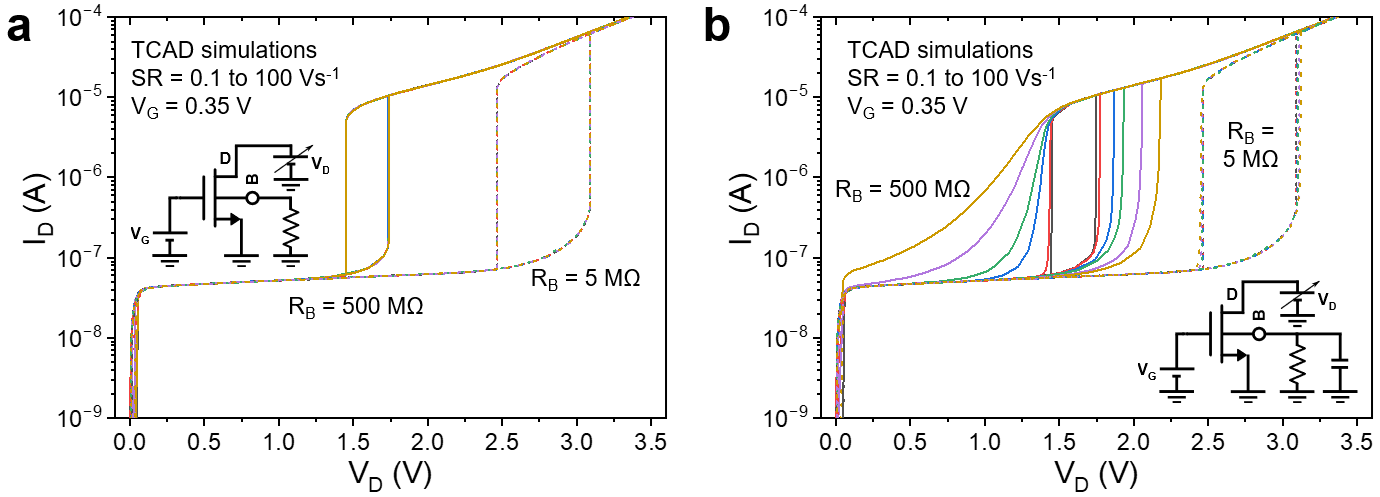
**

**Supplementary Fig. 14 | TCAD transient simulations of sweep rate dependent hysteresis loops.** Mixed mode simulation results for two different configurations: **a,** the device only features an effective bulk resistance and **b,** an additional parasitic capacitance of 10 pF is connected to bulk terminal as well. Note that the dependence on sweep rate vanishes at lower R_B_ for the target sweep rates, but similar characteristics could be observed at higher rates.

**
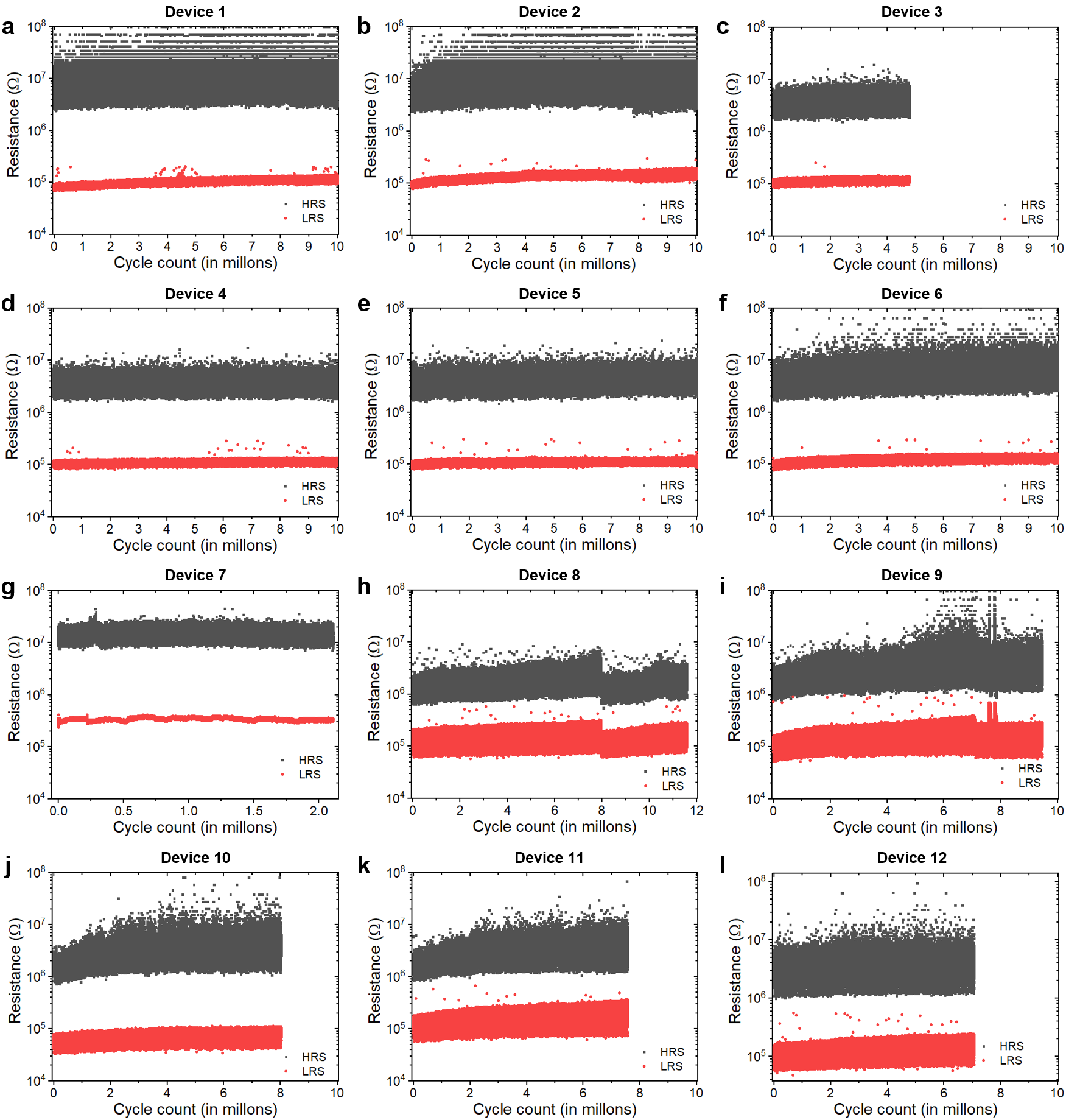
**

**Supplementary Figure 15 | Device-to-device robustness of the firing process under punch-through conditions in 180 nm channel length transistors. a-l,** Cycling under ramped voltage stress (ramp rate between 5500 V/s and 88000 V/s), showing robust behaviour through more than 10 million cycles. For measurements lasting ~8 hours, visible drift is expected due to small changes in contact resistance throughout the long term measurement. Observed cycle-to-cycle variability is acquisition noise of the time domain measurement and not related to the device itself. In all cases, V_G_ is biased between 0.36 V to 0.45 V, peak V_spike_ applied to V_D_ between 4 V and 4.4 V. Devices 1-7 and 8-9 correspond to two different die from Supplementary Fig. 5. Bulk terminals were not connected to an external transistor through the chuck, but instead a constant resistance was connected (~ 1 KΩ) in addition to the substrate spreading resistance of a 5 mm × 5 mm die (estimated 0.1~1 MΩ). Devices 10-12 correspond to same die as 8-9, but connected to a bulk control transistor (as in Supplementary Fig. 7) with V_G2_ = 1.6 V.

**
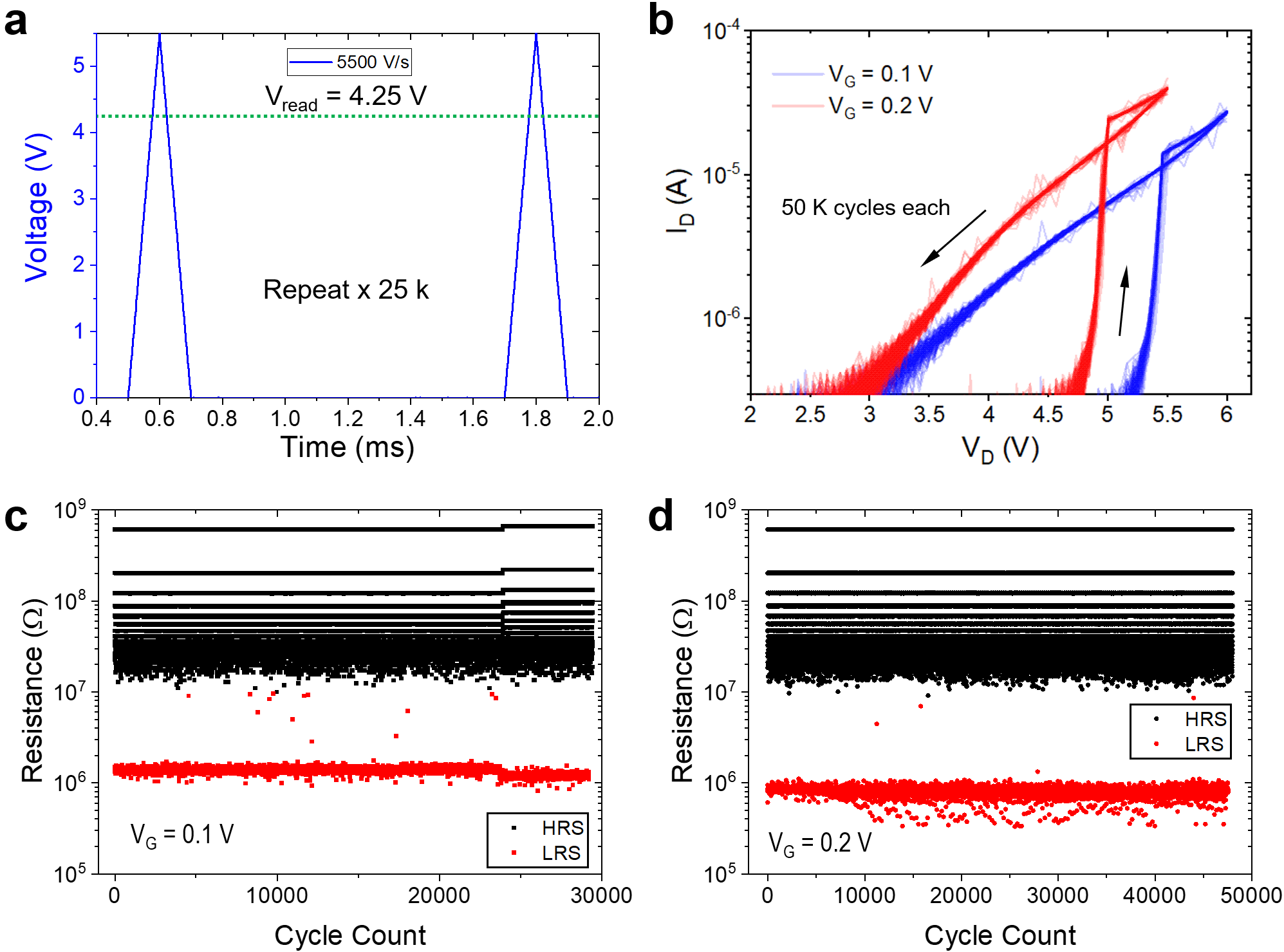
**

**Supplementary Figure 16 | Cycle-to-cycle variability of the firing characteristics under high resolution acquisition for 500 nm channel length transistors. a,** Ramped voltage signal applied for the acquisition, at a ramp rate of 5500 V/s. **b,** Superimposition of 50,000 cycles for two V_G_ conditions on the same device. **c, d,** Resistance extracted at a V_read_ = 4.25 V for every single cycle of the curves shown in panel **b**.

**
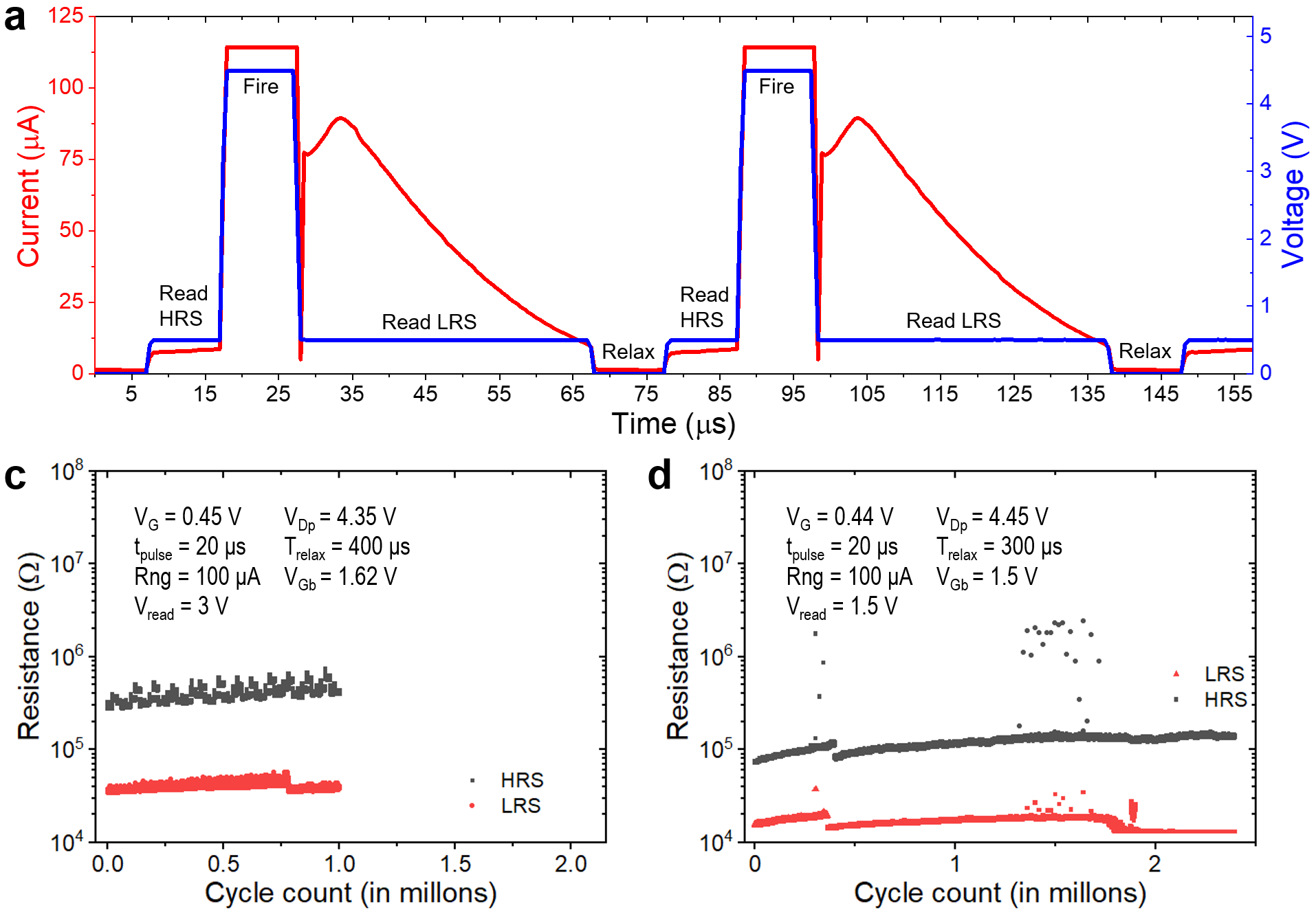
**

**Supplementary Figure 17 | Pulsed regime robustness of the firing mechanism under punch-through conditions in 180 nm channel length transistors. a,** Pulsed waveform to assess the firing and relaxation transient in the µs regime. **b, c,** Resistance (measured at the peak current during the Read LRS section of the waveform) under µs pulse regime. Measurements were stopped after 2 M cycles were performed due to long time required to perform the full acquisition.

**
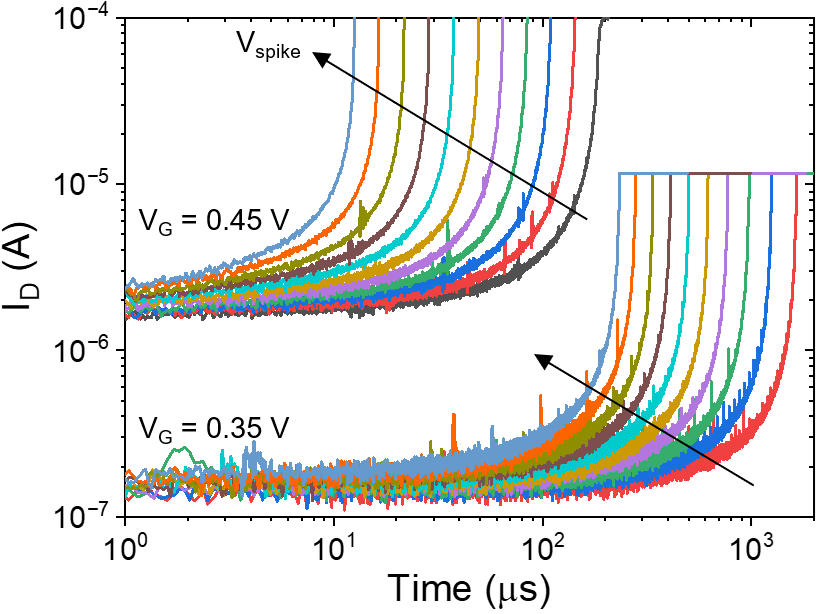
**

**Supplementary Figure 18 | Measurements of the firing time for different voltage spike amplitude in 180 nm channel length transistors.** Impact of gate voltage V_G_ on the firing time and leakage current characteristic at V_G2_ = 1.40 V and different drain voltages (named V_spike_) from 3.60 to 4.50 V.

**
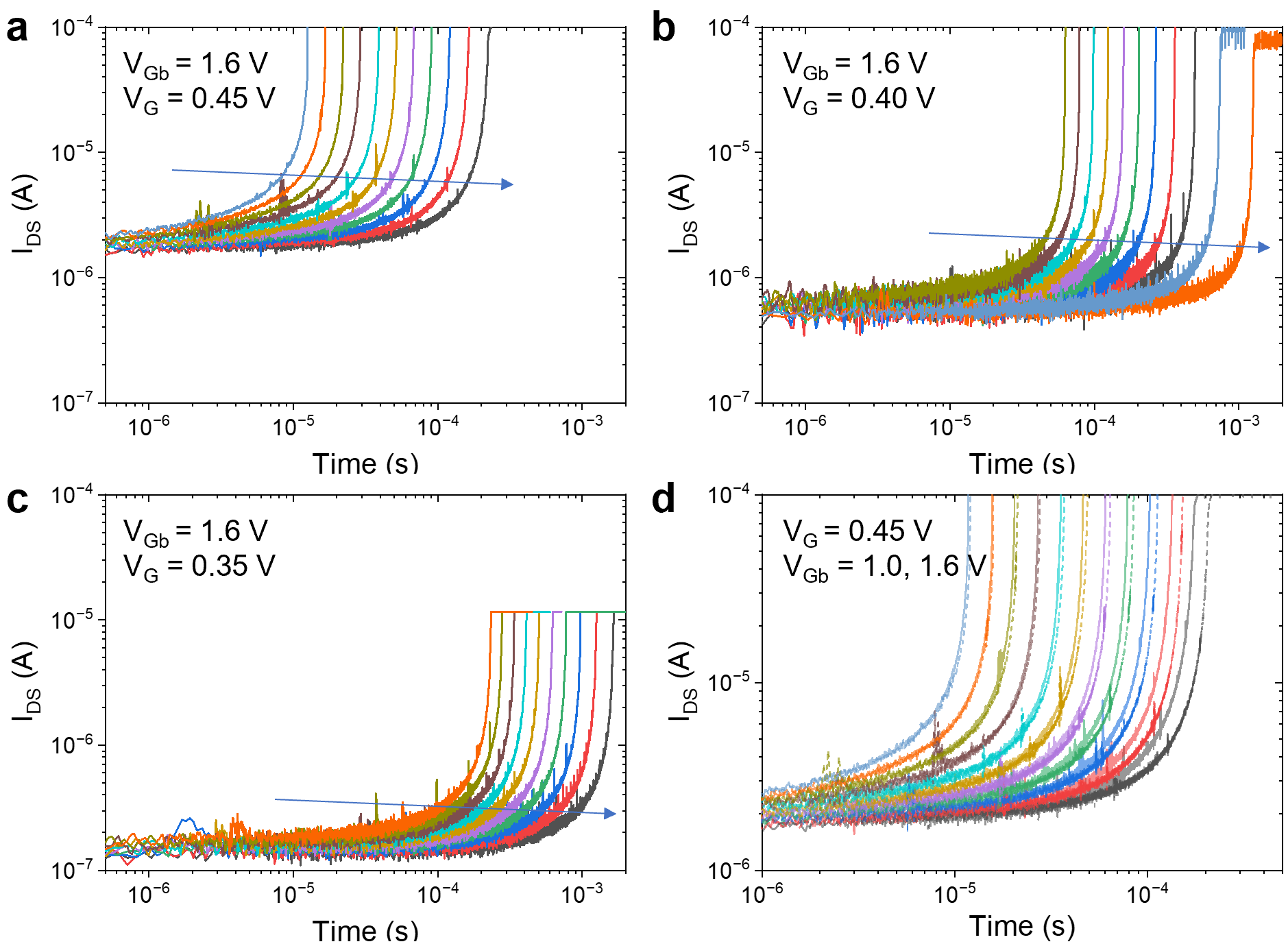
**

**Supplementary Figure 19 | Measurements of the firing time under single voltage spike in 180 nm channel length transistors. a, b, c,** Constant V_spike_ measurements of firing time at V_G_ of 0.45 V, 0.40 V, and 0.35 V, respectively. Each curve is measured at a different V_spike_ between 3.6 V to 4.5 V, at 0.1 V steps. In all cases, V_G2_ = 1.6 V. **d,** Impact of V_G2_ on the firing time. Note a slightly larger firing time at V_G2_ = 1.0 V (dashed curves) than at 1.6 V (full curves).

**
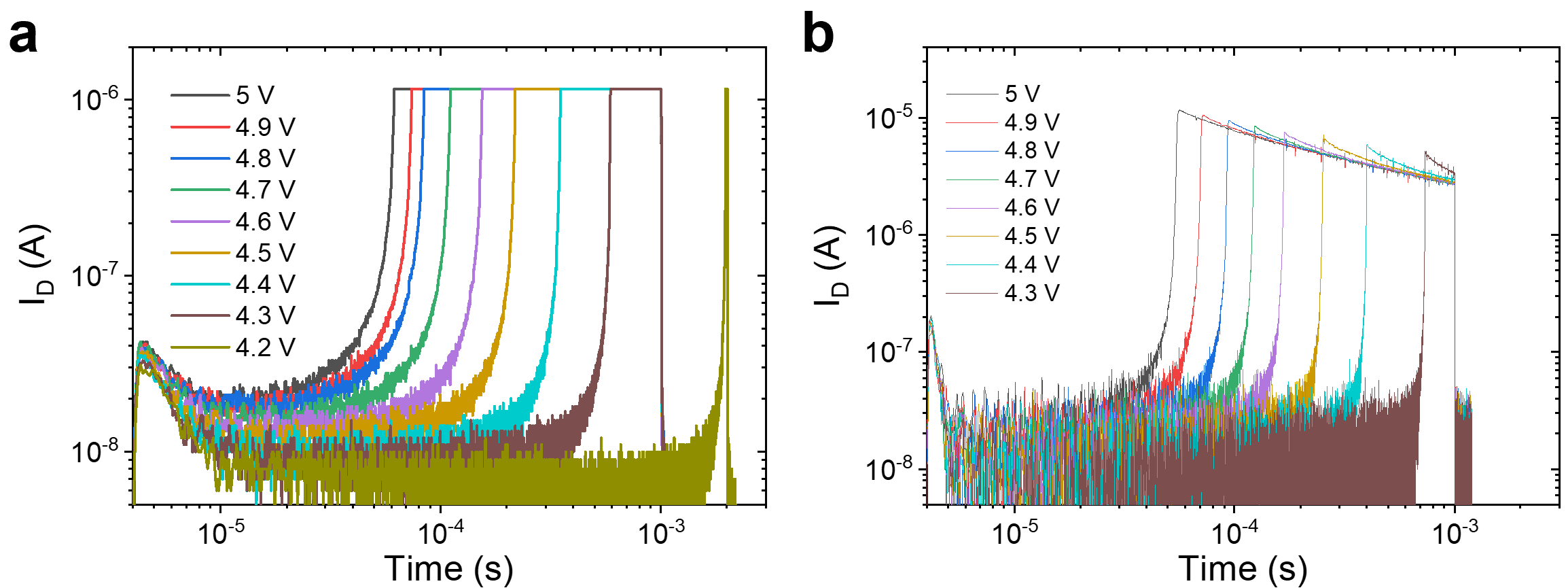
**

**Supplementary Figure 20 | Measurements of the firing time under single voltage spike in 500 nm channel length transistors. a,** Drain current versus time curves at different constant drain voltages (V_spike_) extracted for an amplifier range of 10 µA and **b,** 100 µA. In both cases, V_G2_ = 1.6 V, V_G_ = 0.1 V.

**
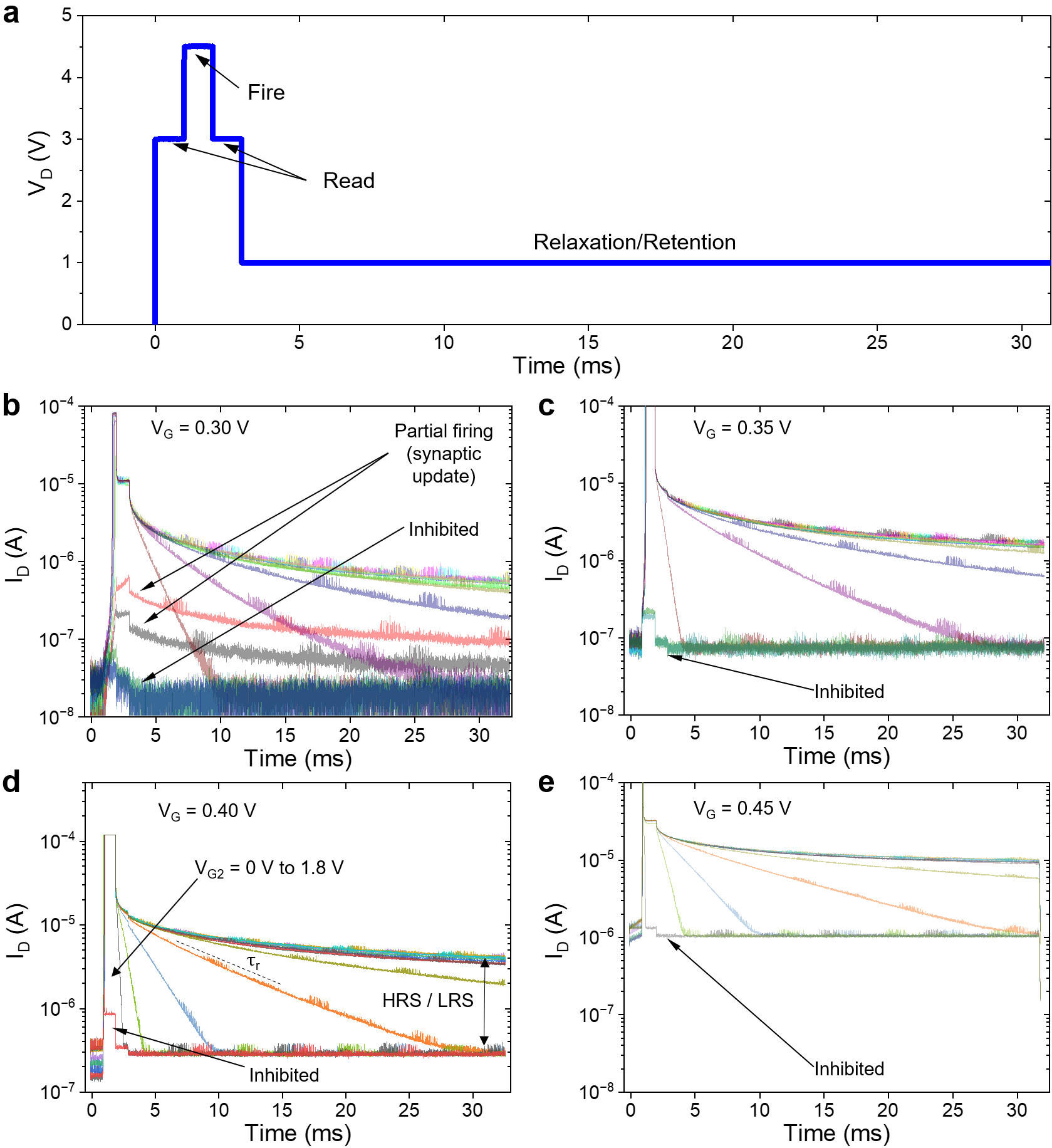
**

**Supplementary Figure 21 | Measurements of the relaxation dynamics of the neural behaviour in 180 nm transistors. a,** Pulsed waveform used to capture the relaxation process of the neural behaviour. The first read voltage (3 V) ensures the device is not fired, while the second read voltage checks the state was changed after the firing pulse (V_spike_ = 4.5 V). The relaxation is captured at a constant voltage of 1 V across a window of 22 ms. **b-e,** Acquisitions of the relaxation transients at V_G_ of 0.30 V, 0.35 V, 0.40 V and 0.45 V, respectively. In all cases, V_spike_ = 4.5 V. Each curve represents a different V_G2_ from 0 V to 1.8 V. For the cases where the device is not fired, this condition is referred to as inhibited neural response. In panel **d**, the HRS/LRS ratio of the synaptic update is indicated by the double arrows.


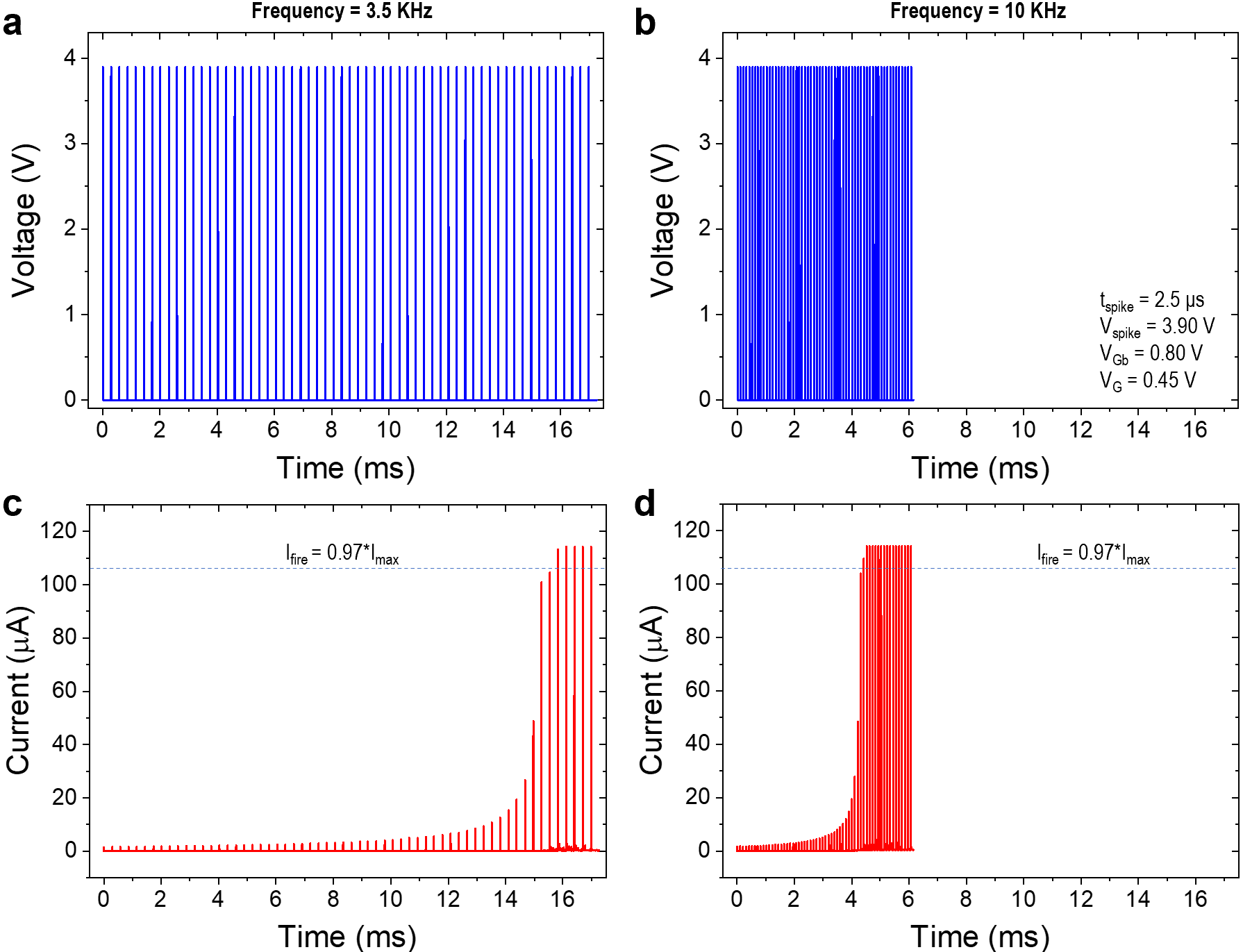


**Supplementary Figure 22 | Spiking frequency dependent firing in 180 nm floating bulk neuron. a-b,** Two examples of pulse trains employed to address firing time at different input spiking frequencies of 3.5 KHz and 10 KHz, respectively. In both cases, t_spike_ = 2.5 µs, V_spike_ = 3.90 V, V_G2_ = 0.80 V and V_G_ = 0.45 V. **c-d,** Current flowing through the neuron device during the pulse train. The firing time is extracted as the instant at which the current level exceeds 0.97 of the maximum current (whether this current is due to acquisition channel saturation of maximum current of the device is reached, depending on the value of V_G_).

**
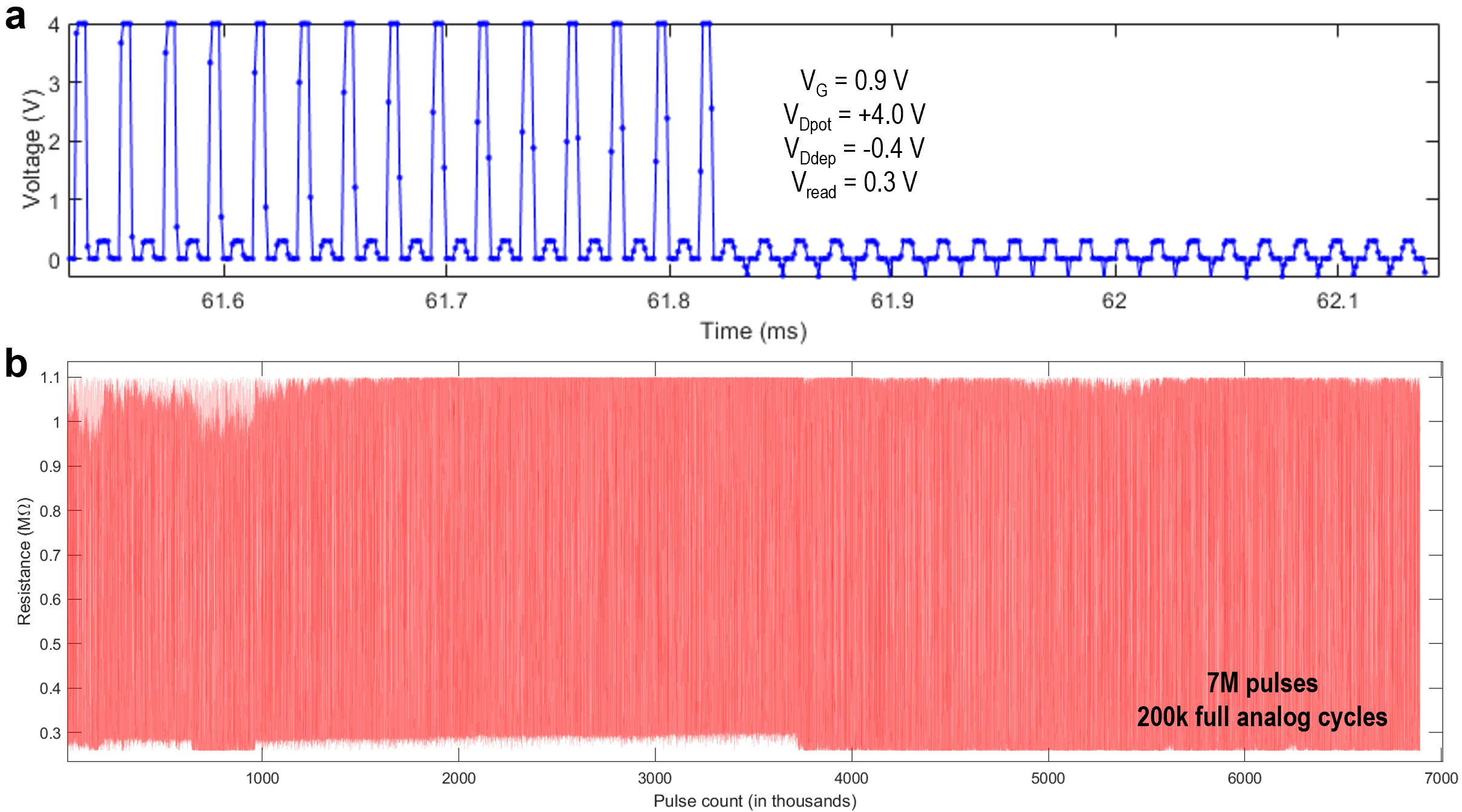
**

**
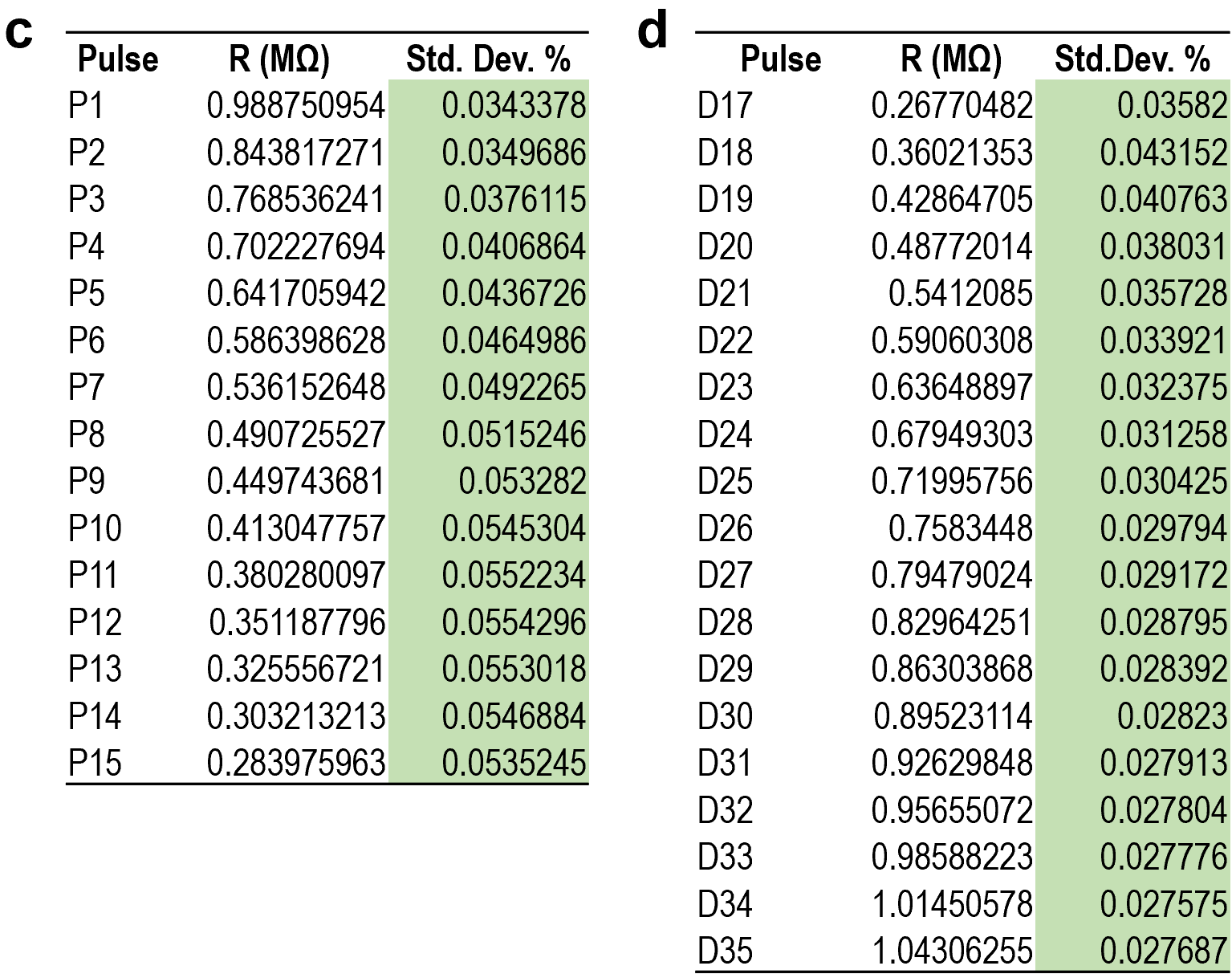
**

**Supplementary Figure 23 | Pulsed synaptic update procedure and statistics. a,** Detailed view of the pulsed voltage waveform for one full potentiation-depression cycle. Read (V_read_ = 0.3 V) and potentiation (V_pot_ = 4.0 V) pulses have a width and spacing of 5 μs, while depression pulses are 1 μs wide (V_dep_ = -0.4 V). **b,** Full view of the extracted resistance (from the read pulses) for a total of 7 million pulses (completing 200,000 full potentiation-depression cycles). Some expected temporal drift is observed throughout the 10-hour measurement due to experimental setup (predominantly tip-to-pad contact stability and substrate noise). **c-d,** Resistance statistics (mean and standard deviation, extracted from the read pulses) after each potentiation and depression pulse across 90,000 full cycles, respectively. Note that long term stability of the tip contact to probing pads induces noticeable changes in the measured resistance in experiments lasting several hours.

**
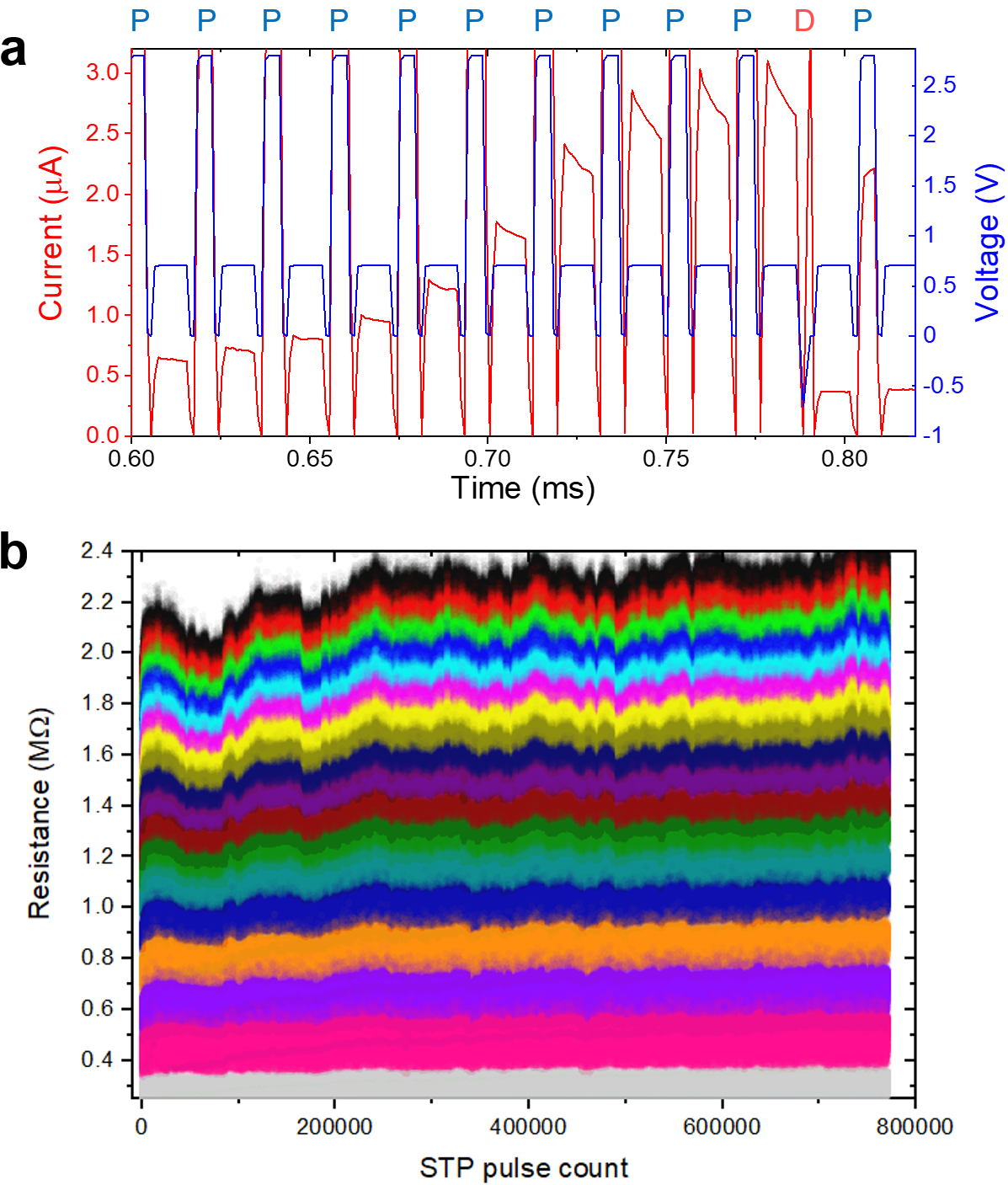
**

**Supplementary Figure 24 | Pulsed synaptic reset and variability. a,** Synaptic update voltage waveforms with single depression (or reset) pulse. All read pulses are 10 μs wide, potentiation pulses are 5 μs wide and reset pulse is 1 μs wide. V_pot_ = 2.80 V, V_read_ = 0.75 V and V_reset_ = -0.70 V. Over the top axis, the “P” stands for potentiation and the “D” for depression, identifying each pulse. **b,** Resistance (extracted from each read pulse) across 760,000 consecutive short-term potentiation cycles. The scatter format shows some overlap due to experimental bench long-term variability and noise, but the individual levels can be clearly identified. Statistics on variability can be found in Fig. 5d of the main text.


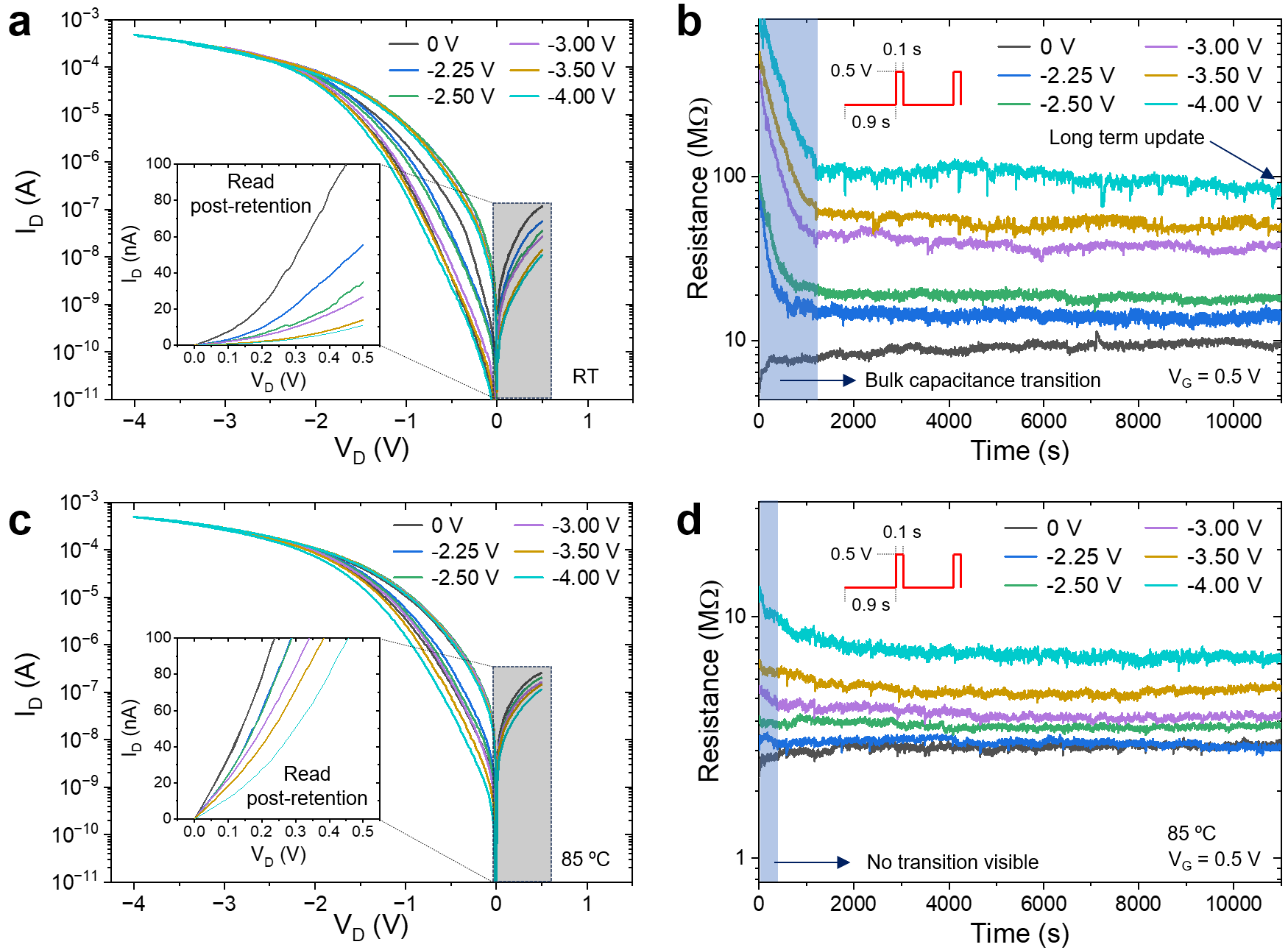


**Supplementary Figure 25 | Retention measurements under long-term periodic read.** **a,** I-V characteristic of reset sweeps (up to different reset voltages ranging from -2.25 V to -4 V) and resulting read I-V sweeps up to 0.5 V (performed after >3 hour retention measurements from panel b). The inset shows the post-retention read I-V sweeps in linear scale. Every reset sweep is preceded by a SET sweep up to 5 V. **b,** Retention results measured after each reset sweep. The read voltage is held at 0 V between read pulses lasting 100 ms, with a period of ~1 s. In both panels, V_G_ = 0.5 V. The long-term retention is preceded by a short-term transition. Each regime can be exploited in different synaptic implementations. **c,** Same measurements as in panel **a** but at 85 ºC. **d,** Same measurements as in panel **b** but at 85 ºC.

**
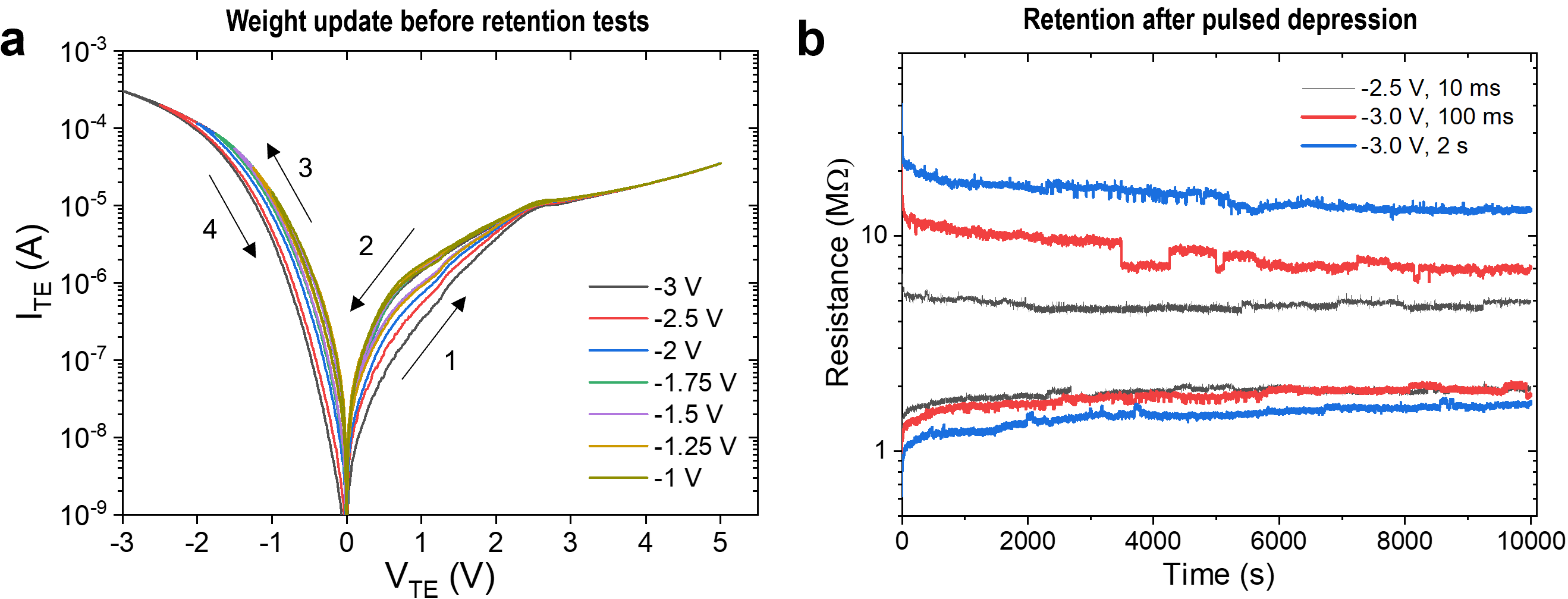
**

**Supplementary Figure 26 | Retention of the synaptic update in 500 nm length transistors. a,** I-V curves employed to assess the effect of the different depression voltages on the synaptic weight and its retention in the floating bulk transistor. A potentiation (positive voltage, curves 1 and 2) sweep up to 5 V precedes every depression (negative voltage, curves 3 and 4) cycle, to guarantee that in all cases the initial synaptic state is the same for all reset voltages. **b,** Retention experiments after pulsed depression with different voltage and duration. Note a clear impact on weight update but similar retention characteristic. Each curve in this plot is preceded by a potentiation cycle as the ones from panel **a** (curves 1 and 2). In all cases, V_G_ = 0.7 V.

### **Supplementary Note 4: physical origin of long-term synaptic update in floating bulk MOSFETs**

Evidence indicates that charge trapping is, most likely, contributing to the long-term retention of the different states in the floating bulk transistor operating in synaptic regime. Mainstream embedded memories typically feature charge trapping mechanisms into floating structures for their operation, but charge injection and trapping can take place without the need of a floating gate. The so-called charge-trapping transistor (CTT) has been widely proposed as a simple alternative to more elaborate embedded memory solutions for their application in storage and in neuromorphic computing applications^75–77^. In these devices, the intrinsically “trappy” gate dielectric of high dielectric constant or high-K (HK) based technologies is exploited as means of synaptic mimicking using standard technology devices. Typically, these devices require high gate and drain voltages (above the nominal) to inject hot-electrons into the HK stack. In the floating body device here studied, voltages beyond the nominal are not required and we find that the trapping behaviour is repeatable within requirements: bear in mind that the 10^5^ cycles of endurance shown in Fig. 4e-f is higher than qualified specifications of some commercial embedded FLASH memories for code storage^78^ featuring 10^4^ cycles, and that charge trapping transistors have been reported for 1,000 cycles in the literature^79^.

From the theoretical aspect, it is most likely that the injection of hot electrons during the reset (negative drain bias) process at a floating bulk condition is contributing to increase the threshold voltage (namely, HRS). Meanwhile, detrapping of some of these injected charge and/or hot hole injection (HHI) can be the mechanism through which the threshold voltage is reduced back to its initial value. In the following, we discuss these two processes (reset and set) individually, for clarity.

In the reset process (increase of threshold voltage or, in other words, increase of resistance under constant bias), a negative bias is applied to the drain at floating bulk condition. Note that, if the transistor bulk was indeed grounded, the current would be determined by the forward bias drain-bulk junction and would rise rapidly, since it would only be limited by the semiconductor spreading resistance and interconnect resistance (see measurements and TCAD simulation results for these conditions in Supplementary Fig. 25a). But with the floating bulk, the decreasing drain voltage tends to forward bias the drain-body junction, lowering the electrostatic potential of the silicon bulk and inducing an inversion channel under the gate (recall that V_G_ is held at constant voltage). Since the source is held at 0 V, it is biased above the electrostatic potential of the body and large currents are driven in the device channel.


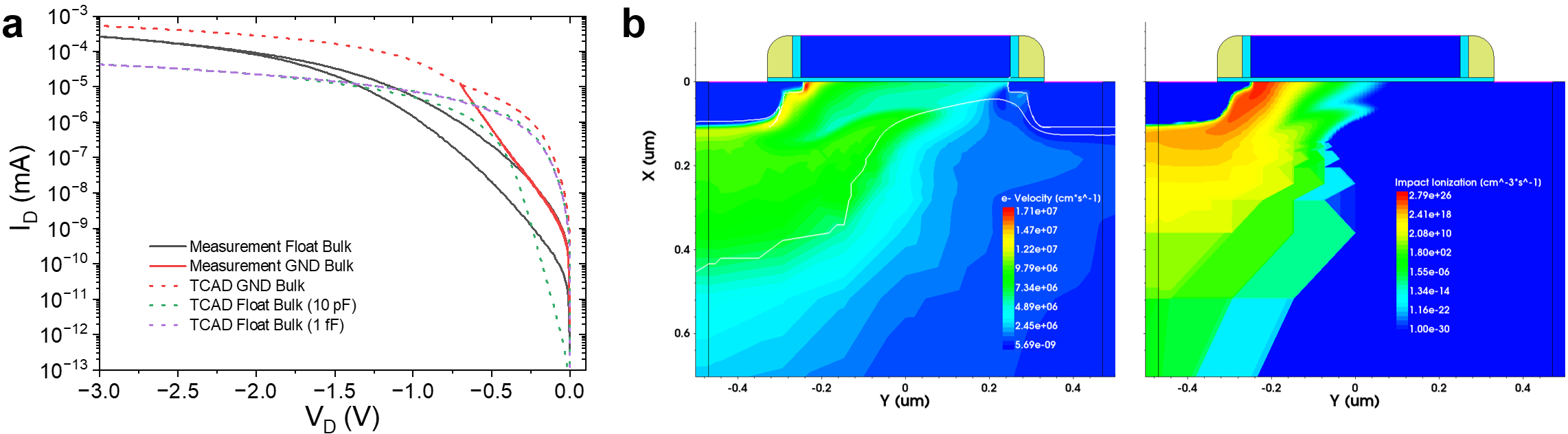


**Supplementary Figure 27 | Device conditions during reset sweeps. a,** Characteristic I_D_-V_D_ reset sweeps (V_D_ < 0 V) of the floating bulk transistor operating in synaptic regime and the same device with the grounded bulk (forward drain-bulk junction bias). Full curves are measurements and dashed curves are TCAD simulation results (differences are expected due to lack of detailed information on the parameters of the front-end-of-line). Note that, the current of the floating bulk device is much smaller. Floating bulk simulations were carried out using a large resistance connected to the bulk (10^13^ Ω) with a capacitor in parallel (1 fF or 10 pA). Larger capacitance at the bulk terminal explains the observed hysteresis. **b,** Electron velocity (left) and impact ionization rate (right) at the floating bulk condition when applying -3 V at the drain terminal. Note presence of hot electrons near the source, at the oxide-silicon interface.

It is important to highlight that the observed hysteresis during dual sweeps is related to the parasitic bulk capacitance in the device and interconnects (see difference in simulations with -green dotted- and without -lilac dotted- external additional capacitance), which again can maintain the substrate potential low until it discharges through leakage sources. However, this process eventually relaxes as the total bulk capacitance discharges. This is most likely the origin of the relaxation observed in retention measurements (Supplementary Fig. 23).

When the drain voltage is sufficiently negative, energetic electrons are present in the vicinity of the source terminal (see impact ionization rates from the TCAD calculations in Supplementary Fig. 25b) and are likely to be injected into the gate oxide. This will logically result in an increase of V_th_ and therefore a reduction of current drive capability. This tends to shift the V_th_ to a higher voltage, therefore lowering the drive current capability of the transistor, which is translated to a high resistivity state under read conditions.

During the set process, some detrapping of the charge generated during the reset sweep can be expected, but the damage by hot electrons is typically non-reversible^80^ (at least without annealing conditions). Therefore, it is likely that the injection of holes through the gate dielectric on the drain side may also take place at impact ionization conditions. This is a well-known process that has been observed in standard silicon transistors since 1986 (Ref. ^81^). This phenomenon takes place at high drain voltages, where band-to-band tunnelling is likely in the drain-bulk junction and highly energetic holes and electrons are present at impact ionization conditions. Since excess holes are not collected by the bulk current in the floating bulk condition and the density of holes tends to increase at the oxide interface close to the drain (as discussed previously in Supplementary Fig. 12), conditions are given for hole injection. Just like with electrons, hot holes can be injected through the gate dielectric if they have enough energy to overcome the energy barrier. As a result, this effect has been observed to be responsible for read disturb instability in EEPROM memories^82,83^ and is employed as an erase mechanism in some commercial embedded flash memories^78^, as injected trapped holes result in a decrease of the threshold voltage. In such cases, the hot holes that are injected can be effectively concealed within the floating gate structure, but in standard MOSFET structures a lesser number of holes can be trapped in defect centres of the gate-oxide and/or spacer oxide of the MOSFET structure.

We have also performed retention measurements to address the permanent charge trapping behaviour without accounting for any floating bulk effects during the retention reading period. This is done by performing set and reset sweeps (see Supplementary Fig. 26a) under a floating bulk condition and then running the retention experiment with the bulk of the device grounded (see Supplementary Fig. 26b), followed by I_D_-V_G_ curves to address the V_th_ shift (ΔV_th_) after each measurement (see Supplementary Fig. 26c). These results show that a long-term synaptic effect is possible without the need for a floating gate structure by exploiting intrinsic charge trapping in standard silicon transistors aided by the floating bulk configuration. Note that different V_G_ voltages upon read can also enable a trade-off between readout sensitivity, power consumption and speed.

All in all, the floating bulk condition allows to use a single, standard, silicon transistor in a similar fashion as charge trapping transistors based on high-k oxides. **It is very important to highlight that in this regime the nominal operating voltage of the device (5.5 V) is never exceeded, so the displayed memory effect is not related to a high voltage regime but to the bipolar operation of the device in the floating body configuration. More importantly, the floating body enables the possibility to change between hot-electron injection and hot-hole injection without high forward bias currents (see Supplementary Fig. 25a).** This enables very relevant and commercialized technologies for mixed-signal circuits (above 24 nm, without high-k dielectrics) or even enhance the trapping processes with high-k/metal gate stacks to include high density memory/synaptic devices without the need of additional process steps.


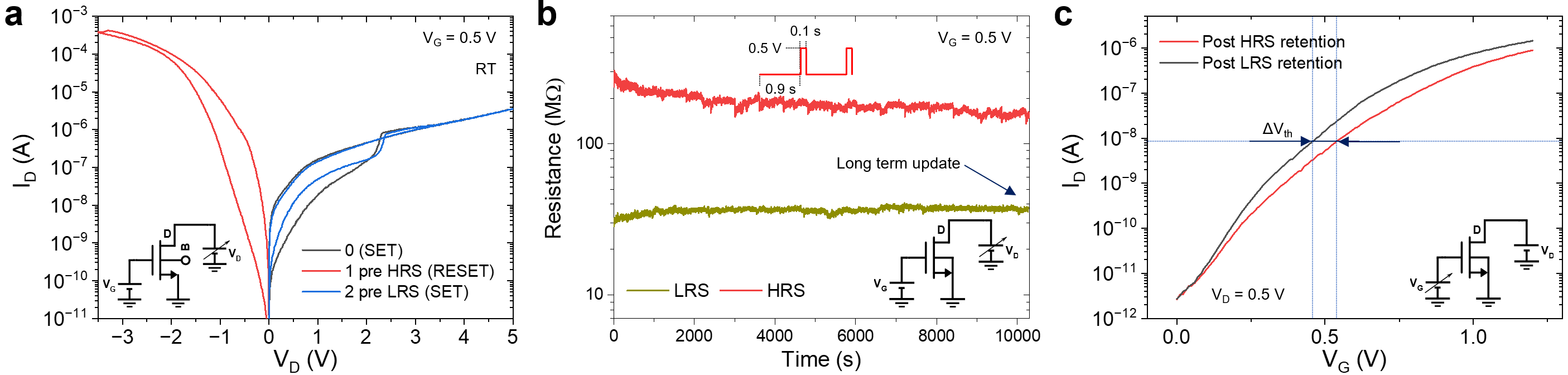


**Supplementary Figure 28 | Retention measurements with grounded bulk of the synaptic device after set/reset in floating bulk condition. a,** Characteristic I_D_-V_D_ sweeps denoting the floating bulk transistor operation in synaptic regime. The body of the transistor is left floating (single device cell). **b,** After the set (red) and reset (yellow) sweeps, retention measurements are carried out with the bulk of the device grounded and under a periodic read regime (see insets). **c,** I_D_-V_G_ characteristics of the device after each retention measurement from panel **b**. The shift of the curve denotes charge trapping and the impact of interface states. Note that in the 500 nm device, nominal operating voltages are never exceeded, indicating that the trapping process is not a result of high operating voltages but of the operation under floating bulk condition.

### **Supplementary Note 5: Reliability aspects related to drain voltages under pulsed operation**

From DC and pulsed characterization, the operating voltages to observe impact-ionization firing for neuron mimicking can be slightly above the nominal DC operating voltages for the working technology (in the case of 180 nm transistors). This becomes clearer when operating under high-speed ramp or pulse conditions, where peak voltages can reach ~4 V. Therefore, reliability is an important aspect to be addressed whenever a device is proposed to operate within specific conditions that may fall outside the nominal. Although a detailed reliability qualification procedure is required to address all working conditions for a specific device in a certain application, for the floating bulk transistors we highlight that reliable operation is possible, supported on multiple aspects:

1. **Operating principle can take place within nominal voltages.** In the experiments carried out in 500 nm devices, the applied voltages in our experiments never exceed nominal operating voltages (5.5 V nominal from the manufacturer) and the punch-through impact ionization process that fires the device takes place at voltages well below the nominal in the floating bulk condition (down to ~2.5 V). This indicates that different front-end-of-line design can be employed to facilitate this regime in case a lower operating voltage was desired.
2. **Tuneability allows to operate within maximum voltages.** In the 180 nm MOSFET, the neuro-synaptic regime can be achieved at lower voltages, at the cost of higher power consumption (higher currents), by modulating the neuron response through gate bias voltage V_G_ and substrate resistance control (V_G2_). Under certain conditions, the firing voltage can be as low as 2 V, which is within the corner specification of the nominal voltage (1.1×V_DD_). From the purely experimental perspective, during the experiments carried out for this work we observed only 3 events of dielectric breakdown within more than 100 devices (180 nm channel) that were intensively measured. We cannot ascribe these events to the specific voltages that were used. In fact, we have measured multiple devices under sweeping, pulsing and (newly added) bursting conditions for millions of cycles and did not notice a degradation on the behaviour of such devices. This doesn’t mean to imply that these voltages would pass a very stringent, industrial reliability requirement. But, while this aspect is of utmost importance, it requires a specific qualification procedure that should be addressed in a technology/process-specific manner.
3. **High drain voltage by itself may not represent a risk on reliability.** Stating that the voltage is higher than the nominal is not enough to indicate a definite reliability limitation. Specific circuit configurations can drive drain voltage over the nominal in the off condition for transient periods of time (e.g., class AB/C amplifiers, see Ref.^84^), and careful assessment of the reliability can show that this condition is acceptable for certain applications. For the working conditions of the devices under test, only the drain voltage is pushed to voltages higher than the nominal, while the channel remains between the OFF and the weak inversion conditions, well below the threshold voltage. As the reviewer has well pointed out, this puts the device under high V_DG_ stress, which is at most ~1.8× the nominal voltage (1.5 V for this technology by this manufacturer). However, as mentioned previously, this operating voltage can be reduced to 1.1× the nominal voltage and it is well known that, under OFF condition stress, the dielectric breakdown in MOSFETs takes place on the spacer side, with a long percolation path and a resulting higher voltage tolerance. Note that, for transistors of the 130nm node, accelerated time dependent dielectric breakdown experiments at room temperature are conducted at voltages as high as 16 V (namely, ~10× higher than the nominal voltage)^85^. Therefore, a detailed reliability study should contemplate the full range of operating conditions of the device, which may limit the full operational voltage space here described to ensure reliability.
4. **Device front-end-of-line can be optimized for operating in this specific regime at lower voltages.** The parasitic mechanism of punch-through impact ionization being employed is an unwanted effect in standard CMOS circuits, and therefore CMOS devices themselves are designed to minimize the presence of this effect under nominal conditions, as was discussed in the original version of the manuscript. Even under these conditions, the avalanche behaviour is well-known and used in VLSI circuits as electrostatic discharge (ESD) protection devices on input/output pins, as discussed in revised Supplementary Note 2. Therefore, it is expected that higher voltages than nominal are required to exploit this effect on standard CMOS devices but is still featured in non-core devices as a standard phenomenon. Even in these conditions, for the sake of exploiting this phenomenon for this specific application, device engineering is definitely possible within standard procedures in commercial foundries, as evidenced by the wide availability of speciality devices (commercial examples include processes by XFAB, Tower Semiconductors, TSMC, among others) in current CMOS technologies that are very mature and are still widely commercialized nowadays (14 nm and above). Therefore, even if some degree of redesign and optimization of the devices is required to evacuate any possible reliability concerns, this does not diminish the potential of the proposed approach. Moreover, this device optimization would be done on a vastly well-known platform, with decades of experience on the making and with thousands of specialty devices that have been tailored to perform specific tasks in analogue/mixed-signal integrated circuits, and for this reason we see this as extremely feasible for the dominating silicon processing industry.

### **Supplementary Note 6: Tuneable frequency bursting neuron and its application in neural networks**

The floating bulk two-transistor neuron can produce a configurable integrate and fire neuron behaviour. Integrate and fire functions fundamentally consist of accumulating potential from multiple temporal inputs for a finite time and, when the accumulated potential exceeds a threshold, they produce a temporal output. While the integrative aspect is provided by the generation and recombination rate of excess carriers in the verge of punch-through impact ionization conditions, the firing dynamics are provided by the parasitic bipolar transistor in the MOSFET structure, that can be finely tuned via a bulk connected control device and gate voltage control. This already poses an important advantage compared to other emerging devices used for this end, such as diffusive memristors: while in memristors the response time is pretty much fixed by the materials involved (electrodes and switching medium) with a certain dependence on the input magnitude, the 2-transistor neuron allows for a wide range of response times within a single, simple device.

However, the integrate and fire neuron is a first order behaviour of a neuron in its simplest terms. Individual biological neurons are well known to be capable of higher order functions in specific roles. Particularly, neurons that play fundamental roles in the production of motor, sensory, and cognitive behaviours produce periodic action potentials, or “bursts” at the output once a certain input threshold excitation is reached. In its simplest form, neurons can produce periodic spikes in response to a constant input. If this period (frequency) depends on the input magnitude of the excitatory signal, the process is known as spike number adaptation^1^.

Some memristors have shown certain bursting capabilities determined, once again, by the materials involved in its structure^1^. A typical approach is to use simple thresholding memristors within a relaxation oscillator, requiring a couple extra components. Approaches involving CMOS devices include similar neuron implementations that have been recently demonstrated using partially depleted silicon-on-insulator MOSFETs^27,28^. However, these devices operate in a band-to-band tunnelling regime, which requires negative gate bias, and also require multiple additional circuits to generate a reset signal through the bulk control transistor in order to operate in bursting mode. Moreover, the spiking frequency can be tuned in the range 1 KHz – 1 MHz, which is much higher than the frequencies of biological processes, typically a drawback in multiple bio-mimicking approaches that require large area and power overheads to reach biological regimes^26^.


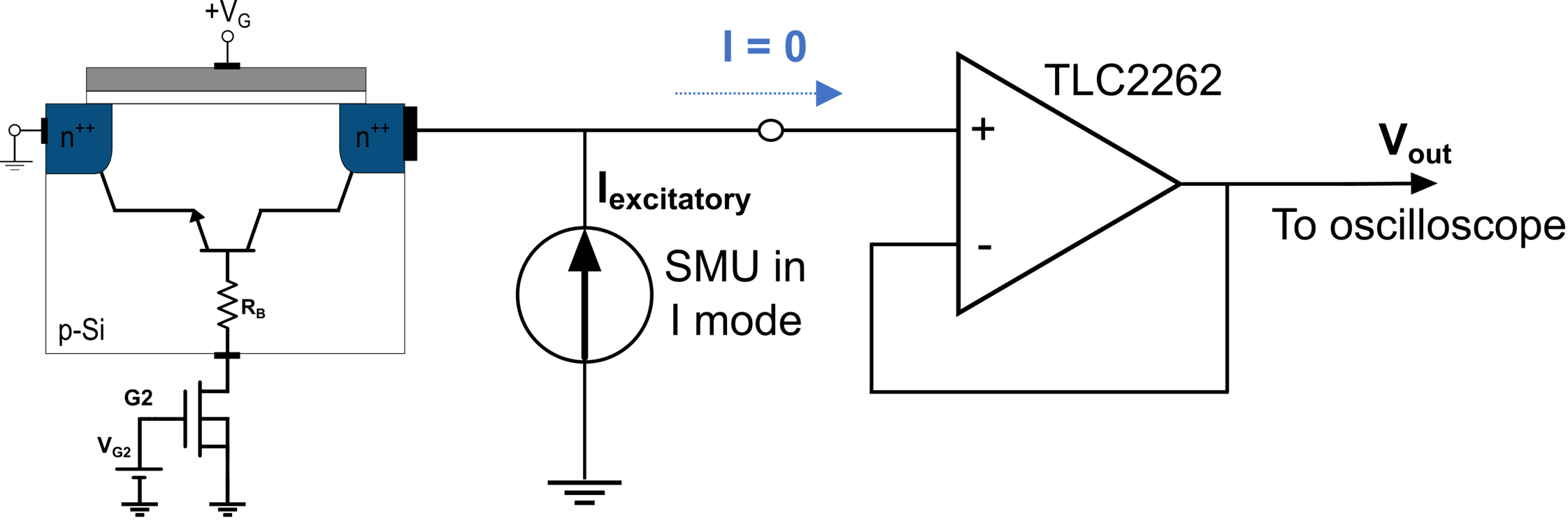


**Supplementary Fig. 29 |** **Experimental testbench for floating bulk transistor in tuneable bursting neuron mode.** The floating bulk transistor is connected in the two-device configuration to a current source, a voltage amplifier and an oscilloscope.

The experiment testbench (see Supplementary Fig. 29) is run by contacting an individual 180 nm transistor with the probe station and configured in the two-transistor device using an external off-the-shelf transistor (BS170). The drain of the floating bulk device is connected, via probe tips, to a current source (SMU in current source mode) and to a high-input impedance voltage follower built with off-the-shelf components (TLC2262). The objective is to minimize the load on the output of the floating bulk device. The output of the amplifier drives the input impedance of a passive compensated oscilloscope probe connected to a digital sampling oscilloscope (MSO-X 3024G). All the instruments are controlled by a single MATLAB script that forces a constant excitatory current through the SMU and immediately launches an oscilloscope acquisition. The excitatory current is swept in steps and the firing frequency of the neuron is measured in the oscilloscope and in the MATLAB script based on the captured waveform. V_G_ and V_G2_ are also parametrically swept to cover the whole range of tuneable frequencies of the floating bulk device. Typical neuron firing results for different biasing conditions are displayed in Supplementary Fig. 30, and the tuning range of the firing frequency is displayed in Supplementary Fig. 31a as a function of input excitatory current. By considering different biasing points for V_G_ and V_G2_, the configurable neuron can burst in the range of frequencies 1 Hz ~ 2 KHz, well in agreement with the characteristic firing times reported in Fig. 3. These frequency values are an excellent fit to mimic biological processes of neuro-receptors, such as retinal or cochlear sensory cells^26^, and the bursting behaviour of neurons observed from cells in motor and positioning centres of the brain^86^, displaying the versatility of the 2-transistor neuron. This is achieved for input excitatory currents between 1 nA/μm ~ 20 μA/μm (total current is proportional to transistor width), with a roughly linear dependence of frequency on input bias. State-of-the-art polymeric transistors that are well fitted for this purpose still require large circuits with large passives (capacitors and resistors) to mimic neuron firing a these bio-inspired frequencies (see Supplementary Fig. 31b)^8^.


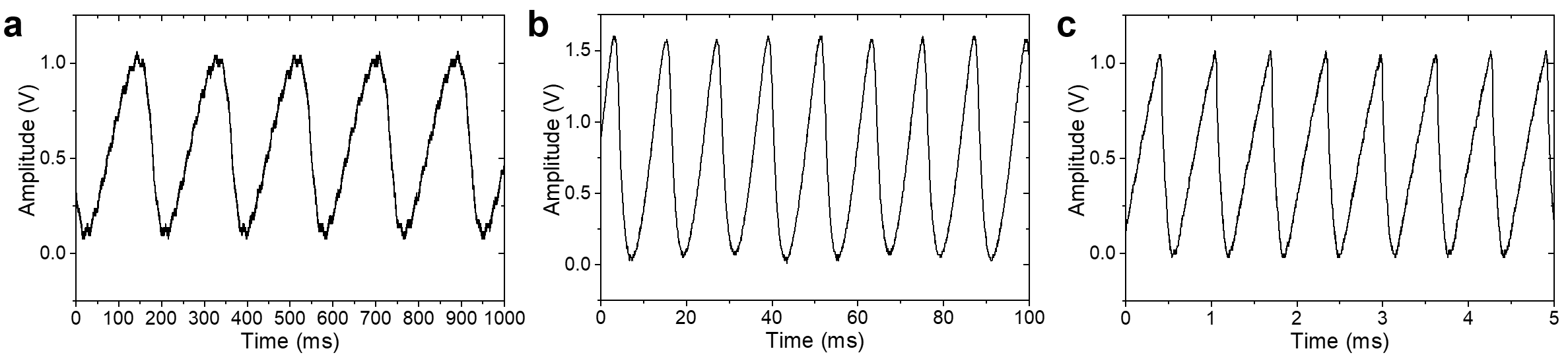


**Supplementary Fig. 30 | Oscillatory spiking behaviour of tuneable floating bulk neuron.** Using V_G_, V_G2_, and the value of the excitatory current, the floating bulk device can show configurable spiking across a wide range of frequencies. Note that low frequencies (panel a) are damped by parasitic capacitance effects of the characterization setup.


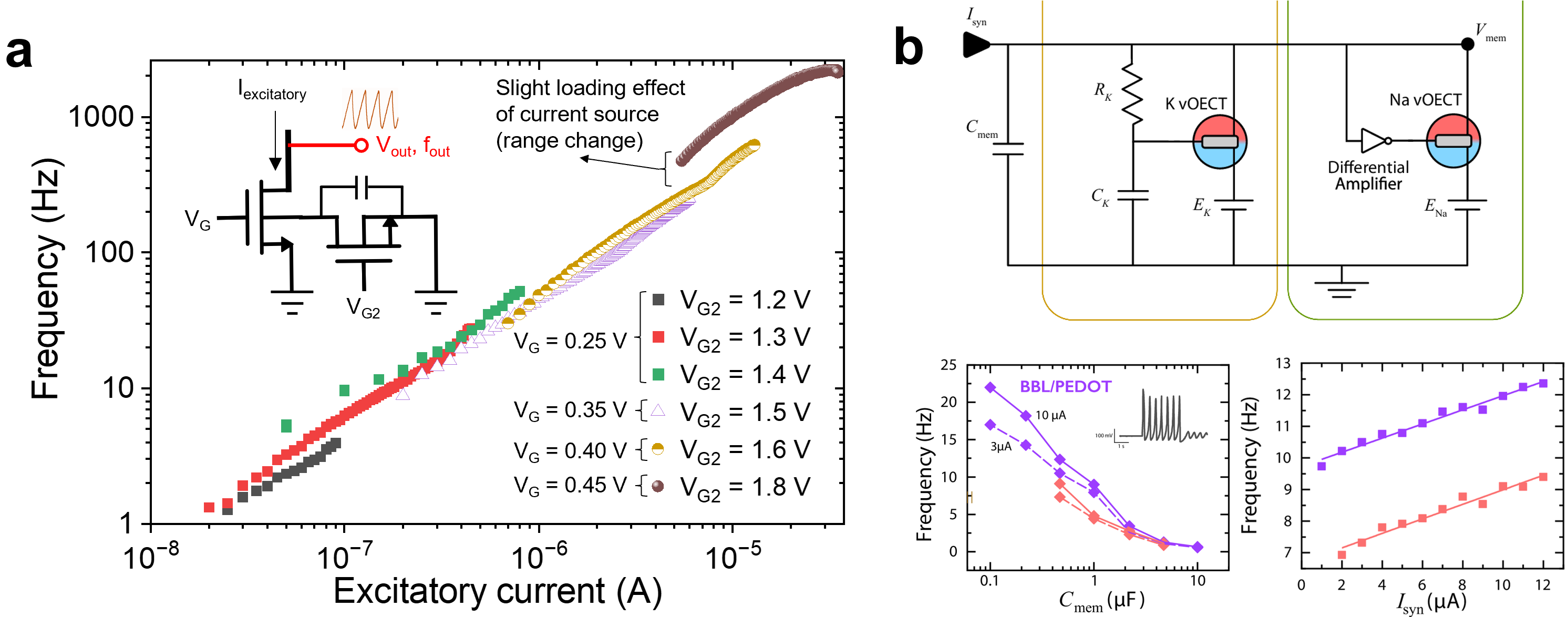


**Supplementary Fig. 31 | Range of firing frequencies across the tuning space and input excitatory currents. a,** The 2-transistor floating bulk neuron device bursts through 3 orders of magnitude in frequency depending on the input current. **b,** Example of state-of-the-art organic transistors operating in the same bursting mode (mimicking the Hodgkin-Huxley model) through a more area demanding circuit, higher currents and larger overall capacitances, making integration difficult. Reproduced with permission from Ref. ^8^. Copyright 2022, Nature Publishing Group

From the technological point of view, an important aspect should be considered when integrating these devices in large arrays. If neurons are expected to be tuned individually, independent body regions are necessary. SOI technologies are excellent candidates for this, as has been shown in other neuron implementations exploiting floating bulk dynamics (see Supplementary Table 1). In bulk silicon technologies, triple wells and deep trench isolation are good alternatives to achieve this, with the corresponding area overhead, but neuron density is less challenging than synaptic array density. In synaptic arrays, many synaptic floating bulk transistors can share a common floating bulk region, but the crossbar addressing circuitry must ensure only the selected devices are being subjected to the writing voltage conditions and rest of the devices connected to the same drain line should be floated to minimize disturbance (half-select) during each cell write. Similar addressing strategies are already common in non-volatile memory arrays employing other charge-trapping technologies, including NAND/NOR Flash ^87^ and charge trapping transistors^77^.


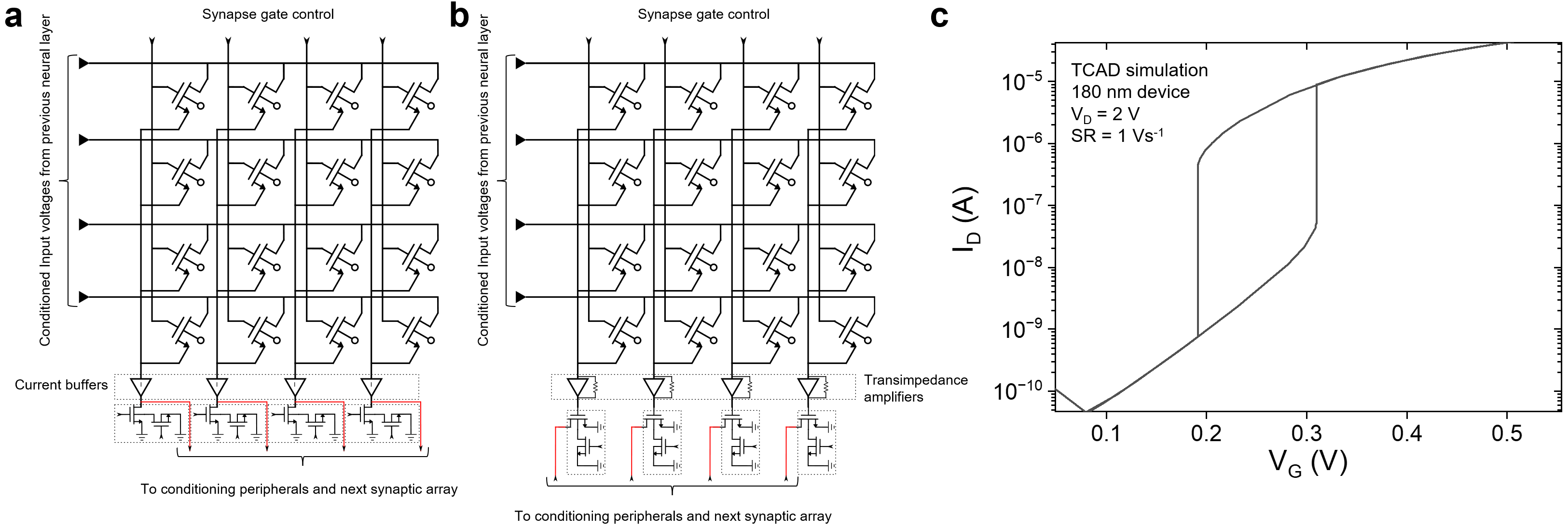


**Supplementary Fig. 32 | Schematic representation of a potential neural network section implemented with floating bulk transistor neurons and synapses.** The array implementation is similar to floating gate or memristor array-based in-memory compute cores. The floating bulk neuron could be operated with **a,** current input, and monitoring its spiking action potential (see Supplementary Fig. 29) or **b,** in voltage mode by using the gate as input (see panel **c**), which would require different peripherals to condition the output for the next synaptic array layer.

The array architecture can be very similar to in-memory compute cores based on floating gate or memristive synapses (see Fig. R15). The peripherals would include array addressing decoders, switches and multiplexers to change the array between training and inference conditions, output sense amplifiers that gather the synaptic crossbar signal and feed it to the output neurons. In general, in between layers of the neural network, buffering amplifiers and signal level shifters or amplifiers are required to condition the signal before is fed to the next layer of neurons through a separate synaptic array. The most convenient architecture for connecting the proposed neuron would be to operate it in current input mode. For this end, each column of the synaptic array could be directly connected to the drain of a neuron cell, although current buffers are recommended (to avoid the neuron loading the output of the synaptic columns). Meanwhile, a voltage buffer can sense the voltage at the neuron to obtain output voltage spikes (as will be discussed further along this letter in the bursting mode). Another alternative is to convert the output of the crossbar columns with transimpedance amplifiers and operate the neuron in voltage mode, requiring a current readout at the output to detect the firing condition. Voltage input mode in the floating bulk neuron is basically driving the gate of the device at constant drain voltage, which will depend on the designed threshold required from the neuron within the neural network. There are several strategies to arrange the peripheral circuits in hardware-based artificial neural networks, some of which are discussed in a recent review^14^ (see an example for a memristive network of 2T2R differential synaptic arrays in Supplementary Fig. 33).


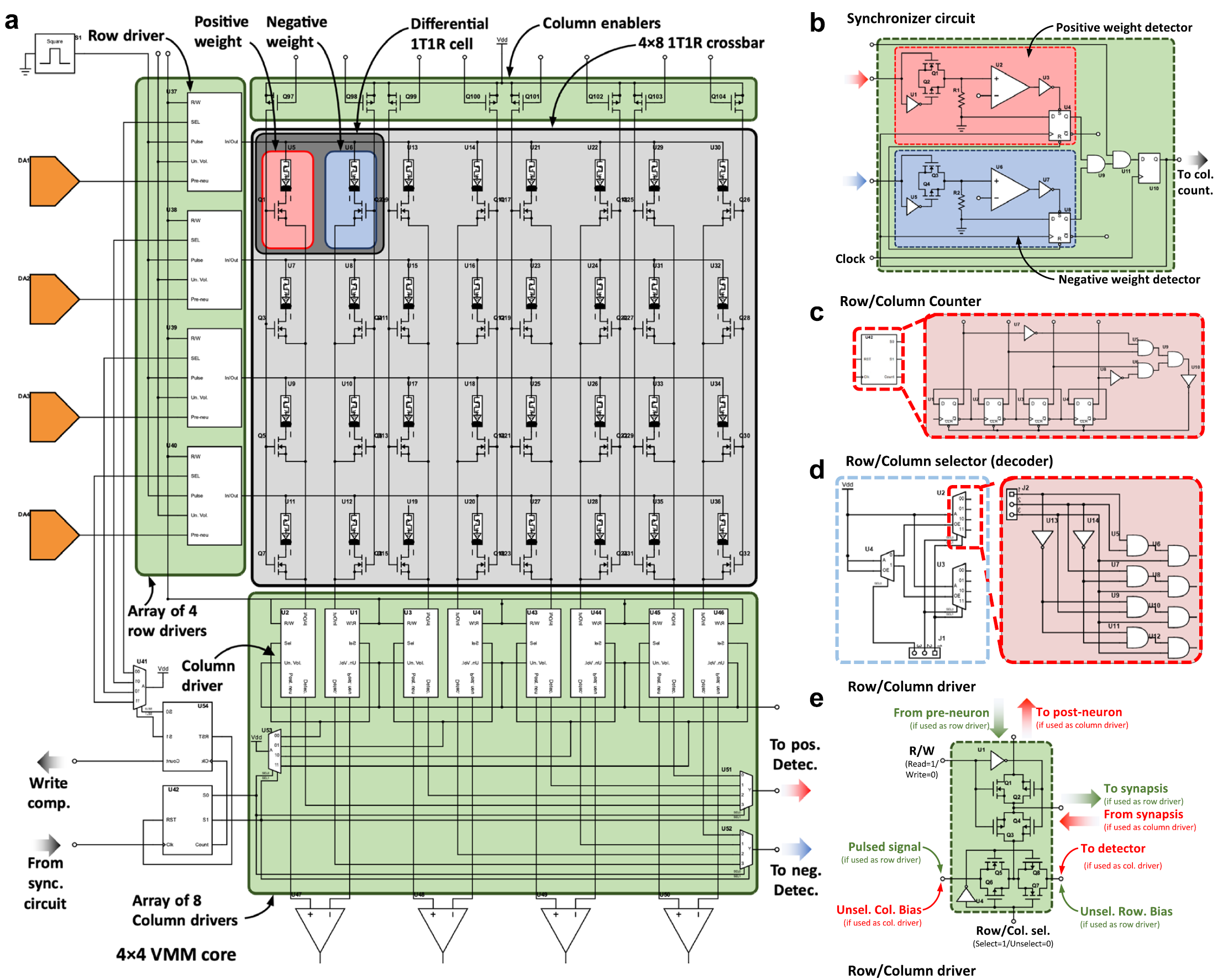


**Supplementary Fig. 33 | Detail of the control circuits used for dual inference/write procedures. a,** Complete circuit schematic for a 4×8 1T1R crossbar array. The 1T1R elements can be replaced by floating bulk synapses. **b**, Detail of the synchronizers including the sense amplifiers used to detect the correct programming of a given memristor. **c,** Address block, essentially a counter which sequentially addresses each memristor in the crossbar. **d,** Row and column decoders, used to enable the memristor addressed by the address block. **e,** Row and column driver, used to bias the rows with the voltage input or with the programming signal, and to connect the columns to the output neurons (during inference) or the sense amplifier (during write-verify). Reproduced with permission from Ref. ^14^. Copyright 2024, Nature Publishing Group.

**
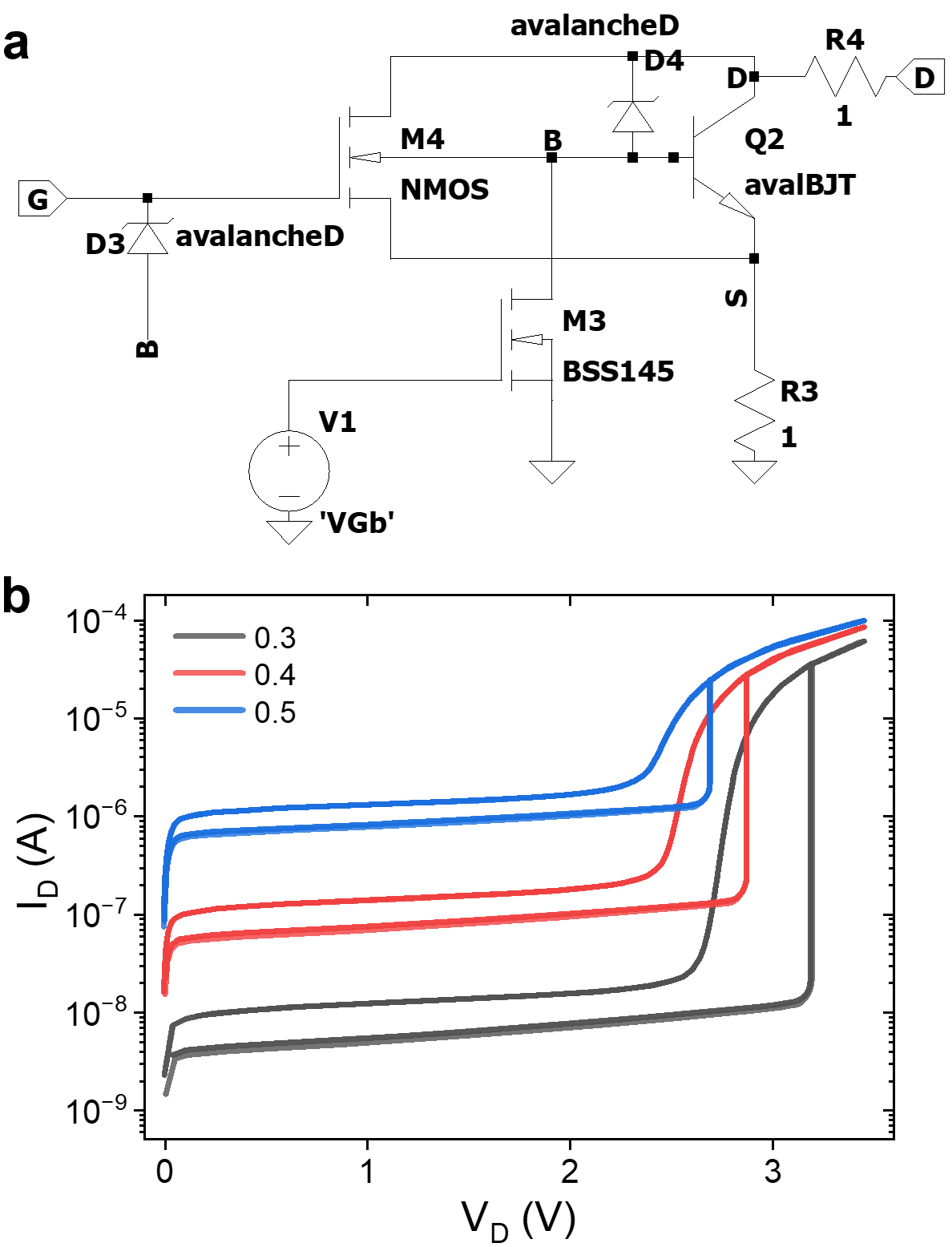
**

**Supplementary Figure 34 | Device level SPICE modelling through fundamental phenomena. a,** Circuit schematic of the floating bulk MOSFET device, with a bipolar transistor (Q2) and an avalanche diode (D4) to model the avalanche mechanism and a body bias control transistor (M3). **b,** Simulation results at three different V_G_, showing good representation of the avalanche mechanism observed experimentally. All simulation netlists and models can be found publicly available in the online repository:

<https://doi.org/10.5281/zenodo.13843362>

### **Supplementary Note 7: Neuro-synaptic mimicking phenomena and devices, and their application in hardware-based demonstrations of ANNs**

Different kinds of ANNs have been proposed based on different device structures and different physical mechanisms employed to implement synapses and neurons. In all cases, for a given switching mechanism, the time between its first use as neuron/synapse and the implementation of a hardware-based ANN was of at least 7 years, and not in all cases these demonstrations have been fully on-a-chip (see Supplementary Table 2). The reason is that moving from single device to ANN still requires an immense engineering work. In all cases, these large neuromorphic systems employ synaptic devices based on standard memory array architectures, that are adapted from common digital or mixed signal circuits. Moreover, a common aspect of all these neural network implementations is that they require a very large amount of custom-designed, dedicated peripheral circuits, interfaces with external circuitry for testing, and several iteration cycles of circuit, block and system level design, fabrication and test before a functional prototype can be achieved. In fact in multiple cases, neuron activations are either implemented with external hardware or even emulated numerically with a system in the loop (FPGA or a PC) which has been extensively discussed this in a recent review^14^. An upside of employing standard widely-used MOSFET transistors to implement electronic neurons and electronic synapses (using punch-through impact ionization phenomenon) should result in much shorter development time towards full hardware implementation of ANNs, compared to the other technologies.

**Supplementary Table 2. Technologies used for neuron and synaptic mimicking.** A summary of the most representative technologies proposed as potential neuron and synapse mimicking devices for ANNs, ant their implementation milestones through time. The second column states the first report of the fundamental physical phenomenon that operates the device. The third column refers to the first experimental demonstration of the capabilities of the device to mimic neurons and/or synapses. The fourth column states the first demonstration of a full neural network, where some of them involve combination of software and hardware (e.g., the synaptic array is generally implemented in hardware and the rest of the neural network is either ran in external hardware or executed in software in a PC). The fifth column highlights the most recent, state-of-the-art neural network implemented with each technology.

| **Technology** | **Fundamental phenomenon reported** | **First report of**  **neuro-synaptic**  **behaviour** | **First full neural network demonstration** | **State-of-the-art neural network demonstration** | **Time from device to system level demonstration** |
| --- | --- | --- | --- | --- | --- |
| Phase change (PCM) | Ovshinsky (1968)  Ref. ^88^ | Kuzum (2011),  Suri (2011)  *(synapse)*  Refs. ^22,89^  Tuma (2016),  Sebastian (2017) *(Neuron and synapse)*  Refs. ^23,33^ | Ambrogio (2018)  *(mixed hardware-software)*  Ref. ^90^ | LeGallo (2023) Ref. ^13^ | 7 to 12 years *(depending on what is considered a full neural network)* |
| Metal-oxide memristors  (RRAM) | Hickmott (1962)  Ref. ^91^ | Snider (2008)  Ref. ^92^  *(synapse spiking time dependent plasticity)* | Prezioso (2015)  *(passive crossbar vector-matrix multiply)*  Ref. ^93^ | Song (2024),  Rao (2023)  Refs. ^94,95^ | 7 years |
| Ferroelectric FET  (FeFET) | Moll (1963)  Ref. ^96^  Ishiwara (1993) *(First concept of neuron + synapse)*  Ref. ^97^ | Mulaosmanovic (2017), Jerry (2017)  *(synapse)*  Refs. ^24,98^  Wang (2018) *(neuron)*  Ref. ^25^ | *Simulations only* | Kim (2023)  Soliman (2023)  *(Simulations only)*  Ref. ^99,100^ | Pending (> 7 years) |
| Magneto-resistive  (MRAM) | Berger (1984)  Ref. ^101^ | Vincent (2015)  *(synapse)*  Ref. ^102^ | Jung (2022) *(vector-matrix multiply)*  Ref. ^103^ | Song (2023)  Ref. ^104^ | 8 years |
| Silicon nanowires |  | Han (2020)  *(LIF neuron)*  Ref. ^37^ | - | Han (2022)  *(simulations only)*  Ref. ^37^ | Pending |
| Punch-through impact ionization / BTBT MOSFET | Moselund (2008)  Ref. ^55^ | Dutta 2017  *(LIF neuron)*  Ref. ^42^ | - | Singh (2022), Kadam (2024)  *(simulations only)*  Refs. ^27,28^ | Pending |
| Charge-trapping Transistor | Kothandaraman (2015) Ref. ^75^ | Gu (2017) *(synapse)*  Ref. ^76^ | - | [Qiao2022]  *(crossbar vector-matrix multiply)* Ref. ^77^ | > 7 years |
| eFLASH / Floating Gate | Chen (1977)  Ref. ^105^ | Diorio (1996) *(synapse)*  Ref. ^106^ | Guo (2017)  Ref. ^107^ | [Wang2023] ^87^ | 21 years |

### **Supplementary Note 8: Physical simulations of the mechanisms governing the floating bulk device**

Technology computer assisted design (TCAD) tools have been used in the past to address the physics behind floating body effects for different technologies, both standard SOI CMOS and other non-standard devices that feature different compatibilities with CMOS processes^32,55,56,68–74^. All these works focused on understanding the mechanisms that give rise to impact-ionization related behaviour in these devices, in some cases for their application as an integration element in leaky integrate-and-fire (LIF) neuron circuits. However, the possibility of using the floating body effect to mimic both neural and synaptic behaviours has not been explored before. Therefore, the physical conditions under which the wide spectrum of neuro-synaptic behaviours here displayed take place, are worth of analysing from the theoretical point of view.

We performed TCAD simulations on a parametric structure of the floating bulk transistor using Synopsys’ Sentaurus TCAD. An overview of the project structure is given in Supplementary Fig. 30. The device structure is defined for simplicity in 2D by the descriptive method using Sentaurus SDE (1). A standard planar N-MOSFET structure is used and parameterized for sensitive design features of the devices under test. Particularly, we used 180 nm channel transistors and 500 nm (thick oxide) channel transistors. To address the architectural differences, we establish structure parameters based on the experimental nominal I_D_-V_G_ of the devices. For 180 nm and 500 nm devices, respectively, best agreement was achieved for: oxide thickness (t_ox_) of 3.5 nm and 10 nm, channel implantation for V_th_ adjustment (chImp) of 1.05×10^18^ cm^-3^ and 1.2×10^17^ cm^-3^ and a channel implantation depth (ImpDepthFactor) of 1.5× and 2× the depth of the source/drain junction diffusion.


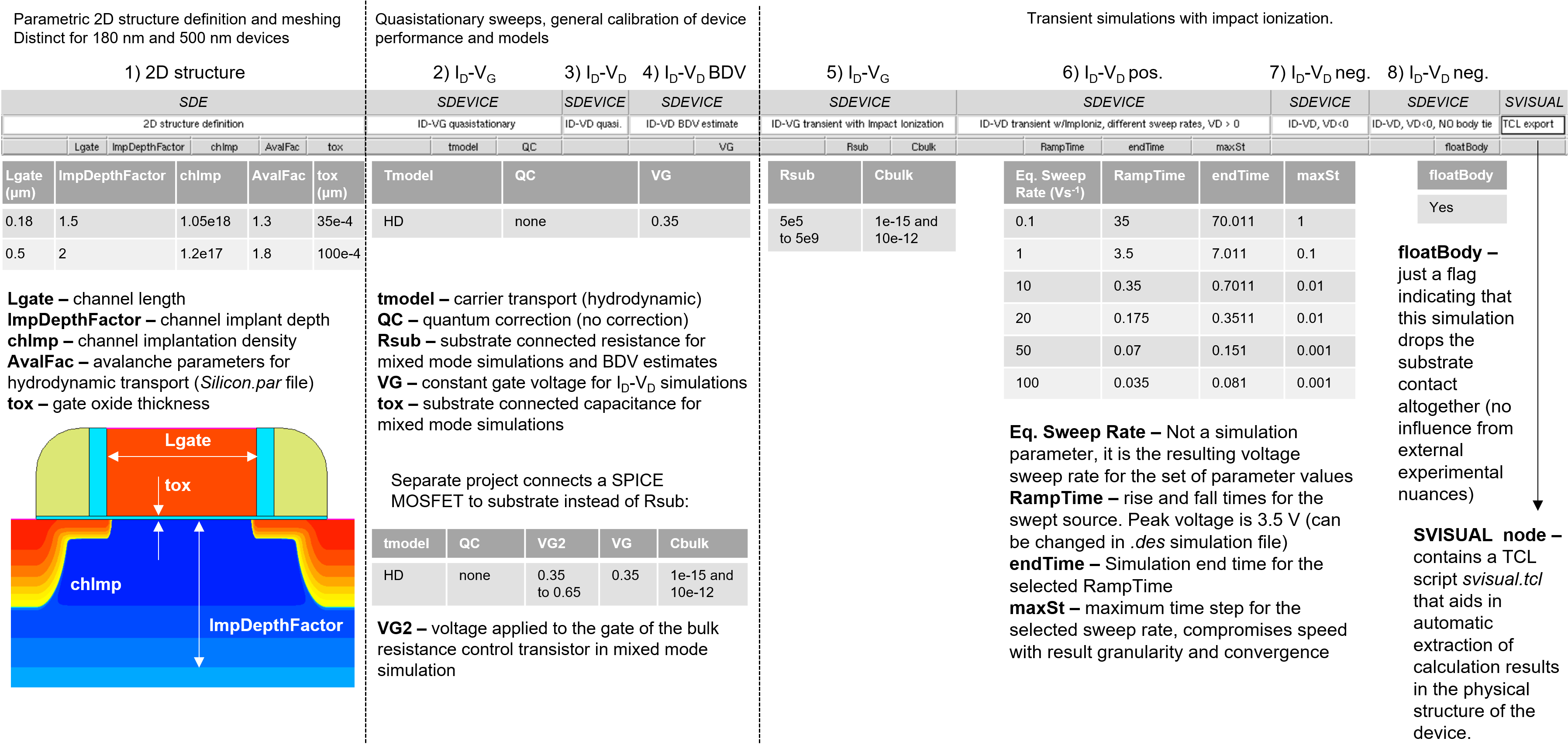


**Supplementary Figure 35 | TCAD simulation workbench and parameters.** A detailed view of the different sections included in the simulation approach of the devices under test. Full project structures are made publicly available (see Methods).

The iterative procedure to calibrate simulation models to experimental data was carried out by performing quasistationary I_D_-V_G_ and I_D_-V_D_ sweeps within nominal operating conditions (bulk grounded and voltages within nominal V_DD_ of 2V and 5.5 V for each device channel length, see Supplementary Fig. 36a,b). For the quasistationary simulations (2) and (3), hydrodynamic transport models without quantum corrections were employed, which are effective for modelling planar sub-micrometer technologies to good agreement. In these simulations, mobility dependences with doping, normal field and velocity are included in the calculations, while only considering Shockley-Read-Hall recombination dynamics (for speed over accuracy). Additionally, an estimation of breakdown voltage (BDV) is carried out using the resistance method (4) while including Auger, band to band and impact ionization models.


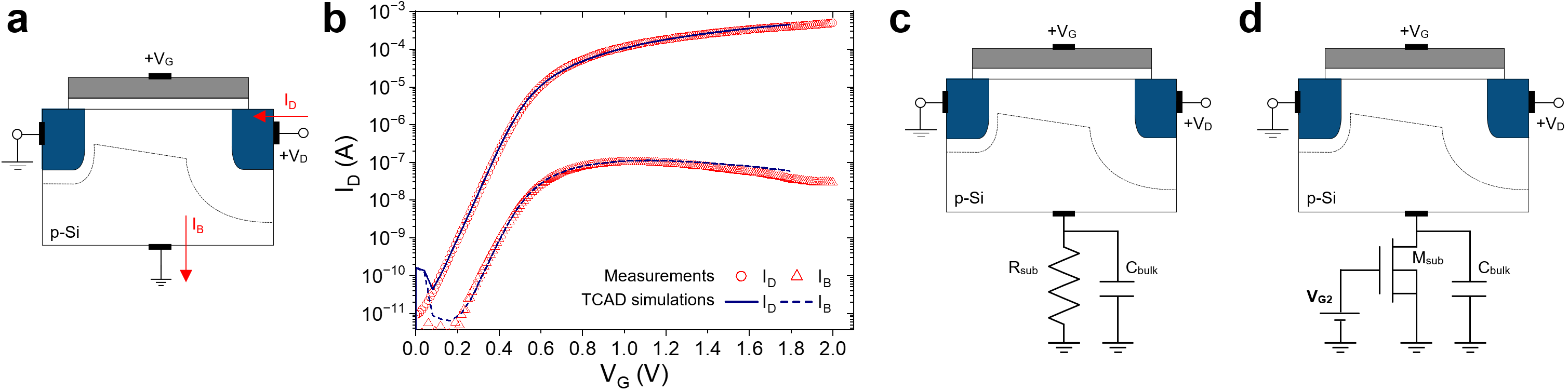


**Supplementary Figure 36 | Agreement of TCAD impact ionization simulations with experimental data.** Fully transient simulations ran at a sweep rate of 1 Vs^-1^ (lines) agreeing well with I_D_ and I_B_ data acquired from the devices under test.

Once these sets of simulations agree with the measurement data, we introduce the transient simulations including Auger (important at high carrier densities), band-to-band (important at high electric fields) and SRH recombination dynamics, and carrier temperature driven avalanche based on the Van Overstaeten model and calibrate the main indicator of impact ionization, which is the bulk current in the devices under test (Supplementary Fig. 36a). Since the driving force in hydrodynamic transport is carrier temperature, we adjust the fitting parameter *λ_n_* in the temperature to field conversion:


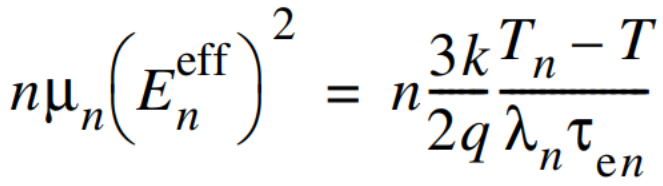
,

where *n* is the density of electrons, *μ_n_* is the electron mobility, *E_n_^eff^* is the effective driving field for the avalanche models, *k* is Boltzmann’s constant, *q* is the electron’s charge, *T* is the room temperature, *T_n_* is the carrier temperature, and *τ_en_* is the electron temperature relaxation time constant within the hydrodynamic transport model equations. An equivalent set of parameters is used for the hole equation, and we used *λ_n_ = λ_p_* in all cases. These parameters were adjusted to 1.3 and 1.8 for 180 nm and 500 nm channels, respectively, obtaining good agreement in nominal operating conditions (see Supplementary Fig. 36b).

With this fit, transient simulations including all of the above models are used within a mixed mode netlist that connects external sources and elements to the structure being solved. This netlist includes a bulk-connected network involving a capacitance (C_bulk_, see Supplementary Fig. 36c and 36d) that accounts for device and setup interconnects, and either a constant lumped resistance (R_sub_, see Supplementary Fig. 36c) or a N-channel MOSFET with BSIM model to control the effective bulk grounding resistance (see Supplementary Fig. 36d). Each of these configurations are provided as separate projects, where the one with the resistance provides a more simplified approach to replicate experimental results while the one with the transistor is more realistic both with the experimental setup and with an actual device application. Independent voltage sources are used for the gate of the main device (V_G_), the gate of the bulk control transistor (V_G2_, if present) and the drain of the main device (V_D_). The sources controlling the bias quiescent point the main transistor – V_D_ in I_D_-V_G_ curves (5), V_G_ in I_D_-V_D_ curves (6) –are ramped to ease convergence of the transient simulation and are immediately followed by the main sweep, which is parameterized to account for different ramp sweep rates in accordance with the experimental conditions.

Finally, a separate set of simulations is included to address the negative V_D_ sweeps used at high R_sub_ (7) or totally floating body (8) conditions (the latter eliminates the inclusion of a substrate contact altogether). These simulations allow us to assess the working conditions of the floating body 500 nm device in the synaptic mode.

### **References for the supplementary information**

1. Kumar, S., Wang, X., Strachan, J. P., Yang, Y. & Lu, W. D. Dynamical memristors for higher-complexity neuromorphic computing. *Nat. Rev. Mater.* **7**, 575–591 (2022).

2. Abbott, L. F. Lapicque’s introduction of the integrate-and-fire model neuron (1907). *Brain Res. Bull.* **50**, 303–304 (1999).

3. Li, C. *et al.* Short-term synaptic plasticity in emerging devices for neuromorphic computing. *iScience* **26**, 106315 (2023).

4. Moraitis, T., Sebastian, A. & Eleftheriou, E. The Role of Short-Term Plasticity in Neuromorphic Learning: Learning from the Timing of Rate-Varying Events with Fatiguing Spike-Timing-Dependent Plasticity. *IEEE Nanotechnol. Mag.* **12**, 45–53 (2018).

5. Yi, W. *et al.* Biological plausibility and stochasticity in scalable VO2 active memristor neurons. *Nat. Commun.* **9**, 4661 (2018).

6. Shao, J., Liu, Y., Gao, D., Tu, J. & Yang, F. Neural Burst Firing and Its Roles in Mental and Neurological Disorders. *Front. Cell. Neurosci.* **15**, (2021).

7. Wang, Y. *et al.* Designing organic mixed conductors for electrochemical transistor applications. *Nat. Rev. Mater.* 1–17 (2024) doi:10.1038/s41578-024-00652-7.

8. Laswick, Z. *et al.* Tunable anti-ambipolar vertical bilayer organic electrochemical transistor enable neuromorphic retinal pathway. *Nat. Commun.* **15**, 6309 (2024).

9. Orchard, G. *et al.* Efficient Neuromorphic Signal Processing with Loihi 2. in *2021 IEEE Workshop on Signal Processing Systems (SiPS)* 254–259 (2021). doi:10.1109/SiPS52927.2021.00053.

10. Whitlock, J. R., Heynen, A. J., Shuler, M. G. & Bear, M. F. Learning Induces Long-Term Potentiation in the Hippocampus. *Science* **313**, 1093–1097 (2006).

11. Fujiwara, H. *et al.* 34.4 A 3nm, 32.5TOPS/W, 55.0TOPS/mm^2^ and 3.78Mb/mm^2^ Fully-Digital Compute-in-Memory Macro Supporting INT12 × INT12 with a Parallel-MAC Architecture and Foundry 6T-SRAM Bit Cell. in *2024 IEEE International Solid-State Circuits Conference (ISSCC)* vol. 67 572–574 (2024).

12. Hsu, H.-H. *et al.* A Nonvolatile AI-Edge Processor With SLC–MLC Hybrid ReRAM Compute-in-Memory Macro Using Current–Voltage-Hybrid Readout Scheme. *IEEE J. Solid-State Circuits* **59**, 116–127 (2024).

13. Le Gallo, M. *et al.* A 64-core mixed-signal in-memory compute chip based on phase-change memory for deep neural network inference. *Nat. Electron.* **6**, 680–693 (2023).

14. Aguirre, F. *et al.* Hardware implementation of memristor-based artificial neural networks. *Nat. Commun.* **15**, 1974 (2024).

15. Sebastian, A., Le Gallo, M., Khaddam-Aljameh, R. & Eleftheriou, E. Memory devices and applications for in-memory computing. *Nat. Nanotechnol.* **15**, 529–544 (2020).

16. Maass, W., Natschläger, T. & Markram, H. Real-Time Computing Without Stable States: A New Framework for Neural Computation Based on Perturbations. *Neural Comput.* **14**, 2531–2560 (2002).

17. Hochreiter, S. & Schmidhuber, J. Long Short-Term Memory. *Neural Comput.* **9**, 1735–1780 (1997).

18. Lont, J. B. & Guggenbuhl, W. Analog CMOS implementation of a multilayer perceptron with nonlinear synapses. *IEEE Trans. Neural Netw.* **3**, 457–465 (1992).

19. Bartolozzi, C. & Indiveri, G. Synaptic Dynamics in Analog VLSI. *Neural Comput.* **19**, 2581–2603 (2007).

20. Pazos, S. *et al.* Solution-processed memristors: performance and reliability. *Nat. Rev. Mater.* **9**, 358–373 (2024).

21. Moon, J. *et al.* Temporal data classification and forecasting using a memristor-based reservoir computing system. *Nat. Electron.* **2**, 480–487 (2019).

22. Suri, M. *et al.* Phase change memory as synapse for ultra-dense neuromorphic systems: Application to complex visual pattern extraction. in *2011 International Electron Devices Meeting* 4.4.1-4.4.4 (2011). doi:10.1109/IEDM.2011.6131488.

23. Sebastian, A. *et al.* Temporal correlation detection using computational phase-change memory. *Nat. Commun.* **8**, 1115 (2017).

24. Jerry, M. *et al.* Ferroelectric FET analog synapse for acceleration of deep neural network training. in *2017 IEEE International Electron Devices Meeting (IEDM)* 6.2.1-6.2.4 (2017). doi:10.1109/IEDM.2017.8268338.

25. Wang, Z. *et al.* Experimental Demonstration of Ferroelectric Spiking Neurons for Unsupervised Clustering. in *2018 IEEE International Electron Devices Meeting (IEDM)* 13.3.1-13.3.4 (2018). doi:10.1109/IEDM.2018.8614586.

26. Milozzi, A., Ricci, S. & Ielmini, D. Memristive tonotopic mapping with volatile resistive switching memory devices. *Nat. Commun.* **15**, 2812 (2024).

27. Kadam, A. A., Singh, A. K., Somappa, L., Baghini, M. S. & Ganguly, U. A Compact Low Power Multi-mode Spiking Neuron using Band to Band Tunneling. in *2024 IEEE International Symposium on Circuits and Systems (ISCAS)* 1–5 (2024). doi:10.1109/ISCAS58744.2024.10557876.

28. Singh, A. K., Saraswat, V., Baghini, M. S. & Ganguly, U. Quantum Tunneling Based Ultra-Compact and Energy Efficient Spiking Neuron Enables Hardware SNN. *IEEE Trans. Circuits Syst. Regul. Pap.* **69**, 3212–3224 (2022).

29. Chen, X., Yajima, T., Inoue, I. H. & Iizuka, T. An ultra-compact leaky integrate-and-fire neuron with long and tunable time constant utilizing pseudo resistors for spiking neural networks. *Jpn. J. Appl. Phys.* **61**, SC1051 (2022).

30. Saha, S., Kanakya, P. B., Goel, M., Baghini, M. S. & Rao, V. R. Energy Efficient Lif Neuron Circuit Using Hybrid Cmos-Nems in 65 Nm Cmos Technology. in *2022 IEEE 35th International Conference on Micro Electro Mechanical Systems Conference (MEMS)* 17–20 (2022). doi:10.1109/MEMS51670.2022.9699762.

31. Kwon, M.-W. *et al.* Integrate-and-fire neuron circuit using positive feedback field effect transistor for low power operation. *J. Appl. Phys.* **124**, 152107 (2018).

32. Chen, Y., Xiao, K., Qin, Y., Liu, F. & Wan, J. A Compact Artificial Spiking Neuron Using a Sharp-Switching FET With Ultra-Low Energy Consumption Down to 0.45 fJ/Spike. *IEEE Electron Device Lett.* **44**, 160–163 (2023).

33. Tuma, T., Pantazi, A., Le Gallo, M., Sebastian, A. & Eleftheriou, E. Stochastic phase-change neurons. *Nat. Nanotechnol.* **11**, 693–699 (2016).

34. Wu, X., Dang, B., Zhang, T., Wu, X. & Yang, Y. Spatiotemporal audio feature extraction with dynamic memristor-based time-surface neurons. *Sci. Adv.* **10**, eadl2767 (2024).

35. Liang, F.-X. *et al.* Stochastic STT-MRAM Spiking Neuron Circuit. in *2020 International Symposium on VLSI Technology, Systems and Applications (VLSI-TSA)* 151–152 (2020). doi:10.1109/VLSI-TSA48913.2020.9203701.

36. Luo, J. *et al.* Capacitor-less Stochastic Leaky-FeFET Neuron of Both Excitatory and Inhibitory Connections for SNN with Reduced Hardware Cost. in *2019 IEEE International Electron Devices Meeting (IEDM)* 6.4.1-6.4.4 (2019). doi:10.1109/IEDM19573.2019.8993535.

37. Han, J.-K., Yu, J.-M. & Choi, Y.-K. A Junctionless Single Transistor Neuron With Vertically Stacked Multiple Nanowires for Highly Scalable Neuromorphic Hardware. *IEEE Trans. Electron Devices* **69**, 3142–3146 (2022).

38. Han, J.-K., Oh, J., Yu, J.-M., Choi, S.-Y. & Choi, Y.-K. A Vertical Silicon Nanowire Based Single Transistor Neuron with Excitatory, Inhibitory, and Myelination Functions for Highly Scalable Neuromorphic Hardware. *Small* **17**, 2103775 (2021).

39. Han, J.-K., Seo, M., Yu, J.-M., Suh, Y.-J. & Choi, Y.-K. A Single Transistor Neuron With Independently Accessed Double-Gate for Excitatory-Inhibitory Function and Tunable Firing Threshold Voltage. *IEEE Electron Device Lett.* **41**, 1157–1160 (2020).

40. Lee, D. *et al.* Various Threshold Switching Devices for Integrate and Fire Neuron Applications. *Adv. Electron. Mater.* **5**, 1800866 (2019).

41. Jerry, M., Parihar, A., Grisafe, B., Raychowdhury, A. & Datta, S. Ultra-low power probabilistic IMT neurons for stochastic sampling machines. in *2017 Symposium on VLSI Technology* T186–T187 (2017). doi:10.23919/VLSIT.2017.7998148.

42. Dutta, S., Kumar, V., Shukla, A., Mohapatra, N. R. & Ganguly, U. Leaky Integrate and Fire Neuron by Charge-Discharge Dynamics in Floating-Body MOSFET. *Sci. Rep.* **7**, 8257 (2017).

43. Dutta, S., Chavan, T., Mohapatra, N. R. & Ganguly, U. Electrical Tunability of Partially Depleted Silicon on Insulator (PD-SOI) Neuron. *Solid-State Electron.* **160**, 107623 (2019).

44. Han, J.-K. *et al.* Cointegration of single-transistor neurons and synapses by nanoscale CMOS fabrication for highly scalable neuromorphic hardware. *Sci. Adv.* **7**, eabg8836 (2021).

45. Woo, S. Y. *et al.* Low-Power and High-Density Neuron Device for Simultaneous Processing of Excitatory and Inhibitory Signals in Neuromorphic Systems. *IEEE Access* **8**, 202639–202647 (2020).

46. Smeys, P. & Colinge, J. P. Analysis of drain breakdown voltage in enhancement-mode SOI MOSFETs. *Solid-State Electron.* **36**, 569–573 (1993).

47. Taur, Y. & Ning, T. H. *Fundamentals of Modern VLSI Devices*. (Cambridge University Press, 2009).

48. Wang, A. Z., Feng, H. G., Gong, K., Zhan, R. Y. & Stine, J. On-chip ESD protection design for integrated circuits: an overview for IC designers. *Microelectron. J.* **32**, 733–747 (2001).

49. Liu, W. Design, Characterization And Analysis Of Electrostatic Discharge (ESD) Protection Solutions In Emerging And Modern Technologies. (University of Central Florida, 2012).

50. Li, Y., Lee, J.-W. & Sze, S.-M. Optimization of the Anti-Punch-Through Implant for Electrostatic Discharge Protection Circuit Design. *Jpn. J. Appl. Phys.* **42**, 2152 (2003).

51. Guegan, G. *et al.* A 0.10 μm buried p-channel MOSFET with through the gate boron implantation and arsenic tilted pocket. *Solid-State Electron.* **46**, 343–348 (2002).

52. Takeuchi, H. *et al.* Punch-Through Stop Doping Profile Control via Interstitial Trapping by Oxygen-Insertion Silicon Channel. *IEEE J. Electron Devices Soc.* **6**, 481–486 (2018).

53. Chen, C.-D. *et al.* Single-transistor latch in SOI MOSFETs. *IEEE Electron Device Lett.* **9**, 636–638 (1988).

54. Cristoloveanu, S., Wan, J. & Zaslavsky, A. A Review of Sharp-Switching Devices for Ultra-Low Power Applications. *IEEE J. Electron Devices Soc.* **4**, 215–226 (2016).

55. Moselund, K. E. *et al.* Punch-through impact ionization MOSFET (PIMOS): From device principle to applications. *Solid-State Electron.* **52**, 1336–1344 (2008).

56. Moselund, K., Ionescu, M. A., Pott, V. & Kayal, M. Capacitor-less memory and abrupt switch based on hysteresis characteristics in punch-through impact ionization mos transistor (PI-MOS). (2009).

57. Boudou, A. & Doyle, B. S. Hysteresis I-V effects in short-channel Silicon MOSFET’s. *IEEE Electron Device Lett.* **8**, 300–302 (1987).

58. Singh Parihar, M., Ghosh, D., Alastair Armstrong, G. & Kranti, A. Bipolar snapback in junctionless transistors for capacitorless dynamic random access memory. *Appl. Phys. Lett.* **101**, 263503 (2012).

59. Lahgere, A. & Kumar, M. J. 1-T Capacitorless DRAM Using Laterally Bandgap Engineered Si-Si:C Heterostructure Bipolar I-MOS for Improved Sensing Margin and Retention Time. *IEEE Trans. Nanotechnol.* **17**, 543–551 (2018).

60. Lahgere, A. & Kumar, M. J. 1-T Capacitorless DRAM Using Bandgap-Engineered Silicon-Germanium Bipolar I-MOS. *IEEE Trans. Electron Devices* **64**, 1583–1590 (2017).

61. Singh Parihar, M., Ghosh, D. & Kranti, A. Single transistor latch phenomenon in junctionless transistors. *J. Appl. Phys.* **113**, 184503 (2013).

62. Ryu, S.-W., Han, J.-W., Moon, D.-I. & Choi, Y.-K. One-transistor nonvolatile SRAM (ONSRAM) on silicon nanowire SONOS. in *2009 IEEE International Electron Devices Meeting (IEDM)* 1–4 (2009). doi:10.1109/IEDM.2009.5424259.

63. Chavan, T., Dutta, S., Mohapatra, N. R. & Ganguly, U. Band-to-Band Tunneling Based Ultra-Energy-Efficient Silicon Neuron. *IEEE Trans. Electron Devices* **67**, 2614–2620 (2020).

64. Dutta, S., Bhattacharya, T., Mohapatra, N. R., Suri, M. & Ganguly, U. Transient Variability in SOI-Based LIF Neuron and Impact on Unsupervised Learning. *IEEE Trans. Electron Devices* **65**, 5137–5144 (2018).

65. Woo, S. *et al.* Implementation and Characterization of an Integrate-and-Fire Neuron Circuit Using a Silicon Nanowire Feedback Field-Effect Transistor. *IEEE Trans. Electron Devices* **67**, 2995–3000 (2020).

66. Han, J.-K. *et al.* Mimicry of Excitatory and Inhibitory Artificial Neuron With Leaky Integrate-and-Fire Function by a Single MOSFET. *IEEE Electron Device Lett.* **41**, 208–211 (2020).

67. Chatterjee, D. & Kottantharayil, A. A CMOS Compatible Bulk FinFET-Based Ultra Low Energy Leaky Integrate and Fire Neuron for Spiking Neural Networks. *IEEE Electron Device Lett.* **40**, 1301–1304 (2019).

68. Han, J.-K. *et al.* Investigation of Leaky Characteristic in a Single-Transistor-Based Leaky Integrate-and-Fire Neuron. *IEEE Trans. Electron Devices* **68**, 5912–5915 (2021).

69. Khanday, M. A., Khanday, F. A. & Bashir, F. Single SiGe Transistor Based Energy-Efficient Leaky Integrate-and-Fire Neuron for Neuromorphic Computing. *Neural Process. Lett.* **55**, 6997–7007 (2023).

70. Rajakumari, V. & Pradhan, K. P. BTBT Based LIF Junctionless FET Neuron With Plausible Mimicking Efficiency. *IEEE Trans. Nanotechnol.* **22**, 172–177 (2023).

71. Rajakumari, V. & Pradhan, K. P. Demonstration of an UltraLow Energy PD-SOI FinFET Based LIF Neuron for SNN. *IEEE Trans. Nanotechnol.* **21**, 434–441 (2022).

72. Kamal, A. K., Thakur, A. & Singh, J. Emulating Switching From Short-Term to Long-Term Plasticity of Bio-Synapse Using Split Gate MOSFET. *IEEE Trans. Nanotechnol.* **21**, 449–454 (2022).

73. Priyanka, Singh, S. & Panchore, M. Dopingless-TFET Leaky-Integrated-Fire (LIF) Neuron For High-Speed Energy Efficient Applications. *IEEE Trans. Nanotechnol.* **21**, 110–117 (2022).

74. Sarkhel, S., Kumari, T. & Saha, P. L-Shaped Double Gate Bipolar Impact Ionization MOSFET Based Energy Efficient Leaky Integrate and Fire Neuron for Spiking Neural Network. *IEEE Trans. Nanotechnol.* **22**, 673–678 (2023).

75. Kothandaraman, C. *et al.* Oxygen vacancy traps in Hi-K/Metal gate technologies and their potential for embedded memory applications. in *2015 IEEE International Reliability Physics Symposium* MY.2.1-MY.2.4 (2015). doi:10.1109/IRPS.2015.7112816.

76. Gu, X. & Iyer, S. S. Unsupervised Learning Using Charge-Trap Transistors. *IEEE Electron Device Lett.* **38**, 1204–1207 (2017).

77. Qiao, S., Moran, S., Srinivas, D., Pamarti, S. & Iyer, S. S. Demonstration of Analog Compute-In-Memory Using the Charge-Trap Transistor in 22 FDX Technology. in *2022 International Electron Devices Meeting (IEDM)* 2.5.1-2.5.4 (2022). doi:10.1109/IEDM45625.2022.10019527.

78. AG, I. T. Embedded Flash IP Solutions - Infineon Technologies. https://www.infineon.com/cms/en/product/memories/embedded-flash-ip-solutions/.

79. Khan, F., Cartier, E., Woo, J. C. S. & Iyer, S. S. Charge Trap Transistor (CTT): An Embedded Fully Logic-Compatible Multiple-Time Programmable Non-Volatile Memory Element for High- k -Metal-Gate CMOS Technologies. *IEEE Electron Device Lett.* **38**, 44–47 (2017).

80. Rauch, S. E. & Guarin, F. The energy driven hot carrier model. in *Hot Carrier Degradation in Semiconductor Devices* 29–56 (Springer International Publishing, 2015). doi:10.1007/978-3-319-08994-2_2.

81. Saks, N. S. *et al.* Observation of hot-hole injection in NMOS transistors using a modified floating-gate technique. *IEEE Trans. Electron Devices* **33**, 1529–1534 (1986).

82. Yoshikawa, K. *et al.* Lucky-hole injection induced by band-to-band tunneling leakage in stacked gate transistors. in *International Technical Digest on Electron Devices* 577–580 (1990). doi:10.1109/IEDM.1990.237132.

83. Ielmini, D., Ghetti, A., Spinelli, A. S. & Visconti, A. A study of hot-hole injection during programming drain disturb in flash memories. *IEEE Trans. Electron Devices* **53**, 668–676 (2006).

84. Pazos, S., Aguirre, F., Palumbo, F. & Silveira, F. Reliability-aware design space exploration for fully integrated RF CMOS PA. *IEEE Trans. Device Mater. Reliab.* **20**, 33–41 (2020).

85. Garba-Seybou, T., Federspiel, X., Bravaix, A. & Cacho, F. New Modelling Off-state TDDB for 130nm to 28nm CMOS nodes. in *2022 IEEE International Reliability Physics Symposium (IRPS)* 11A.3-1-11A.3–7 (2022). doi:10.1109/IRPS48227.2022.9764431.

86. Fox, D. M., Rotstein, H. G. & Nadim, F. Bursting in Neurons and Small Networks. in *Encyclopedia of Computational Neuroscience* (eds. Jaeger, D. & Jung, R.) 1–17 (Springer, New York, NY, 2013). doi:10.1007/978-1-4614-7320-6_454-1.

87. Wang, G. *et al.* A 40nm 5-16Tops/W@INT8 eFlash In-Memory Computing SoC Chip with Noise Suppression and Compensation Techniques to Improve the Accuracy. in *2023 IEEE International Conference on Integrated Circuits, Technologies and Applications (ICTA)* 128–129 (2023). doi:10.1109/ICTA60488.2023.10363786.

88. Ovshinsky, S. R. Reversible Electrical Switching Phenomena in Disordered Structures. *Phys. Rev. Lett.* **21**, 1450–1453 (1968).

89. Kuzum, D., Jeyasingh, R. G. D. & Wong, H.-S. P. Energy efficient programming of nanoelectronic synaptic devices for large-scale implementation of associative and temporal sequence learning. in *2011 International Electron Devices Meeting* 30.3.1-30.3.4 (2011). doi:10.1109/IEDM.2011.6131643.

90. Ambrogio, S. *et al.* Equivalent-accuracy accelerated neural-network training using analogue memory. *Nature* **558**, 60–67 (2018).

91. Hickmott, T. W. Low‐Frequency Negative Resistance in Thin Anodic Oxide Films. *J. Appl. Phys.* **33**, 2669–2682 (1962).

92. Snider, G. S. Spike-timing-dependent learning in memristive nanodevices. in *2008 IEEE International Symposium on Nanoscale Architectures* 85–92 (2008). doi:10.1109/NANOARCH.2008.4585796.

93. Prezioso, M. *et al.* Training and operation of an integrated neuromorphic network based on metal-oxide memristors. *Nature* **521**, 61–64 (2015).

94. Song, W. *et al.* Programming memristor arrays with arbitrarily high precision for analog computing. *Science* **383**, 903–910 (2024).

95. Rao, M. *et al.* Thousands of conductance levels in memristors integrated on CMOS. *Nature* **615**, 823–829 (2023).

96. Moll, J. L. & Tarui, Y. A new solid state memory resistor. *IEEE Trans. Electron Devices* **10**, 338–338 (1963).

97. Ishiwara, H. I. H. Proposal of Adaptive-Learning Neuron Circuits with Ferroelectric Analog-Memory Weights. *Jpn. J. Appl. Phys.* **32**, 442 (1993).

98. Mulaosmanovic, H. *et al.* Novel ferroelectric FET based synapse for neuromorphic systems. in *2017 Symposium on VLSI Technology* T176–T177 (2017). doi:10.23919/VLSIT.2017.7998165.

99. Kim, I.-J., Kim, M.-K. & Lee, J.-S. Highly-scaled and fully-integrated 3-dimensional ferroelectric transistor array for hardware implementation of neural networks. *Nat. Commun.* **14**, 504 (2023).

100. Soliman, T. *et al.* First demonstration of in-memory computing crossbar using multi-level Cell FeFET. *Nat. Commun.* **14**, 6348 (2023).

101. Berger, L. Exchange interaction between ferromagnetic domain wall and electric current in very thin metallic films. *J. Appl. Phys.* **55**, 1954–1956 (1984).

102. Vincent, A. F. *et al.* Spin-Transfer Torque Magnetic Memory as a Stochastic Memristive Synapse for Neuromorphic Systems. *IEEE Trans. Biomed. Circuits Syst.* **9**, 166–174 (2015).

103. Jung, S. *et al.* A crossbar array of magnetoresistive memory devices for in-memory computing. *Nature* **601**, 211–216 (2022).

104. Song, M. Y. *et al.* High RA Dual-MTJ SOT-MRAM devices for High Speed (10ns) Compute-in-Memory Applications. in *2023 International Electron Devices Meeting (IEDM)* 1–4 (2023). doi:10.1109/IEDM45741.2023.10413832.

105. Chen, P. C. Y. Threshold-alterable Si-gate MOS devices. *IEEE Trans. Electron Devices* **24**, 584–586 (1977).

106. Diorio, C., Hasler, P., Minch, A. & Mead, C. A. A single-transistor silicon synapse. *IEEE Trans. Electron Devices* **43**, 1972–1980 (1996).

107. Guo, X. *et al.* Fast, energy-efficient, robust, and reproducible mixed-signal neuromorphic classifier based on embedded NOR flash memory technology. in *2017 IEEE International Electron Devices Meeting (IEDM)* 6.5.1-6.5.4 (2017). doi:10.1109/IEDM.2017.8268341.
